# Forensic, legal, and clinical aspects of deaths associated with implanted cardiac devices

**DOI:** 10.3389/fpsyt.2023.1278078

**Published:** 2023-11-28

**Authors:** Jan M. Federspiel, Stefan Potente, Karen B. Abeln, Kai Hennemann, Sara Heinbuch, Katrin Burkhard, Madita Richl, Mattias Kettner, Constantin Lux, Peter Schmidt, Marcel A. Verhoff, Frank Ramsthaler

**Affiliations:** ^1^Faculty of Medicine, Institute of Legal Medicine, Saarland University, Homburg, Germany; ^2^Department for Thoracic and Cardio-Vascular Surgery, Saarland University Medical Center, Homburg, Germany; ^3^Department of Psychiatry, Clinic for Psychiatry, Psychotherapy and Psychosomatics, SHG-Kliniken Sonnenberg, Saarbrücken, Germany; ^4^Institute of Legal Medicine, University Hospital, Goethe-University of Frankfurt, Frankfurt, Germany

**Keywords:** cause of death, left ventricular assist device, mental co-morbidity in cardiac disease, end stage heart disease, ethics, implanted cardiac devices

## Abstract

As the population ages, the prevalence of heart failure and individuals wearing an implanted cardiac device is increasing. The combination of different underlying pathophysiologies and (the combination of) implanted cardiac devices can become a challenge with regard to the determination of cause and manner of death in such individuals. Additionally, heart disease is frequently associated with mental disease, ranging from anxiety and depression to suicidality and suicide (attempts). At the same time, the correct diagnosis of cause and manner of death is the basis for quality assurance, further therapeutic advances, legal safety, and suicide prevention. By that, an interdisciplinary field between legal medicine, clinicians, and law enforcement opens up. In this field, the different participants can simultaneously benefit from and need each other. For example, legal medicine experts need investigatory results and clinical expertise for the interpretation of readout data of implanted cardiac devices in order to correctly determine the cause of death. A correctly determined cause of death can assist law enforcement and help clinicians to further improve various therapeutic approaches based on correct mortality data collection. In addition, it is the basis for identification of suicides of device carriers, allowing psychological and psychiatric experts to better understand the burden of mental disease in this particular cohort. Against this interdisciplinary background, this manuscript summarizes information about psychiatric comorbidities and suicidality while being on a device. Thereby, basic information on complications and malfunctions of implanted cardiac devices, device-associated deaths with particular emphasis on device manipulation is displayed as basic information needed for correct determination of the cause of death. Also, legal and ethical issues in this field are outlined. The final result is a proposal of an interdisciplinary assessment workflow for a conjoint approach to improve the diagnosis of deaths associated with implanted cardiac devices. It will allow for a differentiation between an individual who died with or due to the device.

## 1 Introduction

Implanted cardiac devices (IcarDs; abbreviations summarized in Supplementary material SA) are well known in the antemortem and the postmortem routine. Many clinical specialties are exposed to IcarDs. For example, cardiologists employ them to treat cardiac arrhythmias (1), or cardiac surgeons implant total artificial hearts to bridge a patient to transplantation (2). Also, palliative care specialists encounter them during end-of-life decisions (3, 4). In the postmortem field, IcarDs can be employed to estimate time since death (5, 6), identify unidentified bodies (7, 8), or to clarify the cause of death (5, 9). Additionally, the legal medicine experts contribute to the work-up of IcarD-associated deaths [e.g., (10)] including IcarD associated suicides [e.g., (11)]. However, both fields are facing increasing numbers of IcarD carriers [e.g., (12, 13)]. This could be attributed to the accelerated aging of the 21st century’s population (14). At the same time the incidence of heart failure (HF) is increasing (12, 13). This would fit the growing spectrum of IcarDs complementing the pharmaceutical HF therapy (15, 16). Thereby, for both fields the differentiation whether a person died with an IcarD, or due to an IcarD is relevant. For the clinicians this information is an important outcome measure, and by that, fundamental for further improvement of patient care in terms of safety and quality (17), like demonstrated for HF (18). For the legal medicine expert, clarification of the exact cause of death is a central task to support law enforcement [e.g., (19, 20)]. This is also relevant in cases dealing with malpractice allegations, especially considering its increasing number in the field of internal medicine/cardiology from 2020 to 2021 in Germany (21). In this context, it has to be pointed out, that it is not the task of legal medicine to assess indication, quality of the preimplantation diagnostics, and so forth. Therefore, pertinent clinical experts must be consulted. Also, in cases with assumed deaths due to assumed device malfunction an exact diagnosis of both, the cause and manner of death, is crucial. Respective cases are non-scientifically published (22), and consecutively devices has been withdrawn by the United States Food and Drug Administration (FDA) (23). However, in the ante-and the postmortem setting, the correct differentiation between death with or due to an IcarD is necessary to correctly fill the death certificate. This again has general legal implications (24) such as the opening of a death investigation. But although important, this differentiation is far from trivial. This can be attributed to several factors. One is the complex pathophysiology of HF (summary see Supplementary material SB) and its high mortality (16). Additionally, there is a broad spectrum of IcarDs available nowadays (summary see Supplementary material SC). The author group made these observations on the occasion of two cases reported in Supplementary material SD. In both cases a left ventricular assist device (LVAD) was employed to commit suicide.

Because two cases of suicide by LVAD prompted the present narrative literature review, psychiatric co-morbidity as one cause of suicide is first discussed below. Subsequently, topics that are indispensable for the correct classification of the cause of death will be dealt with. These are, complications, mortality, and device readout as a source of information. Afterwards, further legal and ethical aspects such as the classification of the manner of death are discussed. Finally, a proposal for an assessment workflow of deaths associated with IcarDs is presented.

## 2 Materials and methods

The present article mostly resembles a narrative review article. A particular focus on IcarD associated suicidality is applied. Thus, the outline of the current knowledge of mental illness and suicidality in device carriers is based on a systematic literature search. Therefore, the data base PubMed® was searched. The search algorithms employed are given in the respective paragraph. The identified literature was selected by screening title and abstract. An article was selected, if it dealt with suicide in association with an IcarD. The summary results of each data base query are provided in Supplementary material SE.

## 3 Psychiatric comorbidity of cardiac devices and heart failure

In order to discuss suicide of IcarD carriers, first the question of why these individuals commit suicide will be addressed. Afterwards, the different devices are revisited regarding there associated mental burden. Overall, the suicidality seems attributable to the immense burden of mental co-morbidity in heart disease.

First it must be highlighted, that “cardiac patients” in general show increased depression rates. A clinical trial of coronary artery disease and arterial hypertension patients demonstrated, that at least 14% of the patients had suicidal thoughts within the past 2 weeks. This trial also points out, that the mental health state in their cohort seems to be not attributed to the heart disease only. For example, they found associations of suicidality with loneliness, retirement and stroke in their cardiac patients. They, also found females to have more frequent suicidal ideations compared to males (25). Taken together, this study underlines the relevance of the biopsychosocial model and the associated transdisciplinarity (26).

This trend towards suicidality is known since decades. For example, in a long-term study in 1986 of individuals after correction of tetralogy of Fallot, suicide was observed as a leading cause of death (27). Especially after cardiac events such as acute myocardial infarction, the suicide rate peaks (28). Subsets of cardiac patients of particular relevance for the present review are HF patients and IcarD carrier.

HF patients are exposed to psychological distress (16), considered as overall severe burden of mental disease (29, 30). This includes depression and anxiety disorders in particular (31). In general, up to 30% of individuals suffering from HF experience depression (32). Even higher rates of depression, up to 70%, have been reported in individuals with end-stage HF (29). Among these, depressed HF patients have worse clinical outcomes compared with non-depressed HF patients (32). Correspondingly, psychological comorbidity in HF has been associated with disease progression (31). Also, readmission rates after the procedure of depressed individuals undergoing LVAD implantation are reportedly higher (33). Approximately 2% of the LVAD recipients have attempted or completed suicide (11).

Carrying an IcarD in general seems to be associated with mental illness also. For example, carrying an implantable cardioverter/defibrillator (ICD) or pacemaker (PM) has been associated with anxiety and depression (34). PM patients show depression rates as high as 45% (35). ICD carrier present with depression rates of approximately 23% (34). Even adolescents carrying an ICD present with anxiety and/or depression (36).

Depression progression after ICD implantation has been observed in approximately 23.6% of the affected individuals (37). The depression rates are reported to be significantly higher in ICD carriers compared to PM carriers and a control group (34). As ICDs can be indicated in HF (16), this could be attributable to the HF patients among the ICD carriers. The patients often name a “mental vacuum” (38) as their reason for suicidality. Interestingly, not only the IcarD carriers, but also their partners exhibit psychological distress. For ICD patients it has been shown that patients’ partners also show elevated depression and anxiety rates (39).

In the given context, one must also consider that physically ill individuals are significantly more likely to choose nonviolent suicide (40). So, IcarDs could be seen as a nonviolent option to commit suicide. From a legal medicine perspective, therefore, they must be considered “potential kill switches“. This means that manipulation of its soft- or hardware could potentially cause someone to “die” (41).

### 3.1 Pacemaker

For PMs, 85 publications were identified [last database query: 3rd May 2023; 5:45 pm; search algorithm: (suicide) AND (pacemaker)]. Of these, 9 publications dealt with suicide in PM carriers (35, 42–49). The other articles mostly dealt with the therapeutic use of pacemakers in the treatment of individuals after they have attempted suicide by various substances such as thioridazine (50), nebivolol (51), or propafenone and captopril (52). Also, ethical aspects in end-of-life-scenarios [e.g., (53, 54)], or therapeutic aspects in accidental digoxin overdosing (55) are discussed in the literature. However, 8 articles deal with suicide attempts by targeting the aggregate and/or the leads (35, 42, 43, 45–49). In one case report, the individual attempted suicide by jumping out of the window after PM implantation (35). In some instances, the individuals combine the PM removal attempt with intoxication [e.g., (43, 46, 48)]. Only in one reported case no mental illness was known prior to the suicide attempt (35). In all remaining cases, mental illnesses were prior known (42–49). No literature on a possible association between cardiac resynchronization therapy (CRT) and suicide could have been identified [last database query: 4th May 2023, 03:00 pm; search algorithm: (suicide) AND (cardiac resynchronization therapy)]. The algorithm found 2 articles on sudden death in general (56) and ethical issues of ICD withdrawal (57).

### 3.2 Implantable cardioverter/defibrillator

A literature search regarding ICDs identified 27 publications [last database query, 4th May 2023; 11:30 am; search algorithm: (suicide) AND (implantable cardioverter defibrillator)]. One article was identified describing a case involving a suicide attempt by medication overdose due to phantom shocks (58). Two cases are reported in which the ICD aborted suicide attempts. One of these describes, that the ICD terminated a self-electrocution-induced ventricular tachycardia (59). The other article describes an individual who attempted suicide by overdosing “diazepam, coumadin, and other pills” (60). Again, the ICD aborted the suicide attempt (60). Furthermore, a report was found describing an individual who had ventricular tachycardia due to a myocardial scar. This was caused by a bullet during a suicide attempt 28 years ago (61). The ICD was used to treat the consequences of this failed suicide attempt (61). No other articles on ICD associated suicide could have been found. The vast majority of articles found by the algorithm addressed ethical and legal aspects of device deactivation in end-of-life-scenarios [e.g., (62)].

### 3.3 Mechanical circulatory support

Corresponding to the cases reported in Supplementary material SD, in this section particular focus on ventricular assist devices (VADs) is applied. For mechanical circulatory support (MCS) in general 18 articles have been identified by the algorithm [last database query, 4th May 2023; 12:20 pm; search algorithm: (suicide) AND (mechanical circulatory support)]. For VAD specific, 22 articles have been found [last database query, 4th May 2023; 12:25 pm; search algorithm: (suicide) AND (ventricular assist device)]. The results of the algorithms overlapped [e.g., (3, 63, 64)]. Most articles identified dealt with ethical and/or legal aspects in end-of-life-scenarios.

For MCS, no article was found describing a suicide or suicide attempt on a device. Instead several articles describe MCS-systems used to treat people after pharmacological suicide attempts. Thereby, cases with the use of an extracorporeal membrane oxygenation (ECMO) (63, 65, 66), so-called extracorporeal cardiopulmonary support (ECHLS) (67), or intra-aortic balloon pump (IABP) (68) are reported. However, in one author’s experience (KH), at least one suicide was completed by a patient on the so-called “awake ECMO.” This term describes cases in which the patient is awake while on ECMO (69, 70). In this case, the patient removed one of the cannulas. Thus, there is a paucity of literature on the description and/or assessment of an association between MCS systems and suicide.

Regarding VADs, there are two case reports of suicide (attempts). In one, a depressed patient committed suicide by disconnecting its LVAD’s drive line (71). The other case report describes a suicide attempt of a LVAD carrier by overdosing zolpidem (72). This individual is reported to have attempted suicide because he felt that all the trouble associated with receiving his LVAD was not worth it due to the restrictions and limitations imposed by the recent severe acute respiratory syndrome corona virus 2 (SARS-CoV-2) pandemic (72). Also, two observational studies has been identified. One of these reports on severe psychological distress in VAD carriers (73). Suicidal ideation and anxiety were observed in 17.5% of the individuals. Depression was found in 14.9% of the patients (*n* = 121) (73). Another study reported that 10 of 494 LVAD patients (approximately 2%) completed or attempted suicide during an 18-month follow-up period after hospital discharge following LVAD implantation. Suicide was completed in eight cases. Either the driveline was disconnected, respectively, cut, or an intoxication was caused. Unsuccessful attempts were found in two instances (1 attempt by targeting the drive line, 1 attempt by intoxication). Nine of these individuals were men. Only 2 of these 10 had a known psychiatric comorbidity. Of these 10 individuals, three did not undergo psychiatric evaluation before LVAD implantation (11).

Additionally, an article has been found, that describes the sociocultural risks of LVAD carriers in the Middle East. This region appears to have a high incidence of HF. Here, advanced HF occurs much earlier in life. Therefore, LVADs are used as bridge-to-destination, as heart transplantation is rarely available. At the same time, this region struggles with terrorism and suicide bombers. The article describes several cases in which LVAD carriers were mistaken for suicide bombers due to the belt, batteries, and drive line. Some were even at risk of having their drive line cut after a car accident as they were mistaken as suicide bombers (74).

### 3.4 Summary: mental disease and suicidality in implanted cardiac devices

Reviewing the association of various IcarDs with suicide, it appears that most case reports are available for PM carriers, whereas the best evidence with observational studies is available for LVAD patients. Nevertheless, the literature seems to be incomplete so far regarding the association of other-than-VAD-MCS systems and suicide.

Cases, analogous to the two reported cases (Supplementary material SD), are reported in which suicide was committed by “pulling the plug.” Thus, at least, LVADs can be considered potential “kill switches.” This emphasizes the importance of a pre-implantation psychological/psychiatric assessment. The results of the literature search indicate, that a respective assessment is mandatory in suicide prevention. The literature findings underpin, that not only individuals prior to VAD implantation (16) but also individuals undergoing implantation of other IcarDs should undergo psychosocial assessment (43). Interestingly, the literature findings showed that LVADs and PMs are targets of suicide (attempts), whereas ICDs rarely seem to be associated with suicide. The ICD was in most cases somehow “beneficial.” Contrasting, PMs’ aggregates and/or leads were the target or trigger of the respective suicide attempt. This aligns with the understanding of ICD carriers. Generally, they rate their IcarD as beneficial (75). It could be speculated that this is due to the preventive character ICDs have. The arrhythmias they can terminate are in turn associated with psychological and physical stress (76). PM carrier targeting their aggregate or the leads, also contrast with LVAD carriers. Latter generally preferred more non-violent attempts by intoxication and/or unplugging. Following this line of thought, Rosenthal and colleagues raised the question as early as 1980 whether the PM devices and leads should or could somehow be concealed from their carrier (47). From this perspective, leadless PM technology – originally developed to prevent lead-associated complications (77) – seems useful in the prevention of suicide attempts as well.

While knowledge on the associated psychiatric co-morbidity is fundamental for understanding the why, knowledge on device associated complications, malfunction and mortality is the fundamental for the correct diagnosis of device associated suicide.

## 4 Complications

Foremost it must be stated that the complications associated with IcarDs are “case- and device specific.” This is due to differences in the underlying pathophysiology, the characteristics of different devices, and others. However, different clusters of complications can be distinguished such as *“surgical complications”* (e.g., bleeding or right heart failure) (78) and *“medical complications”* (e.g., ischemic neurological events) (79). For LVADs it can be additionally distinguished between LVAD specific complications, such as device malfunction, and LVAD-associated complications, like aortic regurgitation (80). Also, complications can be assigned to several groups at the same time, such as infection or pump thrombosis (78–80). The group of “unusual complications” such as late onset aortic regurgitation, uterine bleeding, or display malfunction (81) is described also. Regardless of how these complications are grouped they bear a risk for the IcarD carrier. For example, a common factor of LVAD complications in general is their high mortality and morbidity (80). Besides such general clusters, there are also typical complications for each device. So, is hemolysis quite frequently in continuous flow VADs (82). Or acute lung injury (83) is considered typical for extracorporeal life support systems (ECLS). Besides the individual and the device, also the time frame has to be taken into consideration. This is due to different complications being frequent in the early (i.e., implantation, early postoperative phase), and the late phase. In the following, this will be explained using the PM as an example. Here, the most common complications shortly after implantation are pneumothorax, hematoma, lead dislodgement, or superficial phlebitis (84). In this context, especially “re-do procedures” such as PM upgrade (e.g., adding leads) may be associated with higher complication rates compared to initial interventions (85). In the late phase, especially lead-related issues (e.g., lead fracture), endocarditis, or PM-induced HF are more relevant (86). In particular, surgeon experience and patient morbidity seem to influence the long-term complication rate (87). In addition to surgical experience, the technique applied plays a pivotal role. For example, in the past, redundant leads were often abandoned (88). Contrasting, recent studies demonstrated that lead extraction can be performed safely with the laser technique (88). Summarizing, for the PM, the early phase tends to be dominated by surgical/procedural complications (e.g., pneumothorax), whereas the long-term phase tends to be dominated by device-and lead-associated complications (e.g., HF, endocarditis). A brief summary of complications associated with the different IcarDs is given in Supplementary material SF.

## 5 Malfunction

Talking about technical devices, malfunction must always be considered. For the IcarDs the cause of malfunction can be in the device itself (e.g., PM battery depletion), in the connection to the heart (e.g., PM lead fracture), and in the heart (e.g., increased pacing threshold) (89). A particular issue with the device itself is its so-called “programming” (90) or software (91). Programming describes the settings that clinicians make using software to make the device work according to agreed parameters. Cases have been reported in which PM firmware updates resulted in interrogation malfunction (92). So, particular attention should be paid during interrogation after a firmware update, especially for PM-dependent individuals (92). Although such updates carry a particular risk, they are still necessary. One reason therefore is the susceptibility of such devices to cyberattacks. Here appropriate updates can improve the safety (93). But, also external factors can lead to device malfunction. For example, PM malfunction can occur due to electrocautery during surgery (94) or due to radiotherapy (95). Depending on device and patient, the impact of device malfunction differs. For example, in PM carriers, malfunctions are usually not fatal (96) and can result in symptoms such as dizziness or chest pain (97). On the other hand, there are devices such as LVADs in which malfunction is more likely to be fatal (98). It should be noted that LVAD malfunction involves much more than just the pump, but also includes failure of the controller, battery, or patient cable, among others (98). Interestingly, in LVADs, patient non-compliance does not appear to be associated with device malfunction (98).

## 6 Mortality

Mortality varies from case to case due to different underlying diseases, the procedure chosen, the device used, and so on. Therefore, each case in legal medicine casework that addresses the question of IcarD-associated death requires a case-specific evaluation. This must take into account as much information as possible, such as situation of finding the body, signs of extraneous involvement, autopsy findings, and the results of subsequent further analyzes (e.g., histology), as appropriate. Additionally, consultation of appropriate experts such as perfusionists or cardiac surgeons, has to be considered to clarify specific issues at hand. One issue could be the device readout and its interpretation. Thereby, the choice of the expert must make sure that in the respective case there is no conflict of interest. For example, in the legal medicine setting, the cardiac surgeon who did the assessed and disputed surgery, is not appropriate. In the experience of the author group, it has proven useful to consult higher-level entities, such as the manufacturer or the coordinator of the respective VAD program. This can help to find appropriate experts without conflict of interest and also provide equipment for reading out the information.

How mortality can vary is illustrated in the following for the MCS setting. In short-term MCS treatment, 54% of all individuals died on the device (99). Contrasting, long-term MCS therapy as bridge to destination, can be associated with a two-year survival rate of up to 62% (100). Also, how the devices are installed has to be considered. For example, there are several ways to implement an ECLS. They can be installed through an open chest to allow for larger cannulas with central cannulation of the right atrium and the aorta (101). Or they can be installed via the femoral arteries and veins (102). Overall, ECLS has been associated with in-hospital mortality rates of up to 55.5% (103). Herein, associated pathological changes such as need for blood transfusion can lead to higher mortality (103). With the open chest approach being associated with higher transfusion rates (104), the implantation technique may influence the mortality rate observed.

## 7 Readout and associated problems

The data obtained by device readout may be useful for reconstructing events prior to death (10). However, the readout can also raise new questions, as illustrated by the case study by Junge et al. (10). They found a magnetically reversed ICD in a 66-year-old HF patient. As well, 5 ICD interventions in the last 4 days before death has been found in the memory (10). For this reason, the authors doubted the “naturalness” (10) of death in this particular case. The authors state that in a case without readout and without autopsy, the cause of death in ICD carriers “must be reported as unknown” (10). This indicates that device readout does complement the autopsy, for example, by allowing for detection of magnetically reversed devices (105). This cognition, facilitates to evaluate the role of the reversed device in the causal chain leading to death.

## 8 Device manipulation

It is important to note, that device manipulation can occur in various occasions, such as end-of-life decisions or intentionally for suicide. Additionally, plenty external factors can influence or manipulate the device. So, the device can become electrically damaged (106), can dislocate (107) or a cannula can be accidentally removed (106). By that, IcarDs can complicate the determination of the manner of death, especially since there can be discrepancies in the interpretation of deaths between clinicians and (forensic) pathologists (10). In a series examining IcarD data obtained before cremation, single cases with magnet reversion were identified (105). It was not possible to clarify whether this was performed by clinicians because the end of life had been reached or resembled suicide or homicide (105).

### 8.1 Implanted cardiac devices and magnets

Connecting to the aforementioned series, it must be highlighted, that IcarDs can not only be manipulated by magnets, but that the magnet can also damage the data stored on the device (10). At this point, it is important to note that not all magnets *per se* bear the potential to manipulate an ICarD. The magnet must have a particular strength. For example, it has been demonstrated that the magnetic field of a dermatoscope is not capable of influencing IcarDs (108). Furthermore, it should be noted that even stronger magnetic fields, such as those encountered during magnetic resonance imaging (MRI), do not necessarily lead to severe or even fatal changes in IcarD function (109, 110). Since safety regarding the magnetic field in MRI depends on an interdisciplinary approach and appropriate precautions (111), the unconscious magnetic manipulation of an IcarD can lead to potentially severe adverse events for its carrier even in a clinical setting (112). Hereby, it must be noted, that some everyday technologies, such as certain phones, can cause magnetic fields strong enough to turn IcarDs into magnetic mode, as indicated in a 2021 FDA notification (113). At least in the recent years, a rapidly growing number of smartphones has been reported (114), along with an increasing number of users with more than one device (115). Also, elderly use such handheld devices (116) and smartphones are even being investigated in Alzheimer’s Disease therapy (117). Therefore, accidental magnetic manipulation of IcarDs must also be considered. This points out the need for a thorough postmortem examination along with the analysis of perimortem circumstances. Such “unintentional” magnetism is also relevant in a clinical setting. The HeartMate 3 LVAD exhibits a fully magnetically levitated rotor. Its magnetic field can interfere with the function of various ICD models (118). Therefore, a safety margin of at least 10 centimeters should be maintained during the implantation of the LAVD and/or ICD (118).

Although devices can be magnetically manipulated, a literature search failed to find a report of suicide by magnetic device manipulation (Data base: PubMed^®^; search terms: (suicide) AND (magnet) AND (pacemaker); last query 10th January 2022). This is somehow surprising considering the prior outlined suicidality of “cardiac patients,” and that patients are informed regarding the interference of their device and magnets (112, 119). This raises the question of whether there are no cases of completed suicide involving magnet application or whether they are simply not detected. However, there is a case report of a medical professional who used defibrillator pads to commit suicide by self-electrocution (120). This underlines that “knowledgeable” individuals can abuse their knowledge to commit suicide.

### 8.2 Effects of device manipulation

**Figure 1:**
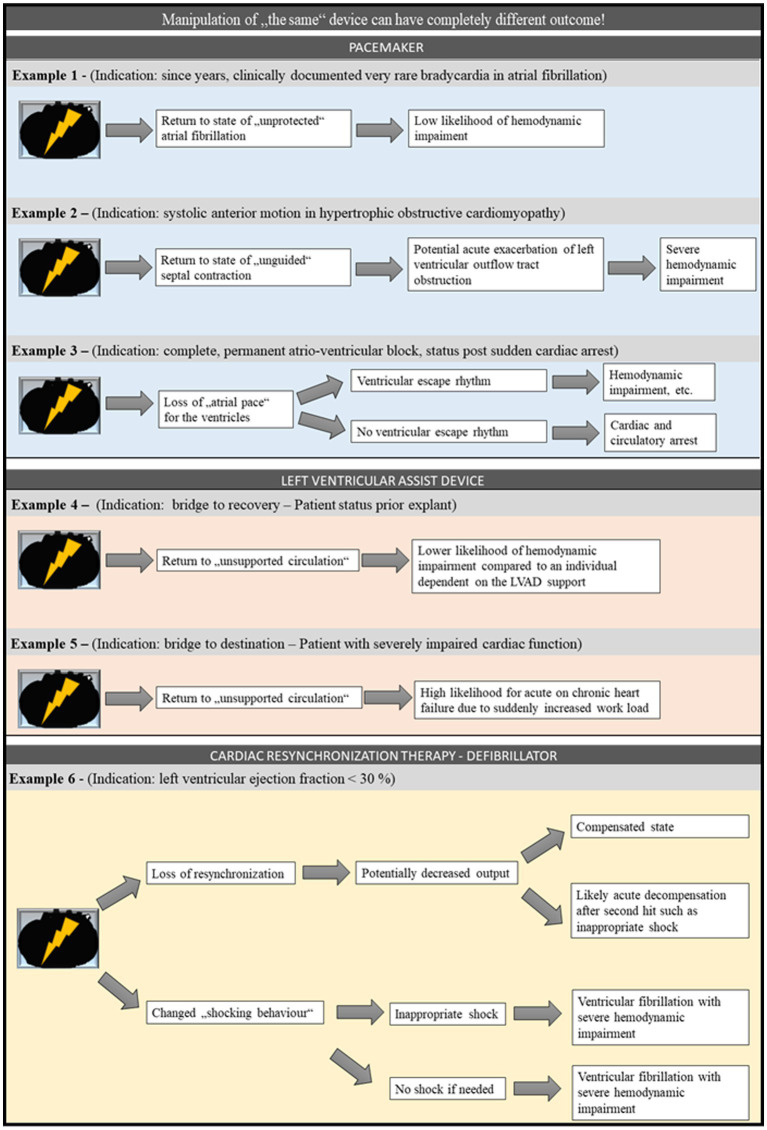
Example for potential consequences of loss of function of different cardiac devices. For PM, LVAD, and CRT-D, different potential scenarios starting with a loss of function of the respective device are displayed. It is highlighted that although the starting point is the same in different scenarios, each scenario reaches a different endpoint and in some cases may even reach different endpoints within a given scenario. CRT-D, cardiac resynchronization – defibrillator; LVAD, left ventricular assist device; PM, pacemaker.

The effect of turning off or manipulating a device is not uniform again. Instead, the effects are highly dependent on the device type and manufacturer, the underlying disease, and the current disease state, concomitant diseases and therapies, etc. This is exemplified in the following for the PM (Figure 1). First, the area of underlying disease and thus PM-dependence is addressed. PM-dependence can be defined as a high risk of “serious injury or death from sudden pacemaker failure” (121). For example, if the PM was indicated due to atrial fibrillation with bradyarrhythmic episodes, that individual may have an apparently high probability of surviving without the device during non bradyarrhythmic phases. In contrast, if this individual is PM-dependent in permanent complete atrioventricular-block, sudden device failure can be fatal. This has already been highlighted in a case report of an individual with sick sinus syndrome who died due to PM malfunction (9). Overall the prevalence of PM-dependence is comparably low. A clinical cohort study reports approximately 2% of the analyzed individuals to be PM-dependent (121). These clinical data fits with observations in the postmortem setting. A study analyzing PMs and ICDs explanted before cremation showed that approximately one third of the explanted devices had low or no power (105). Nevertheless, knowledge of the indication for device implantation and the current disease state is necessary to assess the role of an IcarD in the causal chain leading to death. Next, the aspect of the different devices is displayed for magnetic device manipulation. Plenty manufacturers offer a variety of different PM devices (119). It has been reported that, except for Sorin PM, removal of a magnet results in reversal to the usual preprogrammed setting in most other PM devices (119). In general, most devices are turned into a so-called asynchronous mode by the magnetic effect. What exactly happens in this mode depends on the manufacturer and the device (119). At the end, one can even generalize, that finding a cardiac device at autopsy does not mean that the deceased required the device at the time of death.

## 9 Discussion

As indicated several times, the reconstruction of the causal chain leading to fatality can be complex and challenging. Based on the literature outlined so far, this task can best be accomplished conjoint by antemortem and postmortem experts. Such a bilateral approach has already been proclaimed for the assessment of sudden deaths (56, 122). Practical examples exist, for example, in Switzerland, where in 2015 the Swiss Society of Legal Medicine established a multidisciplinary working group together with clinicians and geneticists to jointly address sudden cardiac death (122). In the given context, beside clinical expertise, especially known psychological and psychiatric findings in the different cases has to be considered. In case these findings are ambiguous or even negative in terms of suicidality, psychological and psychiatric experts could be retrospectively involved to search for the underlying cause or overseen discrete hints. Such a retrospective psychiatric assessment of such cases could be helpful to improve suicide prevention in cardiac patients. However, such beneficial effects require the exact diagnosis of the cause of death. The current literature indicates, that in order to do so, a wide-ranging diagnostic approach is necessary. This can involve clinicians, law enforcement, legal medicine experts, and sometimes even the IcarDs manufacturer’s experts (123, 124). Prior case reports using such a broad and interdisciplinary assessment nicely outline that the presence of IcarDs never excludes a non-IcarD-associated cardiac cause of death (123). However, this shows that classifying death with and death due to an IcarD is just the first diagnostic step. In each scenario a further diagnostic segregation should be attempted, like outlined in the following.

### 9.1 Spectrum of death due to an implanted cardiac device

One scenario could be referred to as “accidental death due to IcarD.” This scenario would summarize all accidental malfunctions of a device leading to death, such as fateful malfunction of a LVAD. Another possible scenario would be “death due to IcarD associated with malpractice,” encompassing cases of individuals who died due to malpractice. It should be noted that in this scenario, the legal medicine expert is mainly employed to preserve evidence, and the final assessment is the responsibility of clinical experts. One might also encounter the “death due to IcarD associated with complications” scenario. This would subsume the deaths associated with a fateful fatal outcome due to complications, regardless of any short-or long-term complications about which the patients were previously informed. The two last scenarios could be termed “death due to IcarD employed for suicide” and “death due to IcarD employed for homicide.” These scenarios can only be resolved through a collaborative effort between law enforcement (e.g., presence or absence of a suicide note, presence or absence of signs of a fight), legal medicine (e.g., evidence of violation, evidence of self-harming behavior), and clinical experts (e.g., readout and interpretation of all encountered IcarDs, psychiatric medical history). Correct attribution can only be achieved by integrating all available information and may also require further subsequent analyzes, like toxicological analyzes to check for (self-)intoxication.

### 9.2 Spectrum of death with an implanted cardiac device

In this spectrum, mainly the area of “other than IcarD causes of death” is present, as pointed out by previous publications (123, 124). However, here the complex interplay of pathophysiology of the circulatory system, IcarD function, and the comorbidities must be considered. For example, autoptic findings must be questioned always to be able to distinguish between fatal mesenteric ischemia due to atherosclerosis and mesenteric ischemia due to cardiac low-output syndrome in the course of fatal device malfunction.

### 9.3 Intersection of death with and death due to an implanted cardiac device

Here, mainly one scenario has to be pointed out. This is the “clinical IcarD manipulation.” Its ambiguous character arises from the perennial ethical and legal debate about “turning off” IcarDs in end-of-life decisions. Accordingly, one could argue that the individual somehow died “accelerated” because the IcarD was turned off. But, one could also argue that the person died from an underlying cardiac disease even if the IcarD is still running. Thus, depending on one’s view and local legal and ethical circumstances, one could consider this scenario as either “death with IcarD” or “death due to IcarD.” In making this decision, it is important to keep in mind even clinical device manipulation (e.g., turning off an ICD to avoid inappropriate shocks in an individual’s final hours) does not have a uniform effect.

### 9.4 Legal and ethical aspects

Not only the determination of the cause of death, but also the correct classification of such is important. From a clinical perspective, the correct classification of the cause of death is considered to be helpful in determining therapeutic or preventive strategies (125). From a legal perspective, especially the manner of death has various consequences. These can vary between different countries and jurisdictions; among others, in Germany, the allowances for burial of the body (126) and potential exercise of legal jurisdiction. Ultimately, the correct determination of the cause of death and the manner of death (i.e., natural, violent or non-natural, or unclear) can have a far-reaching impact on health policy events, actions of investigating authorities, and the bereaved.

Legally, a primary criterion for a non-natural death is an “external” impact or influence (127) that establishes a chain of causation. In a medical setting, this definition is more complex and has been further defined and summarized in the *International Classification of Diseases.*

From a legal perspective, the intentional disconnection of a medical device shows *actus reus* and *mens rea* resulting in a criminal offense. In addition, it is an external intervention causing death and should thus technically be considered an unnatural death. However, if the patient did not communicate his/her intend, *actus reus* and *mens rea* cannot be established with certainty. An accidental interference thus raises the question what time frame constitutes a ‘sudden’ consequence of such manipulation and what time frame would be sufficient to establish a chain of causation. However, it must be noted that the determination of the cause and manner of death remains a scientific approach and not just a legal determination.

A particular type of IcarD manipulation is manipulation by a physician, such as deactivation during an end-of-life decision (128). This kind of manipulation is done, for example, to avoid inappropriate ICD shocks in an individual’s final hours. The deactivation of such devices is an ethical challenge (129) and should be assessed on an individual basis (130). An important factor in this situation is the patient’s right for self-determination (131, 132). In addition, there is a debate about the determination of “clinical deactivation” in end-of-life decisions. Some publications indicate that clinical deactivation of an IcarD could even be considered physician-assisted death (3, 4). The legality of physician-assisted death is a complex topic of ongoing discussion (133, 134) and highly dependent on the respective jurisdiction.

## 10 Summary and conclusion

**Figure 2:**
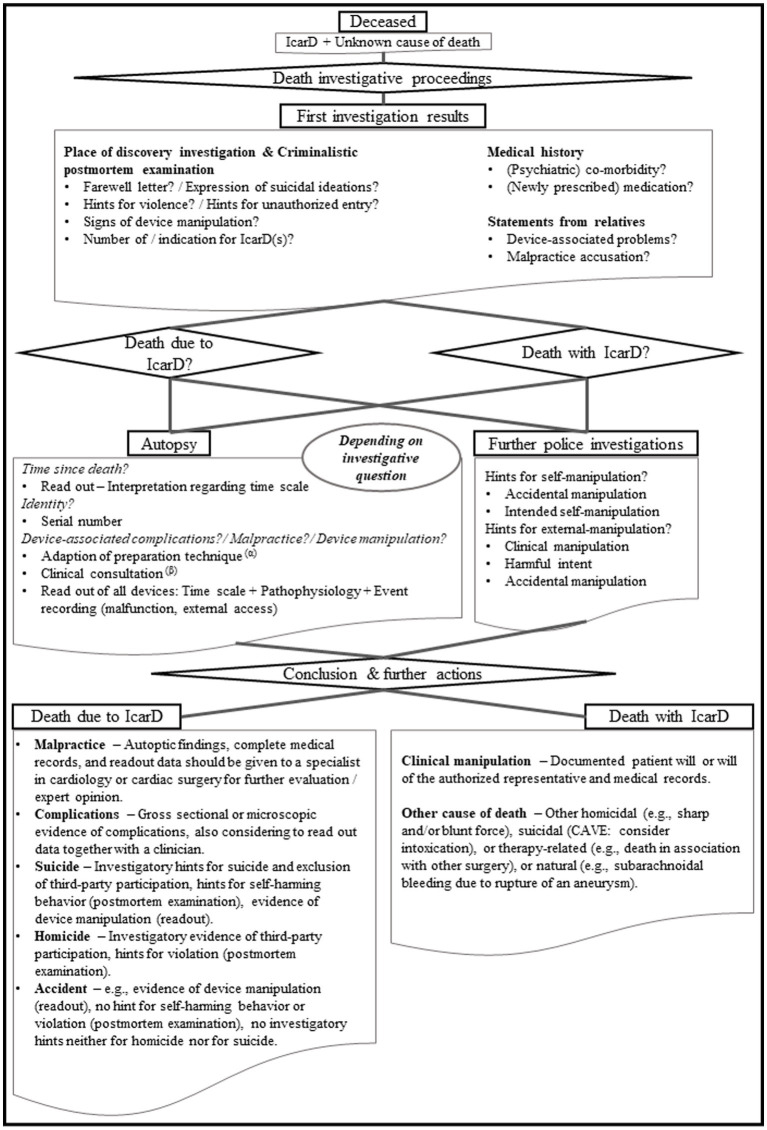
Assessment workflow for deaths in association with implanted cardiac devices. The selection of the different shapes was done aligned to what is known from entity-relationship models (i.e., the rectangles display an entity, ellipses represent attributes, and the diamond shape indicates a relationship). (α) The preparation technique should be adapted depending on the investigative questions and the encountered devices. So, for example if there is an CRT-D system and there is the question for lead-associated complications, the preparation should be done with special care for the venous system to be able to distinguish between antemortem findings and preparation-associated artifacts and to avoid dislocation of potentially adherent thrombi or vegetations. (β) Especially if there is a known complex pathophysiology underlying the IcarDs, one should consider a clinical consultation for conjoint interpretation of both autoptic findings together with the readout data and the known clinical history. The conjoint approach facilitates the integration of as many as possible information to a cause and by that manner of death. At this point it must be pointed out that the scenario “clinical manipulation” is somehow ambiguous. Depending on the underlying disease, the encountered IcarD or combination of IcarDs, turning off an IcarD can at least accelerate the occurrence of death (compare ethical and legal aspects of clinical IcarD manipulation in end-of-life decision). CRT-D, Cardiac Resynchronization Therapy – Defibrillator; IcarD, implanted cardiac device.

All in all, IcarDs can be considered potential “kill switches.” This can be of relevance for the casework in legal medicine due to the severe mental burden of IcarD carriers. To allow for both, suicide prevention and support of the investigating authorities, the exact cause of death has to be determined. Thereby, a conjoint approach of medical and forensic experts is necessary. In the medical field the antemortem and the postmortem field should be involved. Clinical experts, such as cardiologists or psychiatrics, can complement the autopsy and investigatory findings with their expertise and the retrospective analyzes of medical history and readout data. Regarding the readout, it should be attempted to collect data from all IcarDs. By that, manipulation or malfunction with potential relevance to the chain of causation of death can be detected. Therefore, these information can contribute to the clarification of cause and manner of death. The readout data may also establish a timeline of events and help in the overall evaluation of the case. A proposal of an assessment workflow is displayed in Figure 2. In such a conjoint approach the selection of the experts involved always has to consider potential conflicts of interest. Apart from this, a fundamental knowledge of the legal medicine expert regarding IcarDs is essential for an appropriate autopsy procedure and, by that, for the subsequent investigations (if necessary) by additional experts (Figure 2).

## Author contributions

JF: Conceptualization, Investigation, Visualization, Writing – original draft. SP: Investigation, Visualization, Writing – review & editing. KA: Validation, Writing – review & editing. KH: Validation, Writing – review & editing. SH: Investigation, Validation, Writing – review & editing. KB: Investigation, Writing – review & editing. MR: Investigation, Writing – review & editing. MK: Investigation, Writing – review & editing. CL: Writing – review & editing. PS: Writing – review & editing. MV: Writing – review & editing. FR: Conceptualization, Investigation, Writing – review & editing.
