# Supplementary material for: Forensic, legal, and clinical aspects of deaths associated with implanted cardiac devices
Source: Front Psychiatry. 2023 Nov 28;14:1278078. doi: 10.3389/fpsyt.2023.1278078 (PMC10713765; doi:10.3389/fpsyt.2023.1278078)
Supplement: Supplementary file 1 [file Data_Sheet_1.pdf]

Supplementary Material

Forensic, legal, and clinical aspects of deaths associated with implanted cardiac devices

Jan M. Federspiel[1,*], MD; Stefan Potente[1], MD; Karen B. Abeln[2], LLB, MD; Kai Hennemann[2]; Sara Heinbuch[3]; Katrin Burkhard[1], MD; Madita Richl[4]; Mattias Kettner[4], MD; Constantin Lux[4], MD; Peter Schmidt[1], MD; Marcel A. Verhoff[4], MD; Frank Ramsthaler[1], MD.

[1] Institute of Legal Medicine, Saarland University, Faculty of Medicine, Campus Homburg, Kirrberger Straße, Building 49.1, 66421 Homburg/Saar, Germany

[2] Department for Thoracic and Cardio-Vascular Surgery, Saarland University Medical Center, Building 57, Kirrberger Straße, 66421 Homburg (Saar).

[3] Department of Psychiatry, Clinic for Psychiatry, Psychotherapy and Psychosomatics, SHG-Kliniken Sonnenberg, Saarbrücken, Germany.

[4] Institute of Legal Medicine, University Hospital, Goethe-University of Frankfurt/Main, Frankfurt/Main, Germany.

*** Correspondence:**
Jan M Federspiel; Department of Legal Medicine, Saarland University, Kirrberger Straße, Building 49.1, 66421 Homburg/Saar; E-Mail: jmfederspiel@outlook.com.

For the supplement the Nature-citation style was chosen to distinguish the supplement from the main manuscript. The references used in the supplement are displayed in Supplement SG.

Supplement SA – Abbreviations used in manuscript and supplement in alphabetical order

Abbreviation	Meaning
BiVAD	Biventricular assist device
CCM	Cardiac contractility modulation
CPB	Cardio-pulmonary bypass
CRT(-D)	Cardiac resynchronization therapy (-Defibrillator)
ECHLS	Extracorporeal heart lung support
ECLS	Extracorporeal life support
ECMO	Extracorporeal membrane oxygenation
FDA	(United States) Food and Drug Administration
HCM	Hypertrophic cardiomyopathy
HF	Heart failure
HFpEF	Heart failure with preserved ejection fraction
HFrEF	Heart failure with reduced ejection fraction
IABP	Intraaortic balloon pump
IcarD	Implanted cardiac devices
ICD	Implantable Cardioverter / Defibrillator
INR	International Normalized Ratio
LVAD	Left ventricular assist device
LVOTO	Left ventricular outflow tract obstruction
MCS	Mechanical circulatory support
MRI	Magnetic resonance imaging
PM	Pacemaker

Abbreviation	Meaning
RVAD	Right ventricular assist device
SARS-CoV-2	Severe acute respiratory syndrome corona virus 2
VAD	Ventricular assist device
VA	Veno-arterial
VV	Veno-venous

Supplement SB – Clinical, pathophysiological, and pathological aspects of heart failure

Heart failure (HF) is a clinical syndrome defined by symptoms (e.g., dyspnea), signs (e.g., edema), and parameters such as intracardiac pressure (HF with preserved ejection fraction, HFpEF) and/or inadequate cardiac output (HF with reduced ejection fraction, HFrEF)[1]. HF is considered a "global burden"[2,3] due to its high prevalence (26 million people affected worldwide[2] accounting for approximately 1 to 2% of adults[1]) and incidence (among all age groups 3 out of 1000 and among adults 5 out of 1000 [1]). Overall different somehow "HF forms" (e.g., acute HF, chronic HF, HFrEF, or HFpEF [1]) are distinguished.

From the pathological perspective HF can be seen as a somehow common final pathway of a variety of diseases as among others infection/inflammation (e.g., myocarditis), heart valve disease (e.g., aortic valve stenosis), arterial hypertension, coronary heart disease[1] or heart rhythm disorders (e.g., tachycardiomyopathy[4]) can cause HF. Despite HF can have several etiologies, HF is far from being a uniform disease. For example, there is a difference in the therapy of acute and chronic HF as for HFrEF compared to HFpEF[1]. Another example would be the pathophysiology. Here in all instances but HF due to heart valve disease, the primary pathological change is an impaired ventricular function[5]. Talking about pathology and pathophysiology it has to be pointed out, that HF frequently is "whole hearted": although usually the focus is applied on the left ventricular impairment, this left ventricular dysfunction can be seen as the most common cause of changes in right ventricular geometry and function[6]. Also, important to highlight is, that HF is not also a cardiac disease but also presents with potential impairment of the whole body. So, for example, right ventricular dysfunction associated with congestion is among others associated with intestinal malabsorption, systemic inflammation, or congestive hepatopathy[6]. Speaking from the postmortem perspective it is at the same time understandable and surprising due to this pluralism, that the observed histomorphologic changes in congestive HF do not correlate with clinical symptoms[7].

Addressing its pathophysiology (e.g., neurohormonal activation in HFrEF[8]), the pharmacological therapy is seen as the "cornerstone" in HFrEF therapy[1]. Contrasting, the medical treatment options in HFpEF are very limited compared to HFrEF[9]. Besides pharmacological therapy, there is a field of devices applied in HF therapy for both HFrEF[1] and HFpEF[10].

Despite therapeutic options, mortality rates of up to 67% within 5 years after the initial diagnosis are reported for HF in general[1]. Overall mortality rates in HFpEF (27% in 2 years) are reported to be lower than in HFrEF (35% in 2 years)[11]. The comparison of HFrEF and HFpEF individuals shows HFrEF patients to be younger, more frequently male, have less frequently a history of arterial hypertension but present more frequently with coronary artery disease[11]. Due to the context of the later described cases, the present article focusses on HFrEF.

Supplement SC – Overview of cardiac devices

Not only is HF a complex pathology but also the field of carDs is brought and growing. Additionally, complicating are inconsistencies in the terminology of carDs, for example for the extracorporeal life support systems (ECLS): some authors describe ECLS as a "umbrella term including various modalities of temporary mechanical cardiopulmonary assistance of the failing heart and/or lungs"[12]. Opposing other authors describe ECLS as veno-arterial extracorporal membrane oxygenation (VA-ECMO)[13] only. Besides ECMO, also the term cardio-pulmonary bypass (CPB) can be found in the context of ECLS. Some authors state that ECLS circuits are somehow "evolved" CPB circuits with for example, smaller priming volume in ECLS and limited cooling possibility compared to CPB[14]. (The present manuscript employs "mechanical circulatory support" (MCS) as "umbrella term" and refers ECLS to be a veno-arterial ECMO. Due to the focus on postmortem aspects and the similarity of ECLS and CPB, the present manuscript does not further address CPB.) Another example for inconsistent terminology are the different sub-groups of ICDs. For example, some consider dual-chamber ICDs if there is a lead in the ventricle and in the atrium (e.g., reference [15]). Other authors call this a single-chamber device with additional lead for sensing (e.g., reference [16]).

However, the prevalence of HF increases[2]. Also the insertion rates of PMs as well as their prevalence rise[2]. At the same time, the number of individuals on VAD is increasing[17]. For ICDs there is a discussion whether the indication for implantation might be expanded[18] and how to handle "loss" of indication for primary preventive ICD over time, for example, due to changed recommendations by the clinical guidelines[19]. All in all, it seems likely that the legal medicine will face more frequently cases in association with HF and/or IcarDs. By that, it has to be noted, that different devices can be found "side by side" like exemplified in both later described.

To get started with IcarDs first a simplistic and general division is made. On one hand there are somehow "supportive" or "preventive" devices. On the other hand, there are devices in some way "replacing" cardiac function. First the field of the supportive and preventive devices is addressed.

"Supportive" and "preventive" cardiac devices

Implantable cardioverter / defibrillators

Talking about prevention, the first device to name are ICDs. They are primary or secondary preventively applied, for example in HFrEF patients[1,20] or in individuals with cardiac channelopathies such as Brugada syndrome spectrum [21,22]. They can also help to prevent fatal events in a somehow "side-by-side" manner with other devices such as LVADs[23] or can be part of a somehow "combination" device in a so-called cardiac resynchronization therapy – defibrillator (CRT-D) device (for example, Boston Scientific's DynagenTM X4 or InogenTM X4). Interestingly, while an ageing population is associated with an increased occurrence of IcarDs[3] and age is associated with an increased risk for sudden cardiac death (SCD), it has been reported that elderly carrying an ICD have an inferior survival compared to younger ICD carriers due to comorbidities[24]. Nevertheless, data from 5 landmark ICD trials found the profound arrhythmia to be the more important determinant for mortality compared to the ICD therapy itself[25].

ICDs it-self can be further sub-divided. One assigning factor for sub-division of ICDs is the position respectively the placement of the leads. There are subcutaneous, transvenously[26], and epicardially placed leads[27,28]. The subcutaneous devices (e.g., Boston Scientific's EmblemTM) has been developed to avoid complications associated with transvenous leads such as dislocation and transvenous lead placement such as bleeding[26]. Regarding the number and position of transvenously placed leads, ICDs can be classified as single-chamber, dual-chamber, or triple-chamber ICD[29]. The triple chamber ICD can also be referred to as biventricular ICD[29]. Which device is used depends from the respective case (e.g., is there a pacing indication?[15]). After the decision is made how many chambers are addressed, also the decision which device will be implanted has to be made. So, for example, there is a discussion whether to apply a conventional ICD system or to use a CRT-D system without left ventricular lead as long as no CRT is needed[30].

In subcutaneous ICDs the lead is usually placed parasternal[31]. In transvenous systems the leads are usually placed in the right atrium and the right ventricle (compare e.g., references [14,16,32]) and for biventricular systems also in the coronary sinus (compare e.g., references [29,33]). But there are also distinct populations that require alternative lead placements. One example would be individuals after tricuspid valve surgery (here a transition of the tricuspid valve is contraindicated and leads can be placed in the middle cardiac vein or epicardially[27]). Another example are individuals with severely congenitally malformed hearts (for example tricuspid atresia), here epicaridal lead placement can be applied too[28]. It has also to be pointed out that there are plenty of variations how PM and ICD can be combined. So for, example the combination of an subcutaneous ICD with an bipolar and epicardial left ventricular PM has been reported[34].

Several aspects of the ICD can be summarized in the so-called "Defibrillator-Code"[35]. This nomenclature system provides in a precise and concise way various important information on a device (e.g., shock chamber, presence/absence of a antitachycardia pacing site, the mode of tachycardia detection, or the absence/presence of a antibradycardia pacing site)[35].

However, coming to the PM next, it has to be pointed out that the borderline between PMs and ICDs is blurred. One example is the potential clinical use of CRT-D devices without left lead instead of classical ICD[30]. Another point where the borderline is softened, is the capability of some ICDs to pace[27], for example if they detect bradycardia[32]. This blurred borderline also applies for the complications. For example, both ICD and PM with transmembranous leads are associated with neointimal fibrotic lead encapsulation[36] leading to common strategies for lead management[33]. For both of these most frequently encountered IcarDs (i.e., PM[37] and ICD[38]) the most frequent complications around the implantation are bleeding, infection, or lead displacement. Thereby, evolving technology can help to reduce complications, as for example the leadless PM has been demonstrated to be associated with a reduced risk of device-related infections[39].

Cardiac pacemakers

Like for ICD, also for PM further sub-division is possible. One potential assigning factor is the positioning of the leads. There are epicardial pacemakers (for example, applied in neonates with congenital block of the atrioventricular conduction[40] or for temporary pacing after cardiac surgery[41], transvenous PM (for example, used for temporary pacing in an emergency setting[42] as well as for long-term pacing[43]) and currently quite new so-called “leadless pacemakers”[44]. Another potential assigning factor is by the number of sensed or stimulated chambers, i.e., single-chamber[45], dual chamber[45], or triple chamber PM[46]. Moreover, also the pacing mode can be seen as grouping factor for PM, i.e., atrial sensing and/or pacing with a single lead, ventricular sensing and/or pacing with a single lead, and so on[47].

PMs can be both “preventive” and “supportive”. One example for preventive PM are 2-chamber devices that can prevent potentially lethal bradycardia[1]. CRT-systems (3-chamber-PM: right atrium, right ventricle, left ventricle via coronary sinus[48]) can be supportively applied in HF patients[1] as HF has been associated with ventricular desynchronization[49]. The CRT can improve the myocardial functioning[50]. Besides the CRT there is also the so-called His-bundle pacing as particular sub-type of pacing[47]. His-bundle pacing is referred to as “alternative method”[47] and can for example be applied in a subset of patients with high-grade block of the atrioventricular conduction and short QRS-complexes at the same time[47]. Also in hypertrophic cardiomyopathy (HCM) with left ventricular outflow tract obstruction (LVOTO), PM can be applied somehow “supportively” with the intent to reduce LVOTO[51].

Like for ICDs there is also a summarizing code available for pacemaker[52,53]. In five positions this code describes which chamber is paced, which chamber is sensed, how the device responds to sensing, presence/absence of rate modulation, and last information regarding multisite pacing[53].

“Replacing” cardiac devices: mechanical circulatory support

Following the simplified classification of IcarDs, the cluster of “preventive” and “supportive” has been addressed so far. Now the field of “replacing” devices will be highlighted. These devices are used for so-called mechanical circulatory support (MCS). In this group of devices, the focus on total artificial hearts is reported to be shifted from total artificial hearts towards VADs as device failure in total artificial hearts can hardly be survived[54]. Nevertheless, total artificial hearts are applied in particular settings and/or groups. One example would be the Syncardia Total Artificial Heart in a pediatric population[55]. Indication for VAD-therapy can be a lack of adequate symptom relieve with conservative therapy (so-called advanced HF)[1]. After the indication for a VAD was seen, a critical clinical evaluation for suitability of the patient for the VAD has to be carried out[1].

Again, the MCS family of therapies and devices can be further sub-divided by several aspects. One is the differentiation by the supported cardiac chamber, i.e., left ventricular, right ventricular or biventricular assist devices (respectively LVAD, RVAD; BiVAD)[54]. As also systems such as CPB or ECMO can be assigned to the MCS family, a differentiation regarding the supported organ or organ system can be made: there are systems such as the Impella that are designed to support the heart[56] (“heart only”) while other systems like the ECMO can support respiratory and circulatory system[57]

(heart and lung support) and other systems like the so-called Homburg lung mainly support the respiratory system[58] (lung support). Considering the anatomy, they can also be divided by the position of the pump in relation to the body, in terms intracorporeal pumps such as the INCOR device[59] or "paracorporeal" devices such as the EXCOR device[60]. From the technical point of view MCS devices can be sub-divided into different classes by the flow in several aspects. One is the flow the device generates, i.e., a device with either pulsatile or continuous flow[54]. Another aspect can be how the flow is generated, so centrifugal and axial flow blood pumps can be distinguished[61]. Considering the timespan of the MCS for example short-[1,62], mid-[63], and long-term MCS therapy[1,62] can be distinguished. Thereby, these different forms can merge as, for example short-term MCS can be used to bridge to a long-term MCS[62]. By doing so, different devices are applied for the different time scales. Among others ECMOs[64,65]respectively ECLS[66], the so-called Impella®[65] or combinations of aforementioned devices (so-called ECMELLA)[67] can be used for shorter time scales. But, the Impella® for example, can also be used for a mid-term support to a long-term therapy[63]. For the long-term therapy devices like the EXCOR[60] or the HeartMate3[68] can be applied. Not only regarding the approached time scale but also regarding the approached final "goal" the MCS therapy can be sub-divided (e.g., bridge to destination or transplant[55,69], bridge to long-term therapy[63] or bridge to recovery[70]). Besides the so far mentioned devices also the intra-aortic balloon-pump (IABP) is available and usually applied for short-term MCS[71]. Some reports on "ambulatory" IABP to bridge to heart transplant are available[72]. This system uses counterpulsation to improve coronary blood flow and increases left ventricular stroke volume by lowering the left ventricular afterload[71].

Other devices

Besides the aforementioned comparably frequent applied IcarDs, also other devices are available. One example is the cardiac contractility modulation (CCM) therapy as illustration of the continuous development of new devices and approaches increasing the panel of available IcarDs. CCM employs non-excitatory electrical signals to cause positive inotropic effects[73]. So far, CCM is currently not yet recommended for the clinical routine according to recent clinical guidelines[1] and therefore not further addressed in the present manuscript.

Implantable loop recorders can be implanted to detect rare arrhythmias[21] and can by that help to detect atrial fibrillation[74], and support clarification of the cause of syncope[75] or stroke[76]. Compared to the high prevalence of ICDs[3] or pacemakers[2] these devices are rare[75]. Additionally, they are not addressed by the current clinical HF guideline and for these reasons not further addressed in the present manuscript.

Summary

Table C.1 provides a brief summary of addressed IcarDs and the different sub-groups. Table C.2 gives an overview how the different IcarD groups are usually implanted.

Table C.1 – Summary of implantable cardiac devices and their sub-groups

Implantable cardiac devices (IcarDs)

<table>
<tr><th colspan="2">„Preventive“ & „supportive“ IcarDs</th><th colspan="2">„Replacing“ IcarDs (= MCS)</th><th>Other IcarDs</th></tr>
<tr><td>ICD</td><td>PM</td><td rowspan="3">Time scale of therapy:
Short-term
Mid-term
Long-term

Goal of MCS:
Bridge to transplant
Bridge to destination
Bridge to recovery
Bridge short-term MCS to long-term MCS

Flow characteristics:
Continuous flow
Pulsatile flow

Flow generation:
Axial flow pump
Centrifugal flow pump</td><td rowspan="3">Position of the pump:
Extracorporeal
Intracorporeal

Supported organ:
Heart support
Heart and lung support
Lung support

Supported cardiac chamber:
Left ventricle
Right ventricle
Both ventricles

Extent of cardiac support:
Ventricular assist device
Total artificial heart

Mechanism of action:
Antegrade flow
Counterpulsation</td><td rowspan="3">Cardiac contractility modulation (CCM)

Implantable loop recorder</td></tr>
<tr><td>Chambers covered:
Singe chamber
Dual chamber
Triple chamber

Lead placement:
Subcutanous
Transvenous
Epicardial</td><td>Time scale:
Temporary
Permanent

Chambers covered:
Single chamber
Dual chamber
Triple chamber

Lead placement:
Epicardial
Transvenous
„Leadless“

Others:
His-Pacing
HCM / LVOTO</td></tr>
<tr><td colspan="2">Cross-over systems
- CRT(-D) Systems with and without left ventricular lead
- ICD systems with the ability to pace</td></tr>
</table>

The table summarizes the different IcarDs based on the simplified “classification” described in the text. Thereby, also the different subgroups are displayed. Abbreviations: CCM – cardiac contractility modulation; CRT-(D) – cardiac resynchronization therapy-(defibrillator); HCM – hypertrophic cardiomyopathy; IcarD – implantable cardiac device; ICD – implantable cardioverter / defibrillator; LVOTO – left ventricular outflow tract obstruction. MCS – mechanical circulartoy support; PM – pacemaker;

Table C.2 – Location of IcarDs

PM / CRT-D	***Aggregate:***<br>• Adults – Usually in a pectoral pocket<br>• Infants / smaller children – Abdomen<br>***Leads:***<br>• Mostly: Transvenous to right atrium / right ventricle / coronary sinus<br>• Children / post cardiac surgery: Epicardial<br>***Special Case – Leadless pacemaker***<br>• Electrode and aggregate are place intracavitarily in the right ventricle
ICD	***Aggregate:***<br>Usually in a pectoral pocket<br>**Leads:**<br>Usually transvenously to right atrium / right ventricle<br>Subcutaneous
CPB / ECMO / ECLS / Homburg Lung	• Depending on case / anatomy / underlying disease vascular grafts can be used for allow access of a cannula.<br>• Depending on the chosen approach different cannulas and cannulation sides can be used. For example, to achieve good venous drainage double venous cannulation via jugular vein and femoral vein can be applied.<br>• Most commonly application via the femoral vessels +/- jugular vein. Open chest approach compared to the periphery cannulation rather scarce.
Impella ®	• Depending on case / anatomy / underlying disease vascular grafts can be used for allow access of the device. Lies usually placed across the semilunar valve, allowing for aspiration of blood within the supported ventricular chamber and ejection of blood into the connected downstream large artery.<br>• Several implantation sites are possible. For example access to the LV is also possible via the right subclavian artery. But the Impella can also be implanted via the femoral artery or via the aorta directly. So, one has to consider the possibility of surgical and non-surgical Impella implantation
Long-term VAD	• Depending of the supported ventricle(s). Usually one cannula allowing for “unload” of the heart (e.g., via the apex cordis for an LVAD) and one cannula allowing for return of the blood after the supported chamber is bypassed (e.g., aorta).<br>• The pump itself can be located within the body or outside the body. Irrespective, where the pump is placed there is always a structure passing through the skin (blood flow components in case of extracorporeal pump; drive line in case of intracorporeal pump).
IABP	• Usually applied via the femoral artery and placed in the descending aorta avoiding occlusion of the mesenteric arteries.

The table summarizes general and common features regarding the positioning and implantation of different IcarDs. This summary makes no complain to be complete, as from case to case, especially dependent from the particular anatomy and underlying pathophysiology, there can be a massive variation of how and where the devices are implanted. This variety underpins the need for comprehensive investigations by the law enforcement authorities, if there is the question whether a person died with an IcarD or due to an IcarD to facilitate proper planning of the autopsy to facilitate best possible information yield.

Supplement SD – Case descriptions

Case 1

A 67-year-old male (body weight 96 kg, body height 181 cm) was found dead in his apartment in the afternoon in an oblique sitting, to lying position on a sofa. The emergency physician declared him dead with no further resuscitation attempts. Next to the body, the drive line of an LVAD (Abbott® HeartMate3) which had been implanted 4 months prior to death, was found disconnected. There were no indicators for a break-in, a fight, or any other foul play. No suicide note was present either. Regarding the medical history, the individual had received stenting of the left anterior descending coronary artery, undergone implantation of a combined ICD-pacemaker system (Medtronic® Amplia MRI Quad SureScan CRT-D), suffered from ischemic cardiomyopathy and myocardial infarction in the past, and received surgical aortic valve replacement with a stented biological prosthesis.

The postmortem examination yielded small, fine scars at both wrists, a potential hint for suicidal or self-harming behavior (**figure D.1.A**), and a limited number of petechial hemorrhages especially of the left conjunctiva (**figure D.1.B**) and the back of the right foot. No indicators of strangulation (e.g., strangulation mark), blunt force injury (e.g., hematoma, swelling) or any other violent action (e.g., fresh incision wounds) were present. Both the LVAD and CRT/ICD systems showed no autoptic signs of device-associated complications such as thrombotic material in the outflow or displacement of the leads. The heart (**figure D.2A**) with the LVAD (**figure D.2.B**, and **D.2.C**) is displayed in **figure D.2**. The heart was massively enlarged compared to the person's fist (heart weight 847 g, **figure D.2.A**) and no physical damage was visible on the LVAD (**figure D.2.B**, and **D.2.C**) or the ICD-pacemaker device (additional LVAD equipment is displayed in **figure D.3**).

The read out of the LVAD was performed with help of the local LVAD-commissioner and help of the local team of perfusionists. The read out showed that the disconnection occurred roughly 19 hours prior to finding the body in the early evening. The read out of the ICD-pacemaker documented ventricular arrhythmias with shock 20 minutes and 38 minutes after disconnection. As a preliminary result, death was ruled non-natural, consistent with suicide.

Figure D.1 – Findings upon external examination of the corpse in case 1

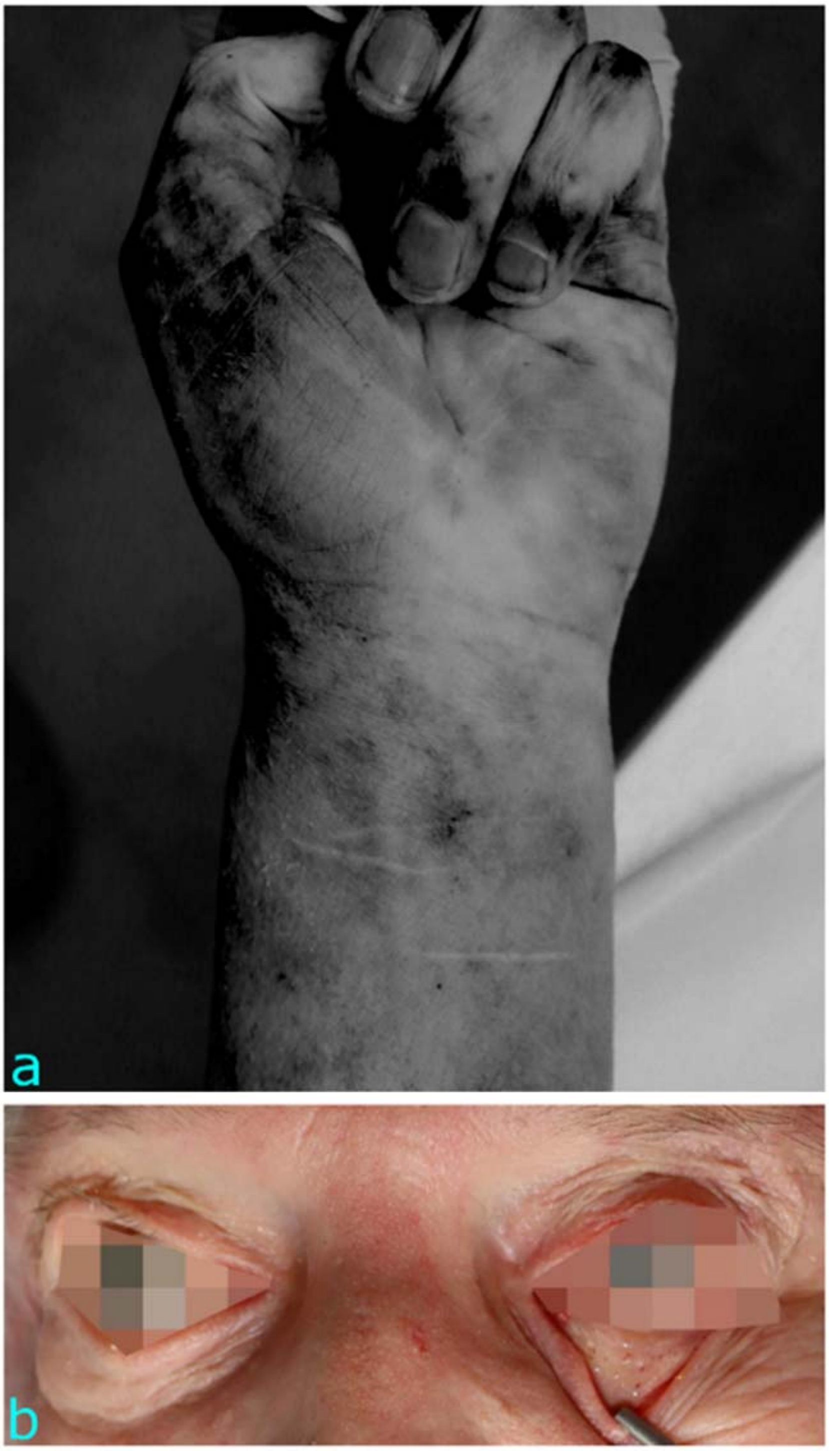

(a) Small scars on the left wrist. (b) Petechiae in the conjunctiva of the left eyelid.

Figure D.2 – Heart with LVAD of case 1

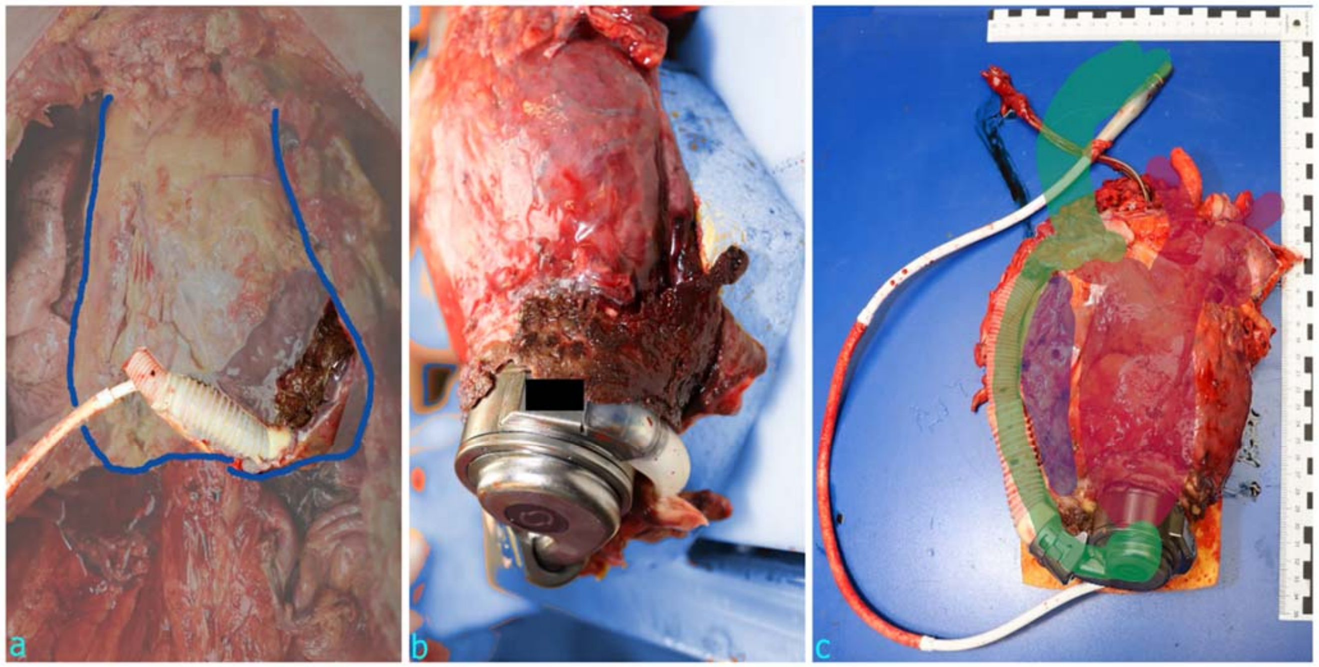

(a) Heart with LVAD in situ after removal of the sternum and the anterior section of the rips. (b) Detail picture of the pump housing of the LVAD. (c) Explanted heart. Marked cardiac hypertrophy (heart weight without device 647 g). The white line at the bottom of the pictures is the drive line. The green overlay displays the LVAD with the tunnel from the ventricle to the aorta. Above the heart the green overlay symbolizes the position of the ascending aorta in situ. The blue overlay resembles the right ventricle. The red overlay resembles the enlarged pulmonary veins entering the left atrium, the left atrium, and the left ventricle.

Figure D.3 – Adjuncts to the implanted LVAD and other medical devices in case 1

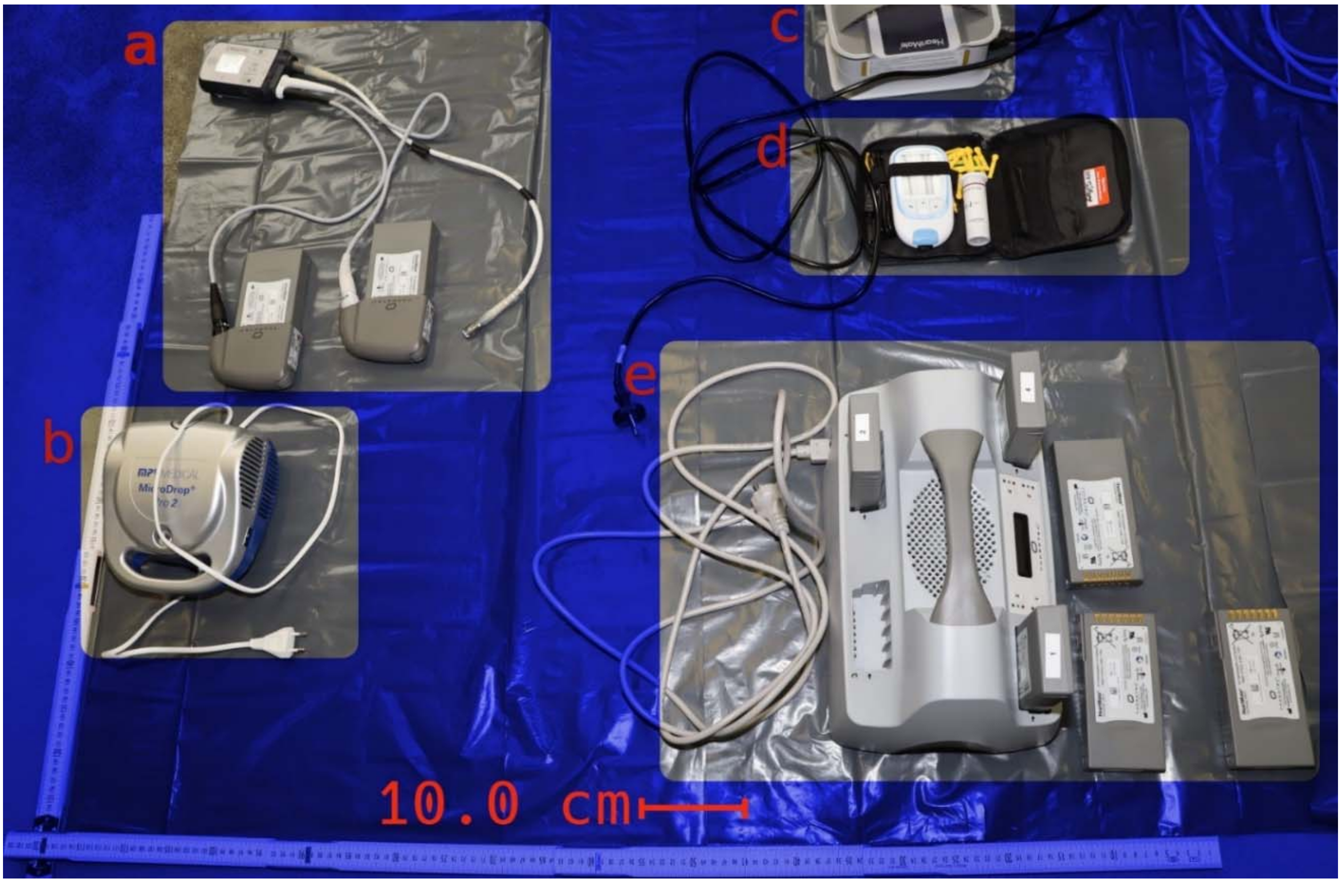

(a) Two batteries with so called clips, connected to the system controller via the modular drive line. (b) inhalation device used by the decedent. (c) Mobile power unit. (d) CoaguCheck® device to control anticoagulation via measuring the International Normalized Ratio (INR). (e) Batteries with the Battery Charger

Case 2

A 56-year-old male was found dead by nursing staff lying in the bed of his apartment. The emergency physician declared him dead and attested an unexplained death. During postmortem examination a LVAD (Manufacturer details unknown) and a long-term oxygen therapy device were found. Both rechargeable batteries of the LVAD were disconnected and the device was connected to a power cable without connection to an electrical outlet. One hand of the decedent was lying on the LVAD. The oxygen therapy was applied correctly. The apartment was closed correctly and exhibited no signs of unauthorized violent entry or a fight. A farewell letter was not found. The man had not voiced specific suicidal intentions towards his doctor, but he had told her, that he was unhappy about his whole situation and that he would end it one day.

Postmortem examination did not give hints for foreign violation. During autopsy an ICD (Manufacturer details unknown) besides the LVAD was found. The autopsy showed that the both devices were applied correctly. No signs of device-associated problems (e.g., displacement) were found. The main autoptic findings included cardiac hypertrophy, coronary heart disease, especially of the left anterior descending, signs of chronic congestive heart failure, and signs of a chronic obstructive pulmonary disease. There were no signs of violence or a fight before death. LVAD malfunction - intentional or unintentional - was defined as the cause of death.

The read out of the devices showed that two disconnections of the LVAD occurred a few hours before death. The first one was at 02:17 am, the second one was at 02:32 am as the final documented event. It was considered that after the first disconnection the alarm displaying a disconnection was set off before the second disconnection. The ICD was not read out. In summary, the findings suggested suicide as the most likely event.

Results of both reported cases

In the first case, the time and cause of death (i.e., ventricular arrhythmia) could be determined more accurately because all ICarDs were read. This emphasizes how important data collection from all IcarDs can be for reconstructing events leading to death and determining cause and mode of death in the presence of such devices.

Supplement SE – PubMed outputs of systematic literature searches

(I) **Literature search on a potential association between CCM and suicide**

Data base: PubMed

Last data base query: 4th May 2023, 03:10 pm.

Search algorithm: (suicide) AND (cardiac contractility modulation)

No results were found.

(II) **Literature search on a potential association between implantable loop recorders and suicide**

Data base: PubMed

Last data base query: 4th May 2023, 03:15 pm.

Search algorithm: (suicide) AND (implantable loop recorder)

1: Van Donge N, Schvey NA, Roberts TA, Klein DA. Transgender Dependent Adolescents in the U.S. Military Health Care System: Demographics, Treatments Sought, and Health Care Service Utilization. Mil Med. 2019 May 1;184(5-6):e447-e454. doi: 10.1093/milmed/usy264. PMID: 30325452.

(III) **Literature search on a potential association between CRT and suicide**

Data base: PubMed

Last data base query: 04.05.2023 ~15:00

Search algorithm: (suicide) AND (cardiac resynchronization therapy)

1: Daeschler M, Verdino RJ, Kirkpatrick JN. The ethics of unilateral implantable cardioverter defibrillators and cardiac resynchronization therapy with defibrillator deactivation: patient perspectives. Europace. 2017 Aug 1;19(8):1343-1348. doi: 10.1093/europace/euw227. PMID: 27915265.

2: Cleland JG, Velavan P, Nasir M. Fighting against sudden death: a single or multidisciplinary approach. J Interv Card Electrophysiol. 2006 Dec;17(3):205-10. doi: 10.1007/s10840-006-9077-6. Epub 2007 Apr 6. PMID: 17415628.t

---

(IV) **Literature search on a potential association between MCS and suicide**

Data base: PubMed

Last data base query: 04.05.2023 ~12:20

Search algorithm: (suicide) AND (mechanical circulatory support)

1: Mueller PS. Ethical and Legal Concerns Associated With Withdrawing Mechanical Circulatory Support: A U.S. Perspective. Front Cardiovasc Med. 2022 Jul 26;9:897955. doi: 10.3389/fcvm.2022.897955. PMID: 35958394; PMCID: PMC9360408.

2: Pak ES, Jones CA, Mather PJ. Ethical Challenges in Care of Patients on Mechanical Circulatory Support at End-of-Life. Curr Heart Fail Rep. 2020 Aug;17(4):153-160. doi: 10.1007/s11897-020-00460-4. PMID: 32488502.

3: Rady MY, Verheijde JL. Ethical challenges with deactivation of durable mechanical circulatory support at the end of life: left ventricular assist devices and total artificial hearts. J Intensive Care Med. 2014 Jan- Feb;29(1):3-12. doi: 10.1177/0885066611432415. Epub 2012 Mar 6. PMID: 22398630.

4: Mazzola MA, Russell JA. Neurology ethics at the end of life. Handb Clin Neurol. 2023;191:235-257. doi: 10.1016/B978-0-12-824535-4.00012-4. PMID: 36599511.

5: Baum C, Bohnen S, Sill B, Philipp S, Damerow H, Kluge S, Reichenspurner H, Blankenberg S, Söffker G, Barten MJ, Sydow K. Prolonged resuscitation and cardiogenic shock after intoxication with European yew (Taxus baccata): Complete recovery after intermittent mechanical circulatory support. Int J Cardiol. 2015 Feb 15;181:176-8. doi: 10.1016/j.ijcard.2014.11.221. Epub 2014 Dec 13. PMID: 25528306.

6: Mohite PN, Patil NP, Sabashnikov A, Zych B, García Sáez D, Popov AF, De Robertis F, Bahrami T, Amrani M, Reed A, Carby M, Simon AR. "Hanging donors": are we still skeptical about the lungs? Transplant Proc. 2015 Mar;47(2):261-6. doi: 10.1016/j.transproceed.2014.12.026. PMID: 25769558.

7: Nakagawa S, Uriel N, Prager KM. Should It Be Called "Suicide" or "Withdrawal of LVAD Support"? J Pain Symptom Manage. 2020 Nov;60(5):e1-e3. doi: 10.1016/j.jpainsymman.2020.08.021. Epub 2020 Aug 22. PMID: 32835832.

8: Rady MY, Verheijde JL. Continuous deep sedation until death: palliation or physician-assisted death? Am J Hosp Palliat Care. 2010 May;27(3):205-14. doi: 10.1177/1049909109348868. Epub 2009 Dec 14. PMID: 20008825.

9: Bologa C, Lionte C, Popescu A, Sorodoc V, Sorodoc L. First Case of Acute Poisoning with Amiodarone and Flecainide in Attempted Suicide Successfully Managed with Lipid Emulsion Therapy in the Emergency Department: Case Report and Literature Review. Healthcare (Basel). 2021 Jun 4;9(6):671. doi: 10.3390/healthcare9060671. PMID: 34199756; PMCID: PMC8226996.

10: Gariboldi V, Grisoli D, Tarmiz A, Jaussaud N, Chalvignac V, Kerbaul F, Collart F. Mobile extracorporeal membrane oxygenation unit expands cardiac assist surgical programs. Ann Thorac Surg. 2010 Nov;90(5):1548-52. doi: 10.1016/j.athoracsur.2010.06.091. PMID: 20971261.

11: Ran Q, Xiang Y, Liu Y, Xiang L, Li F, Deng X, Xiao Y, Chen L, Chen L, Li Z. Eryptosis Indices as a Novel Predictive Parameter for Biocompatibility of Fe3O4 Magnetic Nanoparticles on Erythrocytes. Sci Rep. 2015 Nov 5;5:16209. doi: 10.1038/srep16209. PMID: 26537855; PMCID: PMC4633654.

12: Magdalan J, Antończyk A. Trzy przypadki samobójczego zatrucia Morbitalem-preparatem do eutanazji małych zwierzat [Three cases of suicidal morbital intoxication--preparation for euthanasia of small animals]. Pol Arch Med Wewn. 2006 Feb;115(2):139-43. Polish. PMID: 17274470.

13: Ananiadou O, Schmack B, Zych B, Sabashnikov A, Garcia-Saez D, Mohite P, Weymann A, Mansur A, Zeriouh M, Marczin N, De Robertis F, Simon AR, Popov AF. Suicidal hanging donors for lung transplantation: Is this chapter still closed? Midterm experience from a single center in United Kingdom. Medicine (Baltimore).

2018 Apr;97(14):e0064. doi: 10.1097/MD.0000000000010064. PMID: 29620623; PMCID: PMC5902298.

14: Sein Anand J, Barwina M, Zajac M, Kaletha K. Suicidal intoxication with potassium chlorate successfully treated with renal replacement therapy and extracorporeal liver support. Przegl Lek. 2012;69(8):585-6. PMID: 23243935.

15: Vos EM, Hens JJ, Lau HS, Boon ES, Bartelink AK. Intoxicatie met organofosforverbindingen [Poisoning with organophosphate compounds]. Ned Tijdschr Geneeskd. 2002 Jan 5;146(1):34-7. Dutch. PMID: 11802337.

16: Stachoń K, Dabek J, Jakubowski D, Rychlik W. Implikacje kliniczne u chorych po spozyciu nadmiernej dawki leków blokujacych kanały wapniowe—obserwacje własne [The clinical implications in patients after calcium channel blockers overdose--own observations]. Pol Merkur Lekarski. 2011 Sep;31(183):145-9. Polish. PMID: 21991842.

17: Siddaiah L, Adhyapak S, Jaydev S, Shetty G, Varghese K, Patil C, Iyengar S. Intra-aortic balloon pump in toxic myocarditis due to aluminum phosphide poisoning. J Med Toxicol. 2009 Jun;5(2):80-3. doi: 10.1007/BF03161093. PMID: 19415593; PMCID: PMC3550326.

18: Incagnoli P, Bourgeois B, Teboul A, Laborie JM. Survie sans séquelles d'un arrêt cardiaque avec hypothermie sévère à 22 degrés C: importance de la stratégie de prise en charge préhospitalière [Resuscitation from accidental

hypothermia of 22 degrees C with circulatory arrest: importance of prehospital management]. Ann Fr Anesth Reanim. 2006 May;25(5):535-8. French. doi: 10.1016/j.annfar.2006.01.011. Epub 2006 Mar 3. PMID: 16516435.

(V) **Literature search on a potential association between VADs and suicide**

Data base: PubMed

Last data base query: 04.05.2023 ~12:25

Search algorithm: (suicide) AND (ventricular assist device)

1: Sladen RN, Shulman MA, Javaid A, Hodgson C, Myles PS, Mcgiffin D, Nakagawa S, Amlani AM, Hupf J, Takeda K, Naka Y, Takayama H, Bergin P, Buckland MR, Yozefpolskaya M, Colombo PC. Postdischarge Functional Capacity, Health-Related Quality of Life, Depression, Anxiety, and Post-traumatic Stress Disorder in Patients Receiving a Long-term Left Ventricular Assist Device. J Card Fail. 2022 Jan;28(1):83-92. doi: 10.1016/j.cardfail.2021.07.019. Epub 2021 Aug 20. PMID: 34425221.

2: Tigges-Limmer K, Schönbrodt M, Roefe D, Arusoglu L, Morshuis M, Gummert JF. Suicide after ventricular assist device implantation. J Heart Lung Transplant. 2010 Jun;29(6):692-4. doi: 10.1016/j.healun.2009.12.005. Epub 2010 Mar 5. PMID: 20207168.

3: Chernyak Y, Teh L, Henderson DR, Patel A. Practice Issues for Evaluation and Management of the Suicidal Left Ventricular Assist Device Patient. Prog Transplant. 2020 Mar;30(1):63-66. doi: 10.1177/1526924819893300. Epub 2019 Dec 26. PMID: 31876252.

4: Choi JJ, Peters CJ, Nickels MW. Arranging Inpatient Psychiatric Treatment for a Patient with a Left Ventricular Assist Device. Prog Transplant. 2022 Sep;32(3):248-251. doi: 10.1177/15269248221107033. Epub 2022 Jun 10. PMID: 35686352.

5: Balliet WE, Madan A, Craig ML, Serber ER, Borckardt JJ, Pelic C, Barth K, Hale A, van Bakel AB, Peura JL. A Ventricular Assist Device Recipient and Suicidality: Multidisciplinary Collaboration With a Psychiatrically Distressed Patient. J Cardiovasc Nurs. 2017 Mar/Apr;32(2):135-139. doi: 10.1097/JCN.0000000000000293. PMID: 26422637; PMCID: PMC4811747.

6: Jiménez-Blanco Bravo M, Zamorano Gómez JL, Del Prado Díaz S, Alonso Salinas GL. A suicide attempt on a left ventricular assist device patient during COVID-19 pandemic: can we only blame the virus? A case report. Eur Heart J Case Rep. 2021 May 12;5(5):ytab144. doi: 10.1093/ehjcr/ytab144. PMID: 34013162; PMCID: PMC8117427.

7: McIlvennan CK, Wordingham SE, Allen LA, Matlock DD, Jones J, Dunlay SM, Swetz KM. Deactivation of Left Ventricular Assist Devices: Differing Perspectives of Cardiology and Hospice/Palliative Medicine Clinicians. J Card Fail. 2017 Sep;23(9):708-712. doi: 10.1016/j.cardfail.2016.12.001. Epub 2016 Dec 5. PMID: 27932271.

8: Rady MY, Verheijde JL. Ethical challenges with deactivation of durable mechanical circulatory support at the end of life: left ventricular assist devices and total artificial hearts. J Intensive Care Med. 2014 Jan- Feb;29(1):3-12. doi: 10.1177/0885066611432415. Epub 2012 Mar 6. PMID: 22398630.

9: Cochrane C. Having Heart. Creat Nurs. 2015;21(4):211-2. doi: 10.1891/1078-4535.21.4.211. PMID: 26731919.

10: Mueller PS. Ethical and Legal Concerns Associated With Withdrawing Mechanical Circulatory Support: A U.S. Perspective. Front Cardiovasc Med. 2022 Jul 26;9:897955. doi: 10.3389/fcvm.2022.897955. PMID: 35958394; PMCID: PMC9360408.

11: Pak ES, Jones CA, Mather PJ. Ethical Challenges in Care of Patients on Mechanical Circulatory Support at End-of-Life. Curr Heart Fail Rep. 2020 Aug;17(4):153-160. doi: 10.1007/s11897-020-00460-4. PMID: 32488502.

12: Mueller PS, Swetz KM, Freeman MR, Carter KA, Crowley ME, Severson CJ, Park SJ, Sulmasy DP. Ethical analysis of withdrawing ventricular assist device support. Mayo Clin Proc. 2010 Sep;85(9):791-7. doi: 10.4065/mcp.2010.0113. Epub 2010 Jun 28. PMID: 20584919; PMCID: PMC2931614.

13: Groetzner J, Reichart B, Roemer U, Reichel S, Kozlik-Feldmann R, Tiete A, Sachweh J, Netz H, Daebritz S. Cardiac transplantation in pediatric patients: fifteen-year experience of a single center. Ann Thorac Surg. 2005 Jan;79(1):53-60; discussion 61. doi: 10.1016/j.athoracsur.2003.12.075. PMID: 15620914.

14: Lindemann H. When stories go wrong. Hastings Cent Rep. 2014 Jan-Feb;44(1Suppl):S28-31. doi: 10.1002/hast.266. PMID: 24408704.

15: Skouri H, Shurrab M, Haj-Yahia S. Saved Lives at Risk in the Middle East. ASAIO J. 2016 May-Jun;62(3):359-60. doi: 10.1097/MAT.0000000000000334. PMID: 26735560.

16: Capin I, Capone CA, Taylor MD. Acute on Chronic Heart Failure Secondary to Left Ventricular Noncompaction. Case Rep Pediatr. 2020 Oct 26;2020:6369806. doi: 10.1155/2020/6369806. PMID: 33163242; PMCID: PMC7605943.

17: Charton M, Flécher E, Leclercq C, Delmas C, Dambrin C, Goeminne C, Vincentelli A, Michel M, Lehelias L, Verdonk C, Para M, Pozzi M, Obadia JF, Boignard A, Chavanon O, Barandon L, Nubret K, Kindo M, Minh TH, Gaudard P, Pelcé E, Gariboldi V, Litzler PY, Anselme F, Babatasi G, Belin A, Garnier F, Bielefeld M, Hamon D, Lellouche N, Bourguignon T, Genet T, Eschalier R, D'Ostrevy N, Bories MC, Jouan J, Vanhuyse F, Blangy H, Doucerain J, Martins RP, Galand V. Suicide Attempts Among LVAD Recipients: Real-Life Data From the ASSIST-ICD Study. Circulation. 2020 Mar 17;141(11):934-936. doi: 10.1161/CIRCULATIONAHA.119.041910. Epub 2020 Mar 10. PMID: 32153210.

18: Nakagawa S, Uriel N, Prager KM. Should It Be Called "Suicide" or "Withdrawal of LVAD Support"? J Pain Symptom Manage. 2020 Nov;60(5):e1-e3. doi: 10.1016/j.jpainsymman.2020.08.021. Epub 2020 Aug 22. PMID: 32835832.

19: Gariboldi V, Grisoli D, Tarmiz A, Jaussaud N, Chalvignac V, Kerbaul F, Collart F. Mobile extracorporeal membrane oxygenation unit expands cardiac assist surgical programs. Ann Thorac Surg. 2010 Nov;90(5):1548-52. doi: 10.1016/j.athoracsur.2010.06.091. PMID: 20971261.

20: Rady MY, Verheijde JL. End-of-life discontinuation of destination therapy with cardiac and ventilatory support medical devices: physician-assisted death or allowing the patient to die? BMC Med Ethics. 2010 Sep 15;11:15. doi: 10.1186/1472-6939-11-15. Retraction in: BMC Med Ethics. 2010;11:20. PMID: 20843327; PMCID: PMC2949779.

21: Napp LC, Vogel-Claussen J, Schäfer A, Haverich A, Bauersachs J, Kühn C, Tongers J. First-in-Man Fully Percutaneous Complete Bypass of Heart and Lung. JACC Cardiovasc Interv. 2017 Dec 26;10(24):e231-e233. doi: 10.1016/j.jcin.2017.07.047. Epub 2017 Nov 29. PMID: 29198456.

22: Rygnestad T, Moen S, Wahba A, Lien S, Ingul CB, Schrader H, Knapstad SE, Severe poisoning with sotalol and verapamil. Recovery after 4 h of normothermic CPR followed by extra corporeal heart lung assist. Acta Anaesthesiol Scand. 2005 Oct;49(9):1378-80. doi: 10.1111/j.1399-6576.2005.00709.x. PMID: 16146479.

(VI) **Literature search on a potential association of ICD and suicide**

Data base: PubMed

Last data base query: 04.05.2023 ~11:30

Search algorithm: (suicide) AND (implantable cardioverter defibrillator)

1: McGeary A, Eldergill A. Medicolegal issues arising when pacemaker and implantable cardioverter defibrillator devices are deactivated in terminally ill patients. Med Sci Law. 2010 Jan;50(1):40-4. doi: 10.1258/msl.2009.009006. PMID: 20349694.

2: Pfeiffer D, Hagendorff A, Kühne C, Reinhardt S, Klein N. Implantierbarer Kardioverter-Defibrillator am Ende des Lebens [Implantable cardioverter-defibrillator at the end of life]. Herzschrittmacherther Elektrophysiol. 2015 Jun;26(2):134-40. German. doi: 10.1007/s00399-015-0366-6. Epub 2015 May 22. PMID: 26001358.

3: Daeschler M, Verdino RJ, Kirkpatrick JN. The ethics of unilateral implantable cardioverter defibrillators and cardiac resynchronization therapy with defibrillator deactivation: patient perspectives. Europace. 2017 Aug 1;19(8):1343-1348. doi: 10.1093/europace/euw227. PMID: 27915265.

4: Kirkpatrick JN, Gottlieb M, Sehgal P, Patel R, Verdino RJ. Deactivation of implantable cardioverter defibrillators in terminal illness and end of life care. Am J Cardiol. 2012 Jan 1;109(1):91-4. doi: 10.1016/j.amjcard.2011.08.011. Epub 2011 Sep 22. PMID: 21943937.

5: Lundberg AB, Bowen KP, Baumgart PM, Caplan JP. Phantom shocks and automated implantable cardioverter defibrillators. Psychosomatics. 2015 Jan-Feb;56(1):94-7. doi: 10.1016/j.psym.2014.09.005. Epub 2014 Sep 16. PMID: 25619677.

6: Mueller PS, Hook CC, Hayes DL. Ethical analysis of withdrawal of pacemaker or implantable cardioverter-defibrillator support at the end of life. Mayo Clin Proc. 2003 Aug;78(8):959-63. doi: 10.4065/78.8.959. PMID: 12911044.

7: Ballentine JM. Pacemaker and defibrillator deactivation in competent hospice patients: an ethical consideration. Am J Hosp Palliat Care. 2005 Jan-Feb;22(1):14-9. doi: 10.1177/104990910502200106. PMID: 15736602.

8: Sulmasy DP. Within you/without you: biotechnology, ontology, and ethics. J Gen Intern Med. 2008 Jan;23 Suppl 1(Suppl 1):69-72. doi:10.1007/s11606-007-0326-x. PMID: 18095048; PMCID: PMC2150626.

9: Ginwalla M, Battula S, Dunn J, Lewis WR. Termination of electrocution-induced ventricular fibrillation by an implantable cardioverter defibrillator. Pacing Clin Electrophysiol. 2010 Apr;33(4):510-2. doi:10.1111/j.1540-8159.2009.02607.x. Epub 2009 Dec 10. PMID: 20015136.

10: Kramer DB, Kesselheim AS, Salberg L, Brock DW, Maisel WH. Ethical and legal views regarding deactivation of cardiac implantable electrical devices in patients with hypertrophic cardiomyopathy. Am J Cardiol. 2011 Apr 1;107(7):1071-1075.e5. doi: 10.1016/j.amjcard.2010.11.036. Epub 2011 Feb 4. PMID: 21296323; PMCID: PMC3601901.

11: Doldi F, Reinke F, Yilmaz A, Eckardt L. Bullet-associated ventricular tachycardia: a case report. Eur Heart J Case Rep. 2021 Apr 26;5(4):ytab101. doi: 10.1093/ehjcr/ytab101. PMID: 34124548; PMCID: PMC8188868.

12: Kramer DB, Kesselheim AS, Brock DW, Maisel WH. Ethical and legal views of physicians regarding deactivation of cardiac implantable electrical devices: a quantitative assessment. Heart Rhythm. 2010 Nov;7(11):1537-42. doi: 10.1016/j.hrthm.2010.07.018. Epub 2010 Jul 19. PMID: 20650332; PMCID: PMC3001282.

13: Trappe HJ. Ethik in Intensivmedizin und Sterbehilfe : Zur Frage der Inaktivierung von Defibrillatoren vor dem Lebensende bei Schwerstkranken [Ethics in intensive care and euthanasia : With respect to inactivating defibrillators at the end of life in terminally ill patients]. Med Klin Intensivmed Notfmed. 2017 Apr;112(3):214-221. German. doi: 10.1007/s00063-015-0119-7. Epub 2015 Nov 17. PMID: 26577148.

14: Landry-Hould F, Mondésert B, Day AG, Ross HJ, Brouillette J, Clarke B, Zieroth S, Toma M, Parent MC, Fowler RA, You JJ, Ducharme A; DECIDE-HF investigators. Characteristics of Clinicians Are Associated With Their Beliefs About ICD Deactivation: Insight From the DECIDE-HF Study. CJC Open. 2021 Aug 8;3(8):994-1001. doi: 10.1016/j.cjco.2021.03.006. PMID: 34505038; PMCID: PMC8413241.

15: Goldstein NE, Mehta D, Siddiqui S, Teitelbaum E, Zeidman J, Singson M, Pe E, Bradley EH, Morrison RS. "That's like an act of suicide" patients' attitudes toward deactivation of implantable defibrillators. J Gen Intern Med. 2008 Jan;23 Suppl 1(Suppl 1):7-12. doi: 10.1007/s11606-007-0239-8. PMID: 18095037; PMCID: PMC2150628.

16: Cleland JG, Velavan P, Nasir M. Fighting against sudden death: a single or multidisciplinary approach. J Interv Card Electrophysiol. 2006 Dec;17(3):205-10. doi: 10.1007/s10840-006-9077-6. Epub 2007 Apr 6. PMID: 17415628.

17: Daeschler M, Verdino RJ, Caplan AL, Kirkpatrick JN. Defibrillator Deactivation against a Patient's Wishes: Perspectives of Electrophysiology Practitioners. Pacing Clin Electrophysiol. 2015 Aug;38(8):917-24. doi: 10.1111/pace.12614. Epub 2015 Mar 16. PMID: 25683098.

18: Yakut K, Erdoğan İ, Varan B, Atar İ. A Report of Brugada Syndrome Presenting with Cardiac Arrest Triggered by Verapamil Intoxication. Balkan Med J. 2017 Dec 1;34(6):576-579. doi: 10.4274/balkanmedj.2016.1301. PMID: 29215340; PMCID: PMC5785665.

19: Fuchs T. Suicide attempt aborted by an implantable defibrillator. Pacing Clin Electrophysiol. 2007 Aug;30(8):1020. doi: 10.1111/j.1540-8159.2007.00802.x. PMID: 17669087.

20: Enck RE. Management of cardiac devices as the end nears. Am J Hosp Palliat Care. 2005 Jan-Feb;22(1):7-8. doi: 10.1177/104990910502200101. PMID: 15736599.

21: Facciorusso A, Stanislao M, Fanelli M, Valori VM, Valle G. Ethical issues on defibrillator deactivation in end-of-life patients. J Cardiovasc Med (Hagerstown). 2011 Jul;12(7):498-500. doi: 10.2459/JCM.0b013e3283483724. PMID: 21610508.

22: Kokras N, Politis AM, Zervas IM, Pappa D, Markatou M, Katirtzoglou E, Papadimitriou GN. Cardiac rhythm management devices and electroconvulsive therapy: a critical review apropos of a depressed patient with a pacemaker. J ECT. 2011 Sep;27(3):214-20. doi: 10.1097/YCT.0b013e31820057b3. PMID: 21206373.

23: Huddle TS, Amos Bailey F. Pacemaker deactivation: withdrawal of support or active ending of life? Theor Med Bioeth. 2012 Dec;33(6):421-33. doi: 10.1007/s11017-012-9213-5. PMID: 22351107.

24: Rady MY, Verheijde JL. When is deactivating an implanted cardiac device physician-assisted death? Appraisal of the lethal pathophysiology and mode of death. J Palliat Med. 2011 Oct;14(10):1086-8; discussion 1089-90. doi: 10.1089/jpm.2011.0161. PMID: 22004145.

25: Derrickson AK, Baber JR, Agarwal A. A case of acute stress-induced ventricular tachycardia. Psychosom Med. 2007 Nov;69(8):825. doi: 10.1097/PSY.0b013e318159e7bd. Epub 2007 Oct 17. PMID: 17942830.

26: Goëb JL, Galloyer-Fortier A, Dupuis JM, Victor J, Gohier B, Garré JB. Conséquence psychiatrique après l'implantation d'un défibrillateur automatique. A propos d'un cas [Psychiatric complication of an implanted automatic defibrillator]. Arch Mal Coeur Vaiss. 2003 Dec;96(12):1235-8. French. PMID: 15248453.

27: Tomás CC, Oliveira E, Sousa D, Uba-Chupel M, Furtado G, Rocha C, Teixeira A, Ferreira P, Alves C, Gisin S, Catarino E, Carvalho N, Coucelo T, Bonfim L, Silva C, Franco D, González JA, Jardim HG, Silva R, Baixinho CL, Presado MªH, Marques MªF, Cardoso ME, Cunha M, Mendes J, Xavier A, Galhardo A, Couto M, Frade JG, Nunes C, Mesquita JR, Nascimento MS, Gonçalves G, Castro C, Mártires A, Monteiro MªJ, Rainho C, Caballero FP, Monago FM, Guerrero JT, Monago RM, Trigo AP, Gutierrez ML, Milanés GM, Reina MG, Villanueva AG, Piñero AS, Aliseda IR, Ramirez FB, Ribeiro A, Quelhas A, Manso C, Caballero FP, Guerrero JT, Monago FM, Santos RB, Jimenez NR, Nuñez CG, Gomez IR, Fernandez MªJL, Marquez LA, Moreno AL, Huertas MªJT, Ramirez FB, Seabra D, Salvador MªC, Braga L, Parreira P, Salgueiro-Oliveira A, Arreguy-Sena C, Oliveira BF, Henriques MªA, Santos J, Lebre S, Marques A, Festas C, Rodrigues S, Ribeiro A, Lumini J, Figueiredo AG, Hernandez-Martinez FJ, Campi L, Quintana-Montesdeoca MªP, Jimenez-Diaz JF, Rodriguez-De-Vera BC, Parente A, Mata MªA, Pereira AMª, Fernandes A, Brás M, Pinto MªR, Parreira P, Basto ML, Rei AC, Mónico LM, Sousa G, Morna C, Freitas O, Freitas G, Jardim A, Vasconcelos R, Horta LG, Rosa RS, Kranz LF, Nugem RC, Siqueira MS, Bordin R, Kniess R, Lacerda JT, Guedes J, Machado I, Almeida S, Zilhão A, Alves H, Ribeiro Ó, Amaral AP, Santos A, Monteiro J, Rocha MªC, Cruz R, Amaral AP, Lourenço M, Rocha MªC, Cruz R, Antunes S, Mendonça V, Andrade I, Osório N, Valado A, Caseiro A, Gabriel A, Martins AC, Mendes F, Cabral L, Ferreira M, Gonçalves A, Luz TD, Luz L, Martins R, Morgado A, Vale-Dias ML, Porta-Nova R, Fleig TC, Reuter ÉM, Froemming MB, Guerreiro SL, Carvalho LL, Guedelha D, Coelho P, Pereira A, Calha A, Cordeiro R, Gonçalves A, Certo A, Galvão A, Mata MªA, Welter A, Pereira E, Ribeiro S, Kretzer M, Jiménez-Díaz JF, Jiménez-Rodríguez C, Hernández-Martínez FJ, Rodríguez-De-Vera BDC, Marques-Rodrigues A, Coelho P, Bernardes T, Pereira A, Sousa P, Filho JG, Nazario N, Kretzer M, Amaral O, Garrido A, Veiga N, Nunes C, Pedro AR, Pereira C, Almeia A, Fernandes HM, Vasconcelos C, Sousa N, Reis VM, Monteiro MJ, Mendes R, Pinto IC, Pires T, Gama J, Preto V, Silva N, Magalhães C, Martins M, Duarte M, Paúl C, Martín I, Pinheiro AA, Xavier S, Azevedo J, Bento E, Marques C, Marques M, Macedo A, Pereira AT, Almeida JP, Almeida A, Alves J, Sousa N, Saavedra F, Mendes R, Maia AS, Oliveira MT, Sousa AR, Ferreira PP, Lopes LS, Santiago EC, Monteiro S, Jesus Â, Colaço A, Carvalho A, Silva RP, Cruz A, Ferreira A, Marques C, Figueiredo JP, Paixão S, Ferreira A, Lopes C, Moreira F, Figueiredo JP, Ferreira A, Ribeiro D, Moreira F, Figueiredo JP, Paixão S, Fernandes T, Amado D, Leal J, Azevedo M, Ramalho S, Mangas C, Ribeiro J, Gonçalves R, Nunes AF, Tuna AR, Martins CR, Forte HD, Costa C, Tenedório JA, Santana P, Andrade JA, Pinto JL, Campofiorito C, Nunes S, Carmo A, Kaliniczenco A, Alves B, Mendes F, Jesus C, Fonseca F, Gehrke F, Albuquerque C, Batista R, Cunha M, Madureira A, Ribeiro O, Martins R, Madeira T, Peixoto-Plácido C, Santos N, Santos O, Bergland A, Bye A, Lopes C, Alarcão V, Goulão B, Mendonça N, Nicola P, Clara JG, Gomes J, Querido A, Tomás C, Carvalho D, Cordeiro M, Rosa MC, Marques A, Brandão D, Ribeiro Ó, Araújo L, Paúl C, Minghelli B, Richaud S, Mendes AL, Marta-Simões J, Trindade IA, Ferreira C, Carvalho T, Cunha M, Pinto-Gouveia J, Fernandes MC, Rosa RS, Nugem RC, Kranz LF, Siqueira MS, Bordin R, Martins AC, Medeiros A, Pimentel R, Fernandes A, Mendonça C, Andrade I, Andrade S, Menezes RL, Bravo R, Miranda M, Ugartemendia L, Tena JMª, Pérez-Caballero FL, Fuentes-Broto L, Rodríguez AB, Carmen B, Carneiro MA, Domingues JN, Paixão S, Figueiredo J, Nascimento VB, Jesus C, Mendes F, Gehrke F, Alves B, Azzalis L, Fonseca F, Martins AR, Nunes A, Jorge A, Veiga N, Amorim A, Silva A, Martinho L, Monteiro L, Silva R, Coelho C, Amaral O, Coelho I, Pereira C, Correia A, Rodrigues D, Marante N, Silva P, Carvalho S, Araujo AR, Ribeiro M, Coutinho P, Ventura S, Roque F, Calvo C, Reses M, Conde J, Ferreira A, Figueiredo J, Silva D, Seiça L, Soares R, Mourão R, Kraus T, Abreu AC, Padilha JM, Alves JM, Sousa P, Oliveira M, Sousa J, Novais S, Mendes F, Pinto J, Cruz J, Marques A, Duarte H, Dixe MDA, Sousa P, Cruz I, Bastos F, Pereira F, Carvalho FL, Oliveira TT, Raposo VR, Rainho C, Ribeiro JC, Barroso I, Rodrigues V, Neves C, Oliveira TC, Oliveira B, Morais MªC, Baylina P, Rodrigues R, Azeredo Z, Vicente C, Dias H, Sim-Sim M, Parreira P, Salgueiro-Oliveira A, Castilho A, Melo R, Graveto J, Gomes J, Vaquinhas M, Carvalho C, Mónico L, Brito N, Sarroeira C, Amendoeira J, Cunha F, Cândido A, Fernandes P, Silva HR, Silva E, Barroso I, Lapa L, Antunes C, Gonçalves A, Galvão A, Gomes MªJ, Escanciano SR, Freitas M, Parreira P, Marôco J, Fernandes AR, Cabral C, Alves S, Sousa P, Ferreira A, Príncipe F, Seppänen UM, Ferreira M, Carvalhais M, Silva M, Ferreira M, Silva J, Neves J, Costa D, Santos B, Duarte S, Marques S, Ramalho S, Mendes I, Louro C, Menino E, Dixe M, Dias SS, Cordeiro M, Tomás C, Querido A, Carvalho D, Gomes J, Valim FC, Costa JO, Bernardes LG, Prebianchi H, Rosa

MC, Gonçalves N, Martins MM, Kurcgant P, Vieira A, Bento S, Deodato S, Rabiais I, Reis L, Torres A, Soares S, Ferreira M, Graça P, Leitão C, Abreu R, Bellém F, Almeida A, Ribeiro-Varandas E, Tavares A, Frade JG, Henriques C, Menino E, Louro C, Jordão C, Neco S, Morais C, Ferreira P, Silva CR, Brito A, Silva A, Duarte H, Dixe MDA, Sousa P, Postolache G, Oliveira R, Moreira I, Pedro L, Vicente S, Domingos S, Postolache O, Silva D, Filho JG, Nazario N, Kretzer M, Schneider D, Marques FM, Parreira P, Carvalho C, Mónico LM, Pinto C, Vicente S, Breda SJ, Gomes JH, Melo R, Parreira P, Salgueiro A, Graveto J, Vaquinhas M, Castilho A, Jesus Â, Duarte N, Lopes JC, Nunes H, Cruz A, Salgueiro-Oliveira A, Parreira P, Basto ML, Braga LM, Ferreira A, Araújo B, Alves JM, Ferreira M, Carvalhais M, Silva M, Novais S, Sousa AS, Ferrito C, Ferreira PL, Rodrigues A, Ferreira M, Oliveira I, Ferreira M, Neves J, Costa D, Duarte S, Silva J, Santos B, Martins C, Macedo AP, Araújo O, Augusto C, Braga F, Gomes L, Silva MA, Rosário R, Pimenta L, Carreira D, Teles P, Barros T, Tomás C, Querido A, Carvalho D, Gomes J, Cordeiro M, Carvalho D, Querido A, Tomás C, Gomes J, Cordeiro M, Jácome C, Marques A, Capelas S, Hall A, Alves D, Lousada M, Loureiro MªH, Camarneiro A, Silva M, Mendes A, Pedreiro A, G.Silva A, Coelho ES, Melo F, Ribeiro F, Torres R, Costa R, Pinho T, Jácome C, Marques A, Cruz B, Seabra D, Carreiras D, Ventura M, Cruz X, Brooks D, Marques A, Pinto MR, Parreira P, Lima-Basto M, Neves M, Mónico LM, Bizarro C, Cunha M, Galhardo A, Margarida C, Amorim AP, Silva E, Cruz S, Padilha JM, Valente J, Guerrero JT, Caballero FP, Santos RB, Gonzalez EP, Monago FM, Ugalde LU, Vélez MM, Tena MJ, Guerrero JT, Bravo R, Pérez-Caballero FL, Becerra IA, Agudelo MªE, Acedo G, Bajo R, Malheiro I, Gaspar F, Barros L, Furtado G, Uba-Chupel M, Marques M, Rama L, Braga M, Ferreira JP, Teixeira AMª, Cruz J, Barbosa T, Simões Â, Coelho L, Rodrigues A, Jiménez-Díaz JF, Martinez-Hernandez F, Rodriguez-De-Vera B, Ferreira P, Rodrigues A, Ramalho A, Petrica J, Mendes P, Serrano J, Santo I, Rosado A, Mendonça P, Freitas K, Ferreira D, Brito A, Fernandes R, Gomes S, Moreira F, Pinho C, Oliveira R, Oliveira AI, Mendonça P, Casimiro AP, Martins P, Silva I, Evangelista D, Leitão C, Velosa F, Carecho N, Coelho L, Menino E, Dixe A, Catarino H, Soares F, Gama E, Gordo C, Moreira E, Midões C, Santos M, Machado S, Oliveira VP, Santos M, Querido A, Dixe A, Marques R, Charepe Z, Antunes A, Santos S, Rosa MC, Rosa MC, Marques SF, Minghelli B, CaroMinghelli E, Luís MªJ, Brandão T, Mendes P, Marinho D, Petrica J, Monteiro D, Paulo R, Serrano J, Santo I, Monteiro L, Ramalho F, Santos-Rocha R, Morgado S, Bento T, Sousa G, Freitas O, Silva I, Freitas G, Morna C, Vasconcelos R, Azevedo T, Soares S, Pisco J, Ferreira PP, Olszewer EO, Oliveira MT, Sousa AR, Maia AS, Oliveira ST, Santos E, Oliveira AI, Maia C, Moreira F, Santos J, Mendes MF, Oliveira RF, Pinho C, Barreira E, Pereira A, Vaz JA, Novo A, Silva LD, Maia B, Ferreira E, Pires F, Andrade R, Camarinha L, Silva LD, Maia B, Ferreira E, Pires F, Andrade R, Camarinha L, César AF, Poço M, Ventura D, Loura R, Gomes P, Gomes C, Silva C, Melo E, Lindo J, Domingos J, Mendes Z, Poeta S, Carvalho T, Tomás C, Catarino H, Dixe MªA, Ramalho A, Rosado A, Mendes P, Paulo R, Garcia I, Petrica J, Rodrigues S, Meneses R, Afonso C, Faria L, Seixas A, Cordeiro M, Granjo P, Gomes JC, Souza NR, Furtado GE, Rocha SV, Silva P, Carvalho J, Morais MA, Santos S, Lebre P, Antunes A, Calha A, Xavier A, Cunha M, Pinto-Gouveia J, Alencar L, Cunha M, Madureira A, Cardoso I, Galhardo A, Daniel F, Rodrigues V, Luz L, Luz T, Ramos MR, Medeiros DC, Carmo BM, Seabra A, Padez C, Silva MC, Rodrigues A, Coelho P, Coelho A, Caminha M, Matheus F, Mendes E, Correia J, Kretzer M, Hernandez-Martinez FJ, Jimenez-Diaz JF, Rodriguez-De-Vera BC, Jimenez-Rodriguez C, Armas-Gonzalez Y, Rodrigues C, Pedroso R, Apolinário-Hagen J, Vehreschild V, Veloso M, Magalhães C, Cabral I, Ferraz M, Nave F, Costa E, Matos F, Pacheco J, Dias A, Pereira C, Duarte J, Cunha M, Silva D, Mónico LM, Alferes VR, Brêda MªSJ, Carvalho C, Parreira PM, Morais MªC, Ferreira P, Pimenta R, Boavida J, Pinto IC, Pires T, Silva C, Ribeiro M, Viega-Branco M, Pereira F, Pereira AMª, Almeida FM, Estevez GL, Ribeiro S, Kretzer MR, João PV, Nogueira P, Novais S, Pereira A, Carneiro L, Mota M, Cruz R, Santiago L, Fontes-Ribeiro C, Furtado G, Rocha SV, Coutinho AP, Neto JS, Vasconcelos LR, Souza NR, Dantas E, Dinis A, Carvalho S, Castilho P, Pinto-Gouveia J, Sarreira-Santos A, Figueiredo A, Medeiros-Garcia L, Seabra P, Rodrigues R, Morais MªC, Fernandes PO, Santiago C, Figueiredo MªH, Basto ML, Guimarães T, Coelho A, Graça A, Silva AM, Fonseca AR, Vale-Dias L, Minas B, Franco-Borges G, Simões C, Santos S, Serra A, Matos M, Jesus L, Tavares AS, Almeida A, Leitão C, Varandas E, Abreu R, Bellém F, Trindade IA, Ferreira C, Pinto-Gouveia J, Marta-Simões J, Amaral O, Miranda C, Guimarães P, Gonçalves R, Veiga N, Pereira C, Fleig TC, San-Martin EA, Goulart CL, Schneiders PB, Miranda NF, Carvalho LL, Silva AG, Topa J, Nogueira C, Neves S, Ventura R, Nazaré C, Brandão D, Freitas A, Ribeiro Ó, Paúl C, Mercê C, Branco M, Almeida P, Nascimento D, Pereira J, Catela D, Rafael H, Reis AC, Mendes A, Valente AR, Lousada M, Sousa D, Baltazar AL, Loureiro MªH, Oliveira A, Aparício J, Marques A, Marques A, Oliveira A, Neves J, Ayoub R, Sousa L, Marques-Vieira C, Severino S, José H, Cadorio I, Lousada M, Cunha M, Andrade D, Galhardo A, Couto M, Mendes F, Domingues C, Schukg S, Abrantes AM, Gonçalves AC, Sales T, Teixo R, Silva R, Estrela J, Laranjo M, Casalta-Lopes J, Rocha C, Simões PC, Sarmento-Ribeiro AB, Botelho MªF, Rosa MS, Fonseca V, Colaço D, Neves V, Jesus C, Hesse C, Rocha C, Osório N, Valado A, Caseiro A, Gabriel A, Svensson L, Mendes F, Siba WA, Pereira C, Tomaz J, Carvalho T, Pinto-Gouveia J, Cunha M, Duarte D, Lopes NV, Fonseca-Pinto R, Duarte D, Lopes NV, Fonseca-Pinto R, Martins AC, Brandão P, Martins L, Cardoso M, Morais N, Cruz J, Alves N, Faria P, Mateus A, Morouço P, Alves N, Ferreira N, Mateus A, Faria P, Morouço P, Malheiro I, Gaspar F, Barros L, Parreira P, Cardoso A, Mónico L, Carvalho C, Lopes A, Salgueiro-Oliveira A, Seixas A, Soares V, Dias T, Vardasca R, Gabriel J, Rodrigues S, Paredes H, Reis A, Marinho S, Filipe V, Lains J, Barroso J, Da Motta C, Carvalho CB, Pinto-Gouveia J, Peixoto E, Gomes AA, Costa V, Couto D, Marques DR, Leitão JA, Tavares J, Azevedo MH, Silva CF, Freitas J, Parreira P, Marôco J, Garcia-Gordillo MA, Collado-Mateo D, Chen G, Iezzi A, Sala JA, Parraça JA, Gusi N, Sousa J, Marques M, Jardim J, Pereira A, Simões S, Cunha M, Sardo P, Guedes J, Lindo J, Machado P, Melo E, Carvalho CB, Benevides J, Sousa

M, Cabral J, Da Motta C, Pereira AT, Xavier S, Azevedo J, Bento E, Marques C, Carvalho R, Marques M, Macedo A, Silva AM, Alves J, Gomes AA, Marques DR, Azevedo MªH, Silva C, Mendes A, Lee HD, Spolaôr N, Oliva JT, Chung WF, Fonseca-Pinto R, Bairros K, Silva CD, Souza CA, Schroeder SS, Araújo E, Monteiro H, Costa R, Dias SS, Torgal J, Henriques CG, Santos L, Caceiro EF, Ramalho SA, Oliveira R, Afreixo V, Santos J, Mota P, Cruz A, Pimentel F, Marques R, Dixe MªA, Querido A, Sousa P, Benevides J, Da Motta C, Sousa M, Caldeira SN, Carvalho CB, Querido A, Tomás C, Carvalho D, Gomes J, Cordeiro M, Costa JO, Valim FC, Ribeiro LC, Charepe Z, Querido A, Figueiredo MªH, Aquino PS, Ribeiro SG, Pinheiro AB, Lessa PA, Oliveira MF, Brito LS, Pinto ÍN, Furtado AS, Castro RB, Aquino CQ, Martins ES, Pinheiro AB, Aquino PS, Oliveira LL, Pinheiro PC, Sousa CR, Freitas VA, Silva TM, Lima AS, Aquino CQ, Andrade KV, Oliveira CA, Vidal EF, Ganho-Ávila A, Moura-Ramos M, Gonçalves Ó, Almeida J, Silva A, Brito I, Amado J, Rodrigo A, Santos S, Gomes F, Rosa MC, Marques SF, Luís S, Cavalheiro L, Ferreira P, Gonçalves R, Lopes RS, Cavalheiro L, Ferreira P, Gonçalves R, Fiorin BH, Santos MS, Oliveira ES, Moreira RL, Oliveira EA, Filho BL, Palmeira L, Garcia T, Pinto-Gouveia J, Cunha M, Cardoso S, Palmeira L, Cunha M, Pinto-Gouveia J, Marta-Simões J, Mendes AL, Trindade IA, Oliveira S, Ferreira C, Mendes AL, Marta-Simões J, Trindade IA, Ferreira C, Nave F, Campos M, Gaudêncio I, Martins F, Ferreira L, Lopes N, Fonseca-Pinto R, Rodrigues R, Azeredo Z, Vicente C, Silva J, Sousa P, Marques R, Mendes I, Rodrigues R, Azeredo Z, Vicente C, Vardasca R, Marques AR, Seixas A, Carvalho R, Gabriel J, Ferreira PP, Oliveira MT, Sousa AR, Maia AS, Oliveira ST, Costa PO, Silva MM, Arreguy-Sena C, Alvarenga-Martins N, Pinto PF, Oliveira DC, Parreira PD, Gomes AT, Braga LM, Araújo O, Lage I, Cabrita J, Teixeira L, Marques R, Dixe MªA, Querido A, Sousa P, Silva S, Cordeiro E, Pimentel J, Ferro-Lebres V, Souza JA, Tavares M, Dixe MªA, Sousa P, Passadouro R, Peralta T, Ferreira C, Lourenço G, Serrano J, Petrica J, Paulo R, Honório S, Mendes P, Simões A, Carvalho L, Pereira A, Silva S, Sousa P, Padilha JM, Figueiredo D, Valente C, Marques A, Ribas P, Sousa J, Brandão F, Sousa C, Martins M, Sousa P, Marques R, Mendes F, Fernandes R, Martins E, Magalhães C, Araújo P, Grande C, Mata MªA, Vieitez JG, Bianchini B, Nazario N, Filho JG, Kretzer M, Costa T, Almeida A, Baffour G, Almeida A, Costa T, Baffour G, Azeredo Z, Laranjeira C, Guerra M, Barbeiro AP, Ferreira R, Lopes S, Nunes L, Mendes A, Martins J, Schneider D, Kretzer M, Magajewski F, Soares C, Marques A, Batista M, Castuera RJ, Mesquita H, Faustino A, Santos J, Honório S, Vizzotto BP, Frigo L, Pivetta HF, Sardo D, Martins C, Abreu W, Figueiredo MªC, Batista M, Jimenez-Castuera R, Petrica J, Serrano J, Honório S, Paulo R, Mendes P, Sousa P, Marques R, Faustino A, Silveira P, Serrano J, Paulo R, Mendes P, Honório S, Oliveira C, Bastos F, Cruz I, Rodriguez CK, Kretzer MR, Nazário NO, Cruz P, Vaz DC, Ruben RB, Avelelas F, Silva S, Campos MªJ, Almeida M, Gonçalves L, Antunes L, Sardo P, Guedes J, Simões J, Machado P, Melo E, Cardoso S, Santos O, Nunes C, Loureiro I, Santos F, Alves G, Soar C, Marsi TO, Silva E, Pedrosa D, Leça A, Silva D, Galvão A, Gomes M, Fernandes P, Noné A, Combadão J, Ramalhete C, Figueiredo P, Caeiro P, Fontana KC, Lacerda JT, Machado PO, Borges R, Barbosa F, Sá D, Brunhoso G, Aparício G, Carvalho A, Garcia AP, Fernandes PO, Santos A, Veiga N, Brás C, Carvalho I, Batalha J, Glória M, Bexiga F, Coelho I, Amaral O, Pereira C, Pinho C, Paraíso N, Oliveira AI, Lima CF, Dias AP, Silva P, Espada M, Marques M, Pereira A, Pereira AMª, Veiga-Branco Mª, Pereira F, Ribeiro M, Lima V, Oliveira AI, Pinho C, Cruz G, Oliveira RF, Barreiros L, Moreira F, Camarneiro A, Loureiro MªH, Silva M, Duarte C, Jesus Â, Cruz A, Mota M, Novais S, Nogueira P, Pereira A, Carneiro L, João PV, Lima TM, Salgueiro-Oliveira A, Vaquinhas M, Parreira P, Melo R, Graveto J, Castilho A, Gomes JH, Medina MS, Blanco VG, Santos O, Lopes E, Virgolino A, Dinis A, Ambrósio S, Almeida I, Marques T, Heitor MªJ, Garcia-Gordillo MA, Collado-Mateo D, Olivares PR, Parraça JA, Sala JA, Castilho A, Graveto J, Parreira P, Oliveira A, Gomes JH, Melo R, Vaquinhas M, Cheio M, Cruz A, Pereira OR, Pinto S, Oliveira A, Manso MC, Sousa C, Vinha AF, Machado MªM, Vieira M, Fernandes B, Tomás T, Quirino D, Desouzart G, Matos R, Bordini M, Mouroço P, Matos AR, Serapioni M, Guimarães T, Fonseca V, Costa A, Ribeiro J, Lobato J, Martin IZ, Björklund A, Tavares AI, Ferreira P, Passadouro R, Morgado S, Tavares N, Valente J, Martins AC, Araújo P, Fernandes R, Mendes F, Magalhães C, Martins E, Mendes P, Paulo R, Faustino A, Mesquita H, Honório S, Batista M, Lacerda JT, Ortiga AB, Calvo MªC, Natal S, Pereira M, Ferreira M, Prata AR, Nelas P, Duarte J, Carneiro J, Oliveira AI, Pinho C, Couto C, Oliveira RF, Moreira F, Maia AS, Oliveira MT, Sousa AR, Ferreira PP, Souza GM, Almada LF, Conceição MA, Santiago EC, Rodrigues S, Domingues G, Ferreira I, Faria L, Seixas A, Costa AR, Jesus Â, Cardoso A, Meireles A, Colaço A, Cruz A, Vieira VL, Vincha KR, Cervato-Mancuso AMª, Faria M, Reis C, Cova MP, Ascenso RT, Almeida HA, Oliveira EG, Santana M, Pereira R, Oliveira EG, Almeida HA, Ascenso RT, Jesus R, Tapadas R, Tim-Tim C, Cezanne C, Lagoa M, Dias SS, Torgal J, Lopes J, Almeida H, Amado S, Carrão L, Cunha M, Saboga-Nunes L, Albuquerque C, Ribeiro O, Oliveira S, Morais MªC, Martins E, Mendes F, Fernandes R, Magalhães C, Araújo P, Pedro AR, Amaral O, Escoval A, Assunção V, Luís H, Luís L, Apolinário-Hagen J, Vehreschild V, Fotschl U, Lirk G, Martins AC, Andrade I, Mendes F, Mendonça V, Antunes S, Andrade I, Osório N, Valado A, Caseiro A, Gabriel A, Martins AC, Mendes F, Silva PA, Mónico LM, Parreira PM, Carvalho C, Carvalho C, Parreira PM, Mónico LM, Ruivo J, Silva V, Sousa P, Padilha JM, Ferraz V, Aparício G, Duarte J, Vasconcelos C, Almeida A, Neves J, Correia T, Amorim H, Mendes R, Saboga-Nunes L, Cunha M, Albuquerque C, Pereira ES, Santos LS, Reis AS, Silva HR, Rombo J, Fernandes JC, Fernandes P, Ribeiro J, Mangas C, Freire A, Silva S, Francisco I, Oliveira A, Catarino H, Dixe MªA, Louro MªC, Lopes S, Dixe A, Dixe MªA, Menino E, Catarino H, Soares F, Oliveira AP, Gordo S, Kraus T, Tomás C, Queirós P, Rodrigues T, Sousa P, Frade JG, Lobão C, Moura CB, Dreyer LC, Meneghetti V, Cabral PP, Pinto F, Sousa P, Esteves MªR, Galvão S, Tytgat I, Andrade I, Osório N, Valado A, Caseiro A, Gabriel A, Martins AC, Mendes F, Casas-Novas M, Bernardo H, Andrade I, Sousa G, Sousa AP, Rocha C, Belo P, Osório N, Valado A, Caseiro A, Gabriel A, Martins AC, Mendes F, Martins F, Pulido-Fuentes M, Barroso I, Cabral G, Monteiro MJ, Rainho C, Prado A, Carvalho YM, Campos M, Moreira L, Ferreira J, Teixeira

A, Rama L, Campos M, Moreira L, Ferreira J, Teixeira A, Rama L. Proceedings of the 3rd IPLeiria's International Health Congress : Leiria, Portugal. 6-7 May 2016. BMC Health Serv Res. 2016 Jul 6;16 Suppl 3(Suppl 3):200. doi: 10.1186/s12913-016-1423-5. PMID: 27409075; PMCID: PMC4943498.

(VII) **Literature search on a potential association between PM and suicide**

Data base: PubMed

Last data base query: 03.05.2023 ~17:45

Search algorithm: (suicide) AND (pacemaker)

1: Ballentine JM. Pacemaker and defibrillator deactivation in competent hospice patients: an ethical consideration. Am J Hosp Palliat Care. 2005 Jan-Feb;22(1):14-9. doi: 10.1177/104990910502200106. PMID: 15736602.

2: Menon MS, Kumar P, Jayachandran CI. Clinical Profile and Management of Poisoning with Suicide Tree: An Observational Study. Heart Views. 2016 Oct-Dec;17(4):136-139. doi: 10.4103/1995-705X.201783. PMID: 28400936; PMCID: PMC5363088.

3: McGeary A, Eldergill A. Medicolegal issues arising when pacemaker and implantable cardioverter defibrillator devices are deactivated in terminally ill patients. Med Sci Law. 2010 Jan;50(1):40-4. doi: 10.1258/msl.2009.009006. PMID: 20349694.

4: Harter TD, Sterenson EL, Borgert A, Rasmussen C. Perceptions of Medical Providers on Morality and Decision-Making Capacity in Withholding and Withdrawing Life-Sustaining Treatment and Suicide. AJOB Empir Bioeth. 2021 Oct-Dec;12(4):227-238. doi: 10.1080/23294515.2021.1887961. Epub 2021 Mar 15. PMID: 33719891.

5: Pallangyo P, Mgopa L, Millinga J, Bhalia S, Hemed NR, Mkojera Z, Swai HJ, Seraphine P, Mulashani R, Ndelwa B, Shemu T, Janabi M. Suicide Attempt Following Pacemaker Implantation in an Eighty-Three-Year-Old Male: A Case Report. J Med Cases. 2019 Dec;10(12):345-347. doi: 10.14740/jmc3383. Epub 2019 Dec 31. PMID: 34434306; PMCID: PMC8383566.

6: Menezes RG, Usman MS, Hussain SA, Madadin M, Siddiqi TJ, Fatima H, Ram P, Pasha SB, Senthilkumaran S, Fatima TQ, Luis SA. Cerbera odollam toxicity: A review. J Forensic Leg Med. 2018 Aug;58:113-116. doi: 10.1016/j.jflm.2018.05.007. Epub 2018 May 9. PMID: 29778924.

7: Masoom H, Berbenetz NM, Chow J, Acosta JG, Amin F. Suicide Attempt by Self-Dissection of Permanent Pacemaker Leads. JACC Case Rep. 2020 Feb 19;2(2):296-299. doi: 10.1016/j.jaccas.2019.11.070. PMID: 34317227; PMCID: PMC8298311.

8: Che X, Abdelwahed YS, Wang X, Fang Y, Wang L. Pacemaker implantation in patients with major depression, should it be of concern? A case report and literature review. BMC Cardiovasc Disord. 2020 Jun 9;20(1):279. doi: 10.1186/s12872-020-01565-3. PMID: 32517791; PMCID: PMC7285466.

9: Dulski J, Cerquera-Cleves C, Milanowski L, Kidd A, Sitek EJ, Strongosky A, Vanegas Monroy AM, Dickson DW, Ross OA, Pentela-Nowicka J, Sławek J, Wszolek ZK. Clinical, pathological and genetic characteristics of Perry disease-new cases and literature review. Eur J Neurol. 2021 Dec;28(12):4010-4021. doi: 10.1111/ene.15048. Epub 2021 Aug 26. PMID: 34342072; PMCID: PMC9295182.

10: Norgaard ML, Melchior T, Wagner T, Haugan K. Suicide attempt by complete self-removal of a 12-year-old permanent pacemaker system: case report. J Cardiovasc Electrophysiol. 2014 Jan;25(1):99-100. doi: 10.1111/jce.12295. Epub 2013 Oct 10. PMID: 24102772.

11: Avci A, Yilmaz A, Celik M, Demir K, Keles F. Successful treatment of suicide attempt by megadose of propafenone and captopril. Cardiovasc Toxicol. 2013 Sep;13(3):230-3. doi: 10.1007/s12012-013-9201-7. PMID: 23397376.

12: Bordier P, Robert F. Suicide by self-removal of a pacemaker. Am J Forensic Med Pathol. 2004 Mar;25(1):78-9. doi: 10.1097/01.paf.0000113858.98640.d9. PMID: 15075695.

13: Lathers CM, Schraeder PL. Clinical pharmacology: drugs as a benefit and/or risk in sudden unexpected death in epilepsy? J Clin Pharmacol. 2002 Feb;42(2):123-36. doi: 10.1177/00912700222011157. PMID: 11831534.

14: Harrigan RA, Perron AD, Brady WJ. Atrioventricular dissociation. Am J Emerg Med. 2001 May;19(3):218-22. doi: 10.1053/ajem.2001.22657. PMID: 11326350.

15: Lee-Jones M, Bennett MA, Sherwell JM. Cyanide self-poisoning. Br Med J. 1970 Dec 26;4(5738):780-1. doi: 10.1136/bmj.4.5738.780. PMID: 5497407; PMCID: PMC1820402.

16: Rothenhäusler HB, Hoberl C, Ehrentrout S, Kapfhammer HP, Weber MM. Suicide attempt by pure citalopram overdose causing long-lasting severe sinus bradycardia, hypotension and syncopes: successful therapy with a temporary pacemaker. Pharmacopsychiatry. 2000 Jul;33(4):150-2. doi: 10.1055/s-2000-11225. PMID: 10958266.

17: Alarfaj M, Goswami A. Cardiotoxicity in yew berry poisoning. Am J Emerg Med. 2021 Dec;50:812.e1-812.e4. doi: 10.1016/j.ajem.2021.05.043. Epub 2021 May 18. PMID: 34049761.

18: Simon AB, Kleinman P, Janz N. Suicide attempt by pacemaker system abuse: a case report with comments on the psychological adaptation of pacemaker patients. Pacing Clin Electrophysiol. 1980 Mar;3(2):224-8. doi: 10.1111/j.1540-8159.1980.tb04333.x. PMID: 6160513.

19: Wu VC, Chang SH, Kuo CF, Liu JR, Chen SW, Yeh YH, Luo SF, See LC. Suicide death rates in patients with cardiovascular diseases - A 15-year nationwide cohort study in Taiwan. J Affect Disord. 2018 Oct 1;238:187-193. doi: 10.1016/j.jad.2018.05.046. Epub 2018 Jun 1. PMID: 29885608.

20: Leya F, Tuchek JM, Coats W. Abnormal distortion of aortic corevalve bioprosthesis with suicide left ventricle, aortic insufficiency, and severe mitral regurgitation during transcatheter aortic valve replacement. Catheter Cardiovasc Interv. 2016 Dec;88(7):1181-1187. doi: 10.1002/ccd.26463. Epub 2016 Mar 4. PMID: 26945836.

21: Menchaca K, Ostos Perez CA, Draguljevic N, Isaac S. Management Challenge: An Atypical Variant of Takotsubo Presenting With Multiple Complications. Cureus. 2022 Jul 14;14(7):e26836. doi: 10.7759/cureus.26836. PMID: 35854952; PMCID: PMC9286025.

22: Watling SM, Crain JL, Edwards TD, Stiller RA. Verapamil overdose: case report and review of the literature. Ann Pharmacother. 1992 Nov;26(11):1373-8. doi: 10.1177/106002809202601106. PMID: 1477440.

23: Antunes MJ, Prieto D, Sola E, Antunes PE, De Oliveira JF, Franco F, Providência LA. Cardiac transplantation: five years' activity. Rev Port Cardiol. 2010 May;29(5):731-48. English, Portuguese. PMID: 20866004.

24: Kokras N, Politis AM, Zervas IM, Pappa D, Markatou M, Katirtzoglou E, Papadimitriou GN. Cardiac rhythm management devices and electroconvulsive therapy: a critical review apropos of a depressed patient with a pacemaker. J ECT. 2011 Sep;27(3):214-20. doi: 10.1097/YCT.0b013e31820057b3. PMID: 21206373.

25: Daeschler M, Verdino RJ, Caplan AL, Kirkpatrick JN. Defibrillator Deactivation against a Patient's Wishes: Perspectives of Electrophysiology Practitioners. Pacing Clin Electrophysiol. 2015 Aug;38(8):917-24. doi: 10.1111/pace.12614. Epub 2015 Mar 16. PMID: 25683098.

26: Rosenthal R, Crisafi BR, Coomaraswamy RP. Manual extraction of a permanent pacemaker: an attempted suicide. Pacing Clin Electrophysiol. 1980 Mar;3(2):229-31. doi: 10.1111/j.1540-8159.1980.tb04334.x. PMID: 6160514.

27: Limke KK, Shelton AR, Elliott ES. Fluvoxamine interaction with warfarin. Ann Pharmacother. 2002 Dec;36(12):1890-2. doi: 10.1345/aph.1C112. PMID: 12452751.

28: Kramer DB, Kesselheim AS, Salberg L, Brock DW, Maisel WH. Ethical and legal views regarding deactivation of cardiac implantable electrical devices in patients with hypertrophic cardiomyopathy. Am J Cardiol. 2011 Apr 1;107(7):1071-1075.e5. doi: 10.1016/j.amjcard.2010.11.036. Epub 2011 Feb 4. PMID: 21296323; PMCID: PMC3601901.

29: Kramer DB, Kesselheim AS, Brock DW, Maisel WH. Ethical and legal views of physicians regarding deactivation of cardiac implantable electrical devices: a quantitative assessment. Heart Rhythm. 2010 Nov;7(11):1537-42. doi: 10.1016/j.hrthm.2010.07.018. Epub 2010 Jul 19. PMID: 20650332; PMCID: PMC3001282.

30: Hochmeister MN, Seifert D, Smetana R, Czernin J. Suicide attempted by aiming slaughtering gun at pacemaker. Am J Forensic Med Pathol. 1989 Sep;10(3):268. doi: 10.1097/00000433-198909000-00043. PMID: 2782308.

31: Burgess KR, Jefferis RW, Stevenson IF. Fatal thioridazine cardiotoxicity. Med J Aust. 1979 Aug 25;2(4):177-8. doi: 10.5694/j.1326-5377.1979.tb142025.x. PMID: 514124.

32: Mueller PS, Hook CC, Hayes DL. Ethical analysis of withdrawal of pacemaker or implantable cardioverter-defibrillator support at the end of life. Mayo Clin Proc. 2003 Aug;78(8):959-63. doi: 10.4065/78.8.959. PMID: 12911044.

33: Gómez Casal V, Lage Cendon L, Lago Preciado G, Vara Adrio S. Intoxicación por ivabradina con ideación autolítica [Ivabradine poisoning with suicide intention]. Med Intensiva. 2015 Dec;39(9):577-9. Spanish. doi: 10.1016/j.medin.2015.04.008. Epub 2015 Jul 21. PMID: 26208765.

34: Huddle TS, Amos Bailey F. Pacemaker deactivation: withdrawal of support or active ending of life? Theor Med Bioeth. 2012 Dec;33(6):421-33. doi: 10.1007/s11017-012-9213-5. PMID: 22351107.

35: Taboulet P, Baud FJ, Bismuth C, Vicaut E. Acute digitalis intoxication - is pacing still appropriate? J Toxicol Clin Toxicol. 1993;31(2):261-73. doi: 10.3109/15563659309000393. PMID: 8492339.

36: Naseem S, Munaf S. Suicidal Ideation, Depression, Anxiety, Stress, And Life Satisfaction Of Medical, Engineering, And Social Sciences Students. J Ayub Med Coll Abbottabad. 2017 Jul-Sep;29(3):422-427. PMID: 29076675.

37: Bagian JP, Gosbee J, Lee CZ, Williams L, McKnight SD, Mannos DM. The Veterans Affairs root cause analysis system in action. Jt Comm J Qual Improv. 2002 Oct;28(10):531-45. doi: 10.1016/s1070-3241(02)28057-8. PMID: 12369156.

38: Streit S, Walpoth N, Windecker S, Meier B, Hess O. Is alcohol ablation of the septum associated with recurrent tachyarrhythmias? Swiss Med Wkly. 2007 Dec 1;137(47-48):660-8. doi: 10.4414/smw.2007.11941. PMID: 18058274.

39: Koschny R, Lutz M, Seckinger J, Schwenger V, Stremmel W, Eisenbach C. Extracorporeal life support and plasmapheresis in a case of severe polyintoxication. J Emerg Med. 2014 Nov;47(5):527-31. doi: 10.1016/j.jemermed.2014.04.044. Epub 2014 Sep 12. PMID: 25220022.

40: Caci H, Robert P, Dossios C, Boyer P. L'échelle de matinalité pour enfants et adolescents: propriétés psychométriques et effet du mois de naissance [Morningness-Eveningness for Children Scale: psychometric properties and month of birth effect]. Encephale. 2005 Jan-Feb;31(1 Pt 1):56-64. French. doi: 10.1016/s0013-7006(05)82372-3. PMID: 15971640.

41: Krappweis J, Petereit G, Justus J, Altmann E, Kirch W. Digitoxin intoxication with lethal outcome. Eur J Med Res. 1996 Nov 25;1(12):551-3. PMID: 9438160.

42: Aggarwal N, Kupfer Y, Seneviratne C, Tessler S. Methylene blue reverses recalcitrant shock in β-blocker and calcium channel blocker overdose. BMJ Case Rep. 2013 Jan 18;2013:bcr2012007402. doi: 10.1136/bcr-2012-007402. PMID: 23334490; PMCID: PMC3604019.

43: Pap I, Kertész J, Sági I. Ongyilkossági kísérlet Verapamillal [Attempted suicide with verapamil]. Orv Hetil. 1989 Apr 2;130(14):735-6. Hungarian. PMID: 2657556.

44: Souêtre E, Salvati E, Darcourt G. Le concept de rythme biologique en psychopathologie [The concept of biological rhythm in psychopathology]. Rev Electroencephalogr Neurophysiol Clin. 1987 Dec;17(4):359-76. French. doi: 10.1016/s0370-4475(87)80083-7. PMID: 3326073.

45: Juneja D, Singh O, Bhasin A, Gupta M, Saxena S, Chaturvedi A. Severe suicidal digoxin toxicity managed with resin hemoperfusion: A case report. Indian J Crit Care Med. 2012 Oct;16(4):231-3. doi: 10.4103/0972-5229.106511. PMID: 23559736; PMCID: PMC3610461.

46: Ciszowski K, Winnik L, Groszek B, Kłys M, Kołodziej J. Ostre zatrucie chlorochina--rzadkie, ale zawsze powazne: opis przypadków i przeglad literatury [Acute chloroquine intoxication--rare, but always serious: case reports and literature review]. Przegl Lek. 2005;62(6):501-7. Polish. PMID: 16225106.

47: Yakut K, Erdoğan İ, Varan B, Atar İ. A Report of Brugada Syndrome Presenting with Cardiac Arrest Triggered by Verapamil Intoxication. Balkan Med J. 2017 Dec 1;34(6):576-579. doi: 10.4274/balkanmedj.2016.1301. PMID: 29215340; PMCID:

PMC5785665.

48: Daeschler M, Verdino RJ, Kirkpatrick JN. The ethics of unilateral implantable cardioverter defibrillators and cardiac resynchronization therapy with defibrillator deactivation: patient perspectives. Europace. 2017 Aug 1;19(8):1343-1348. doi: 10.1093/europace/euw227. PMID: 27915265.

49: Türe M, Bilici M, Akın A, Demir F, Balık H, Darakçı SM. Complete atrioventricular block associated with clozapine intoxication: case report. Turk J Pediatr. 2019;61(4):618-621. doi: 10.24953/turkjped.2019.04.024. PMID: 31990485.

50: Mauskopf JA, Wenger TL. Cost-effectiveness analysis of the use of digoxin immune Fab (ovine) for treatment of digoxin toxicity. Am J Cardiol. 1991 Dec 15;68(17):1709-14. doi: 10.1016/0002-9149(91)90334-h. PMID: 1746476.

51: Enck RE. Management of cardiac devices as the end nears. Am J Hosp Palliat Care. 2005 Jan-Feb;22(1):7-8. doi: 10.1177/104990910502200101. PMID: 15736599.

52: Harthorne JW. Attempted suicide by self-removal of implanted pacemaker. Pacing Clin Electrophysiol. 1980 Nov;3(6):740-1. doi: 10.1111/j.1540-8159.1980.tb05581.x. PMID: 6161359.

53: Eibs HG, Oberdisse U, Brambach U. Intoxikation mit Beta-Rezeptorenblockern [Intoxication with beta-receptor blockers (author's transl)]. Dtsch Med Wochenschr. 1982 Jul 23;107(29-30):1139-43. German. doi: 10.1055/s-2008-1070090. PMID: 6177490.

54: Wallin CJ, Hulting J. Massive metoprolol poisoning treated with prenalterol. Acta Med Scand. 1983;214(3):253-5. doi: 10.1111/j.0954-6820.1983.tb08604.x. PMID: 6660032.

55: Wharton CF. Attempted suicide by digoxin self administration and its management. Guys Hosp Rep. 1970;119(3):243-51. PMID: 5491307.

56: Bharadwaj P, Ward KT. Ethical considerations of patients with pacemakers. Am Fam Physician. 2008 Aug 1;78(3):398-9. PMID: 18711956.

57: Ahlmark G. Extreme digitalis intoxication. Acta Med Scand. 1976;200(5):423-5. doi: 10.1111/j.0954-6820.1976.tb08256.x. PMID: 983814.

58: Chen JY, Liu PY, Chen JH, Lin LJ. Safety of transvenous temporary cardiac pacing in patients with accidental digoxin overdose and symptomatic bradycardia. Cardiology. 2004;102(3):152-5. doi: 10.1159/000080483. Epub 2004 Aug 27. PMID: 15334025.

59: Friend DG. Digitalis after two centuries (William Withering). Arch Surg. 1976 Jan;111(1):14-9. doi: 10.1001/archsurg.1976.01360190016002. PMID: 1106355.

60: Bismuth C, Motte G, Conso F, Chauvin M, Gaultier M. Acute digitoxin intoxication treated by intracardiac pacemaker: experience in sixty-eight patients. Clin Toxicol. 1977;10(4):443-56. doi: 10.3109/15563657709046282. PMID: 862379.

61: Lillehei CW, Varco RL, Cohen M, Warden HE, Gott VL, DeWall RA, Patton C, Moller JH. The first open heart corrections of tetralogy of Fallot. A 26-31 year follow-up of 106 patients. Ann Surg. 1986 Oct;204(4):490-502. doi: 10.1097/00000658-198610000-00017. PMID: 3767482; PMCID: PMC1251326.

62: Schmidt W, Lang K. Life-threatening dysrhythmias in severe thioridazine poisoning treated with physostigmine and transient atrial pacing. Crit Care Med. 1997 Nov;25(11):1925-30. doi: 10.1097/00003246-199711000-00036. PMID: 9366781.

63: Heinroth KM, Kuhn C, Walper R, Busch I, Winkler M, Prondzinsky R. Akute Intoxikation mit dem beta 1-selektiven beta-Rezeptorenblocker Nebivolol in suizidaler Absicht [Acute beta 1-selective beta-receptor blocker nebivolol poisoning in attempted suicide]. Dtsch Med Wochenschr. 1999 Oct 22;124(42):1230-4. German. doi: 10.1055/s-2007-1024525. PMID: 10572531.

64: Thouny R, Benoit A, Gach O. L'image du mois. Pseudoanévrisme ventriculaire gauche secondaire à un traumatisme par balle [Image of the month: Ventricular pseudoaneurysm secondary to gunshot wound]. Rev Med Liege. 2014 Jul-Aug;69(7-8):409-11. French. PMID: 25158380.

65: Brucculeri M, Kaplan J, Lande L. Reversal of citalopram-induced junctional bradycardia with intravenous sodium bicarbonate. Pharmacotherapy. 2005 Jan;25(1):119-22. doi: 10.1592/phco.25.1.119.55630. PMID: 15767228.

66: Cherpanath TG, Geisler FE, van der Meer BJ, van Veer NE, Alings AM. Tentamen suicidii met sotalol: een proaritmische intoxicatie [Suicide attempt with sotalol: a proarrhythmic intoxication]. Ned Tijdschr Geneeskd. 2007 Oct 6;151(40):2214-8. Dutch. PMID: 17969573.

67: Tötterman KJ, Turto H, Pellinen T. Overdrive pacing as treatment of sotalol-induced ventricular tachyarrhythmias (torsade de pointes). Acta Med Scand Suppl. 1982;668:28-33. doi: 10.1111/j.0954-6820.1982.tb08519.x. PMID: 6963090.

68: Althoff H. Gewebereaktionen auf implantierte Herzschrittmacher [Cardiovascular tissue response to intracardiac pacemaking (author's transl)]. Med Klin. 1978 Oct 20;73(42):1468-76. German. PMID: 703676.

69: Lumpkin J, Watanabe AS, Rumack BH, Peterson RG. Phenothiazine-induced ventricular tachycardia following acute overdose. JACEP. 1979 Nov;8(11):476-8. doi: 10.1016/s0361-1124(79)80065-9. PMID: 315488.

70: Heesen H, Lahrtz H. Verlauf und Behandlung von schweren Digitalisintoxikationen in suizidaler Absicht [Treatment of severe digitalis-intoxication in suicidal attempt (author's transl)]. Med Klin. 1975 May 2;70(18):812-6. German. PMID: 1143171.

71: Tomás CC, Oliveira E, Sousa D, Uba-Chupel M, Furtado G, Rocha C, Teixeira A, Ferreira P, Alves C, Gisin S, Catarino E, Carvalho N, Coucelo T, Bonfim L, Silva C, Franco D, González JA, Jardim HG, Silva R, Baixinho CL, Presado MªH, Marques MªF, Cardoso ME, Cunha M, Mendes J, Xavier A, Galhardo A, Couto M, Frade JG, Nunes C, Mesquita JR, Nascimento MS, Gonçalves G, Castro C, Mártires A, Monteiro MªJ, Rainho C, Caballero FP, Monago FM, Guerrero JT, Monago RM, Trigo AP, Gutierrez ML, Milanés GM, Reina MG, Villanueva AG, Piñero AS, Aliseda IR, Ramirez FB, Ribeiro A, Quelhas A, Manso C, Caballero FP, Guerrero JT, Monago FM, Santos RB, Jimenez NR, Nuñez CG, Gomez IR, Fernandez MªJL, Marquez LA, Moreno AL, Huertas MªJT, Ramirez FB, Seabra D, Salvador MªC, Braga L, Parreira P, Salgueiro-Oliveira A, Arreguy-Sena C, Oliveira BF, Henriques MªA, Santos J, Lebre S, Marques A, Festas C, Rodrigues S, Ribeiro A, Lumini J, Figueiredo AG, Hernandez-Martinez FJ, Campi L, Quintana-Montesdeoca MªP, Jimenez-Diaz JF, Rodriguez-De-Vera BC, Parente A, Mata MªA, Pereira AMª, Fernandes A, Brás M, Pinto MªR, Parreira P, Basto ML, Rei AC, Mónico LM, Sousa G, Morna C, Freitas O, Freitas G, Jardim A, Vasconcelos R, Horta LG, Rosa RS, Kranz LF, Nugem RC, Siqueira MS, Bordin R, Kniess R, Lacerda JT, Guedes J, Machado I, Almeida S, Zilhão A, Alves H, Ribeiro Ó, Amaral AP, Santos A, Monteiro J, Rocha MªC, Cruz R, Amaral AP, Lourenço M, Rocha MªC, Cruz R, Antunes S, Mendonça V, Andrade I, Osório N, Valado A, Caseiro A, Gabriel A, Martins AC, Mendes F, Cabral L, Ferreira M, Gonçalves A, Luz TD, Luz L, Martins R, Morgado A, Vale-Dias ML, Porta-Nova R, Fleig TC, Reuter ÉM, Froemming MB, Guerreiro SL, Carvalho LL,

Guedelha D, Coelho P, Pereira A, Calha A, Cordeiro R, Gonçalves A, Certo A, Galvão A, Mata MªA, Welter A, Pereira E, Ribeiro S, Kretzer M, Jiménez-Díaz JF, Jiménez-Rodríguez C, Hernández-Martínez FJ, Rodríguez-De-Vera BDC, Marques-Rodrigues A, Coelho P, Bernardes T, Pereira A, Sousa P, Filho JG, Nazario N, Kretzer M, Amaral O, Garrido A, Veiga N, Nunes C, Pedro AR, Pereira C, Almeia A, Fernandes HM, Vasconcelos C, Sousa N, Reis VM, Monteiro MJ, Mendes R, Pinto IC, Pires T, Gama J, Preto V, Silva N, Magalhães C, Martins M, Duarte M, Paúl C, Martín I, Pinheiro AA, Xavier S, Azevedo J, Bento E, Marques C, Marques M, Macedo A, Pereira AT, Almeida JP, Almeida A, Alves J, Sousa N, Saavedra F, Mendes R, Maia AS, Oliveira MT, Sousa AR, Ferreira PP, Lopes LS, Santiago EC, Monteiro S, Jesus Â, Colaço A, Carvalho A, Silva RP, Cruz A, Ferreira A, Marques C, Figueiredo JP, Paixão S, Ferreira A, Lopes C, Moreira F, Figueiredo JP, Ferreira A, Ribeiro D, Moreira F, Figueiredo JP, Paixão S, Fernandes T, Amado D, Leal J, Azevedo M, Ramalho S, Mangas C, Ribeiro J, Gonçalves R, Nunes AF, Tuna AR, Martins CR, Forte HD, Costa C, Tenedório JA, Santana P, Andrade JA, Pinto JL, Campofiorito C, Nunes S, Carmo A, Kaliniczenco A, Alves B, Mendes F, Jesus C, Fonseca F, Gehrke F, Albuquerque C, Batista R, Cunha M, Madureira A, Ribeiro

O, Martins R, Madeira T, Peixoto-Plácido C, Santos N, Santos O, Bergland A, Bye A, Lopes C, Alarcão V, Goulão B, Mendonça N, Nicola P, Clara JG, Gomes J, Querido A, Tomás C, Carvalho D, Cordeiro M, Rosa MC, Marques A, Brandão D, Ribeiro Ó, Araújo L, Paúl C, Minghelli B, Richaud S, Mendes AL, Marta-Simões J, Trindade IA, Ferreira C, Carvalho T, Cunha M, Pinto-Gouveia J, Fernandes MC, Rosa RS, Nugem RC, Kranz LF, Siqueira MS, Bordin R, Martins AC, Medeiros A, Pimentel R, Fernandes A, Mendonça C, Andrade I, Andrade S, Menezes RL, Bravo R, Miranda M, Ugartemendia L, Tena JMª, Pérez-Caballero FL, Fuentes-Broto L, Rodríguez AB, Carmen B, Carneiro MA, Domingues JN, Paixão S, Figueiredo J, Nascimento VB, Jesus C, Mendes F, Gehrke F, Alves B, Azzalis L, Fonseca F, Martins AR, Nunes A, Jorge A, Veiga N, Amorim A, Silva A, Martinho L, Monteiro L, Silva R, Coelho C, Amaral O, Coelho I, Pereira C, Correia A, Rodrigues D, Marante N, Silva P, Carvalho S, Araujo AR, Ribeiro M, Coutinho P, Ventura S, Roque F, Calvo C, Reses M, Conde J, Ferreira A, Figueiredo J, Silva D, Seiça L, Soares R, Mourão R, Kraus T, Abreu AC, Padilha JM, Alves JM, Sousa P, Oliveira

M, Sousa J, Novais S, Mendes F, Pinto J, Cruz J, Marques A, Duarte H, Dixe MDA, Sousa P, Cruz I, Bastos F, Pereira F, Carvalho FL, Oliveira TT, Raposo VR, Rainho C, Ribeiro JC, Barroso I, Rodrigues V, Neves C, Oliveira TC, Oliveira B, Morais MªC, Baylina P, Rodrigues R, Azeredo Z, Vicente C, Dias H, Sim-Sim M, Parreira P, Salgueiro-Oliveira A, Castilho A, Melo R, Graveto J, Gomes J, Vaquinhas M, Carvalho C, Mónico L, Brito N, Sarroeira C, Amendoeira J, Cunha F, Cândido A, Fernandes P, Silva HR, Silva E, Barroso I, Lapa L, Antunes C, Gonçalves A, Galvão A, Gomes MªJ, Escanciano SR, Freitas M, Parreira P, Marôco J, Fernandes AR, Cabral C, Alves S, Sousa P, Ferreira A, Príncipe F, Seppänen UM, Ferreira M, Carvalhais M, Silva M, Ferreira M, Silva J, Neves J, Costa D, Santos B, Duarte S, Marques S, Ramalho S, Mendes I, Louro C, Menino E, Dixe M, Dias SS, Cordeiro M, Tomás C, Querido A, Carvalho D, Gomes J, Valim FC, Costa JO, Bernardes LG, Prebianchi H, Rosa MC, Gonçalves N, Martins MM, Kurcgant P, Vieira A, Bento S, Deodato S, Rabiais I, Reis L, Torres A, Soares S, Ferreira M, Graça P, Leitão C, Abreu R, Bellém F, Almeida A, Ribeiro-Varandas E, Tavares A, Frade JG, Henriques C, Menino E, Louro C, Jordão C, Neco S, Morais C, Ferreira P, Silva CR, Brito A, Silva A, Duarte H, Dixe MDA, Sousa P, Postolache G, Oliveira R, Moreira I, Pedro L, Vicente S, Domingos S, Postolache O, Silva D, Filho JG, Nazario N, Kretzer M, Schneider D, Marques FM, Parreira P, Carvalho C, Mónico LM, Pinto C, Vicente S, Breda SJ, Gomes JH, Melo R, Parreira P, Salgueiro A, Graveto J, Vaquinhas M, Castilho A, Jesus Â, Duarte N, Lopes JC, Nunes H, Cruz A, Salgueiro-Oliveira A, Parreira P, Basto ML, Braga LM, Ferreira A, Araújo B, Alves JM, Ferreira M, Carvalhais M, Silva M, Novais S, Sousa AS, Ferrito C, Ferreira PL, Rodrigues A, Ferreira M, Oliveira I, Ferreira M, Neves J, Costa D, Duarte S, Silva J, Santos B, Martins C, Macedo AP, Araújo O, Augusto C, Braga F, Gomes L, Silva MA, Rosário R, Pimenta L, Carreira D, Teles P, Barros T, Tomás C, Querido A, Carvalho D, Gomes J, Cordeiro M, Carvalho D, Querido A, Tomás C, Gomes J, Cordeiro M, Jácome C, Marques A, Capelas S, Hall A, Alves D, Lousada M, Loureiro MªH, Camarneiro A, Silva M, Mendes A, Pedreiro A, G.Silva A, Coelho ES, Melo F, Ribeiro F, Torres R, Costa R, Pinho T, Jácome C, Marques A, Cruz B, Seabra D, Carreiras D, Ventura M, Cruz X, Brooks D, Marques A, Pinto MR, Parreira P, Lima-Basto M, Neves M, Mónico LM, Bizarro C, Cunha M, Galhardo A, Margarida C, Amorim AP, Silva E, Cruz S, Padilha JM, Valente J, Guerrero JT, Caballero FP, Santos RB, Gonzalez EP, Monago FM, Ugalde LU, Vélez MM, Tena MJ, Guerrero JT, Bravo R, Pérez-Caballero FL, Becerra IA, Agudelo MªE, Acedo G, Bajo R, Malheiro I, Gaspar F, Barros L, Furtado G, Uba-Chupel M, Marques M, Rama L, Braga M, Ferreira JP, Teixeira AMª, Cruz J, Barbosa T, Simões Â, Coelho L, Rodrigues A, Jiménez-Díaz JF, Martinez-Hernandez F, Rodriguez-De-Vera B, Ferreira P, Rodrigues A, Ramalho A, Petrica J, Mendes P, Serrano J, Santo I, Rosado A, Mendonça P, Freitas K, Ferreira D, Brito A, Fernandes R, Gomes S, Moreira F, Pinho C, Oliveira R, Oliveira AI, Mendonça P, Casimiro AP, Martins P, Silva I, Evangelista D, Leitão C, Velosa F, Carecho N, Coelho L, Menino E, Dixe A, Catarino H, Soares F, Gama E, Gordo C, Moreira E, Midões C, Santos M, Machado S, Oliveira VP, Santos M, Querido A, Dixe A, Marques R, Charepe Z, Antunes A, Santos S, Rosa MC, Rosa MC, Marques SF, Minghelli B, CaroMinghelli E, Luís MªJ, Brandão T, Mendes P, Marinho D, Petrica J, Monteiro D, Paulo R, Serrano J, Santo I, Monteiro L, Ramalho F, Santos-Rocha R, Morgado S, Bento T, Sousa G, Freitas O, Silva I, Freitas G, Morna C, Vasconcelos R, Azevedo T, Soares S, Pisco J, Ferreira PP, Olszewer EO, Oliveira MT, Sousa AR, Maia AS, Oliveira ST, Santos E, Oliveira AI, Maia C, Moreira F, Santos J, Mendes MF, Oliveira RF, Pinho C, Barreira E, Pereira A, Vaz JA, Novo A, Silva LD, Maia B, Ferreira E, Pires F, Andrade R, Camarinha L, Silva LD, Maia B, Ferreira E, Pires F, Andrade R, Camarinha L, César AF, Poço M, Ventura D, Loura R, Gomes P, Gomes C, Silva C,

Melo E, Lindo J, Domingos J, Mendes Z, Poeta S, Carvalho T, Tomás C, Catarino H, Dixe MªA, Ramalho A, Rosado A, Mendes P, Paulo R, Garcia I, Petrica J, Rodrigues S, Meneses R, Afonso C, Faria L, Seixas A, Cordeiro M, Granjo P, Gomes JC, Souza NR, Furtado GE, Rocha SV, Silva P, Carvalho J, Morais MA, Santos S, Lebre P, Antunes A, Calha A, Xavier A, Cunha M, Pinto-Gouveia J, Alencar L, Cunha M, Madureira A, Cardoso I, Galhardo A, Daniel F, Rodrigues V, Luz L, Luz T, Ramos MR, Medeiros DC, Carmo BM, Seabra A, Padez C, Silva MC, Rodrigues A, Coelho P, Coelho A, Caminha M, Matheus F, Mendes E, Correia J, Kretzer M, Hernandez-Martinez FJ, Jimenez-Diaz JF, Rodriguez-De-Vera BC, Jimenez-Rodriguez C, Armas-Gonzalez Y, Rodrigues C, Pedroso R, Apolinário-Hagen J, Vehreschild V, Veloso M, Magalhães C, Cabral I, Ferraz M, Nave F, Costa E, Matos F, Pacheco J, Dias A, Pereira C, Duarte J, Cunha M, Silva D, Mónico LM, Alferes VR, Brêda MªSJ, Carvalho C, Parreira PM, Morais MªC, Ferreira P, Pimenta R, Boavida J, Pinto IC, Pires T, Silva C, Ribeiro M, Viega-Branco M, Pereira F, Pereira AMª, Almeida FM, Estevez GL, Ribeiro S, Kretzer MR, João PV, Nogueira P, Novais S, Pereira A, Carneiro L, Mota M, Cruz R, Santiago L, Fontes-Ribeiro C, Furtado G, Rocha SV, Coutinho AP, Neto JS, Vasconcelos LR, Souza NR, Dantas E, Dinis A, Carvalho S, Castilho P, Pinto-Gouveia J, Sarreira-Santos A, Figueiredo A, Medeiros-Garcia L, Seabra P, Rodrigues R, Morais MªC, Fernandes PO, Santiago C, Figueiredo MªH, Basto ML, Guimarães T, Coelho A, Graça A, Silva AM, Fonseca AR, Vale-Dias L, Minas B, Franco-Borges G, Simões C, Santos S, Serra A, Matos M, Jesus L, Tavares AS, Almeida A, Leitão C, Varandas E, Abreu R, Bellém F, Trindade IA, Ferreira C, Pinto-Gouveia J, Marta-Simões J, Amaral O, Miranda C, Guimarães P, Gonçalves R, Veiga N, Pereira C, Fleig TC, San-Martin EA, Goulart CL, Schneiders PB, Miranda NF, Carvalho LL, Silva AG, Topa J, Nogueira C, Neves S, Ventura R, Nazaré C, Brandão D, Freitas A, Ribeiro Ó, Paúl C, Mercê C, Branco M, Almeida P, Nascimento D, Pereira J, Catela D, Rafael H, Reis AC, Mendes A, Valente AR, Lousada M, Sousa D, Baltazar AL, Loureiro MªH, Oliveira A, Aparício J, Marques A, Marques A, Oliveira A, Neves J, Ayoub R, Sousa L, Marques-Vieira C, Severino S, José H, Cadorio I, Lousada M, Cunha M, Andrade D, Galhardo A, Couto M, Mendes F, Domingues C, Schukg S, Abrantes AM, Gonçalves AC, Sales T, Teixo R, Silva R, Estrela J, Laranjo M, Casalta-Lopes J, Rocha C, Simões PC, Sarmento-Ribeiro AB, Botelho MªF, Rosa MS, Fonseca V, Colaço D, Neves V, Jesus C, Hesse C, Rocha C, Osório N, Valado A, Caseiro A, Gabriel A, Svensson L, Mendes F, Siba WA, Pereira C, Tomaz J, Carvalho T, Pinto-Gouveia J, Cunha M, Duarte D, Lopes NV, Fonseca-Pinto R, Duarte D, Lopes NV, Fonseca-Pinto R, Martins AC, Brandão P, Martins L, Cardoso M, Morais N, Cruz J, Alves N, Faria P, Mateus A, Morouço P, Alves N, Ferreira N, Mateus A, Faria P, Morouço P, Malheiro I, Gaspar F, Barros L, Parreira P, Cardoso A, Mónico L, Carvalho C, Lopes A, Salgueiro-Oliveira A, Seixas A, Soares V, Dias T, Vardasca R, Gabriel J, Rodrigues S, Paredes H, Reis A, Marinho S, Filipe V, Lains J, Barroso J, Da Motta C, Carvalho CB, Pinto-Gouveia J, Peixoto E, Gomes AA, Costa V, Couto D, Marques DR, Leitão JA, Tavares J, Azevedo MH, Silva CF, Freitas J, Parreira P, Marôco J, Garcia-Gordillo MA, Collado-Mateo D, Chen G, Iezzi A, Sala JA, Parraça JA, Gusi N, Sousa J, Marques M, Jardim J, Pereira A, Simões S, Cunha M, Sardo P, Guedes J, Lindo J, Machado P, Melo E, Carvalho CB, Benevides J, Sousa M, Cabral J, Da Motta C, Pereira AT, Xavier S, Azevedo J, Bento E, Marques C, Carvalho R, Marques M, Macedo A, Silva AM, Alves J, Gomes AA, Marques DR, Azevedo MªH, Silva C, Mendes A, Lee HD, Spolaôr N, Oliva JT, Chung WF, Fonseca-Pinto R, Bairros K, Silva CD, Souza CA, Schroeder SS, Araújo E, Monteiro H, Costa R, Dias SS, Torgal J, Henriques CG, Santos L, Caceiro EF, Ramalho SA, Oliveira R, Afreixo V, Santos J, Mota P, Cruz A, Pimentel F, Marques R, Dixe MªA, Querido A, Sousa P, Benevides J, Da Motta C, Sousa M, Caldeira SN, Carvalho CB, Querido A, Tomás C, Carvalho D, Gomes J, Cordeiro M, Costa JO, Valim FC, Ribeiro LC, Charepe Z, Querido A, Figueiredo MªH, Aquino PS, Ribeiro SG, Pinheiro AB, Lessa PA, Oliveira MF, Brito LS, Pinto ÍN, Furtado AS, Castro RB, Aquino CQ, Martins ES, Pinheiro AB, Aquino PS, Oliveira LL, Pinheiro PC, Sousa CR, Freitas VA, Silva TM, Lima AS, Aquino CQ, Andrade KV, Oliveira CA, Vidal EF, Ganho-Ávila A, Moura-Ramos M, Gonçalves Ó, Almeida J, Silva A, Brito I, Amado J, Rodrigo A, Santos S, Gomes F, Rosa MC, Marques SF, Luís S, Cavalheiro L, Ferreira P, Gonçalves R, Lopes RS, Cavalheiro L, Ferreira P, Gonçalves R, Fiorin BH, Santos MS, Oliveira ES, Moreira RL, Oliveira EA, Filho BL, Palmeira L, Garcia T, Pinto-Gouveia J, Cunha M, Cardoso S, Palmeira L, Cunha M, Pinto-Gouveia J, Marta-Simões J, Mendes AL, Trindade IA, Oliveira S, Ferreira C, Mendes AL, Marta-Simões J, Trindade IA, Ferreira C, Nave F, Campos M, Gaudêncio I, Martins F, Ferreira L, Lopes N, Fonseca-Pinto R, Rodrigues R, Azeredo Z, Vicente C, Silva J, Sousa P, Marques R, Mendes I, Rodrigues R, Azeredo Z, Vicente C, Vardasca R, Marques AR, Seixas A, Carvalho R, Gabriel J, Ferreira PP, Oliveira MT, Sousa AR, Maia AS, Oliveira ST, Costa PO, Silva MM, Arreguy-Sena C, Alvarenga-Martins N, Pinto PF, Oliveira DC, Parreira PD, Gomes AT, Braga LM, Araújo O, Lage I, Cabrita J, Teixeira L, Marques R, Dixe MªA, Querido A, Sousa P, Silva S, Cordeiro E, Pimentel J, Ferro-Lebres V, Souza JA, Tavares M, Dixe MªA, Sousa P, Passadouro R, Peralta T, Ferreira C, Lourenço G, Serrano J, Petrica J, Paulo R, Honório S, Mendes P, Simões A, Carvalho L, Pereira A, Silva S, Sousa P, Padilha JM, Figueiredo D, Valente C, Marques A, Ribas P, Sousa J, Brandão F, Sousa C, Martins M, Sousa P, Marques R, Mendes F, Fernandes R, Martins E, Magalhães C, Araújo P, Grande C, Mata MªA, Vieitez JG, Bianchini B, Nazario N, Filho JG, Kretzer M, Costa T, Almeida A, Baffour G, Almeida A, Costa T, Baffour G, Azeredo Z, Laranjeira C, Guerra M, Barbeiro AP, Ferreira R, Lopes S, Nunes L, Mendes A, Martins J, Schneider D, Kretzer M, Magajewski F, Soares C, Marques A, Batista M, Castuera RJ, Mesquita H, Faustino A, Santos J, Honório S, Vizzotto BP, Frigo L, Pivetta HF, Sardo D, Martins C, Abreu W, Figueiredo MªC, Batista M, Jimenez-Castuera R, Petrica J, Serrano J, Honório S, Paulo R, Mendes P, Sousa P, Marques R, Faustino A, Silveira P, Serrano J, Paulo R, Mendes P, Honório S, Oliveira C, Bastos F, Cruz I, Rodriguez CK, Kretzer MR, Nazário NO, Cruz P, Vaz DC, Ruben RB, Avelelas F, Silva S, Campos MªJ, Almeida M, Gonçalves L, Antunes L, Sardo P, Guedes J, Simões J, Machado P,

Melo E, Cardoso S, Santos O, Nunes C, Loureiro I, Santos F, Alves G, Soar C, Marsi TO, Silva E, Pedrosa D, Leça A, Silva D, Galvão A, Gomes M, Fernandes P, Noné A, Combadão J, Ramalhete C, Figueiredo P, Caeiro P, Fontana KC, Lacerda JT, Machado PO, Borges R, Barbosa F, Sá D, Brunhoso G, Aparício G, Carvalho A, Garcia AP, Fernandes PO, Santos A, Veiga N, Brás C, Carvalho I, Batalha J, Glória M, Bexiga F, Coelho I, Amaral O, Pereira C, Pinho C, Paraíso N, Oliveira AI, Lima CF, Dias AP, Silva P, Espada M, Marques M, Pereira A, Pereira AMª, Veiga-Branco Mª, Pereira F, Ribeiro M, Lima V, Oliveira AI, Pinho C, Cruz G, Oliveira RF, Barreiros L, Moreira F, Camarneiro A, Loureiro MªH, Silva M, Duarte C, Jesus Â, Cruz A, Mota M, Novais S, Nogueira P, Pereira A, Carneiro L, João PV, Lima TM, Salgueiro-Oliveira A, Vaquinhas M, Parreira P, Melo R, Graveto J, Castilho A, Gomes JH, Medina MS, Blanco VG, Santos O, Lopes E, Virgolino A, Dinis A, Ambrósio S, Almeida I, Marques T, Heitor MªJ, Garcia-Gordillo MA, Collado-Mateo D, Olivares PR, Parraça JA, Sala JA, Castilho A, Graveto J, Parreira P, Oliveira A, Gomes JH, Melo R, Vaquinhas M, Cheio M, Cruz A, Pereira OR, Pinto S, Oliveira A, Manso MC, Sousa C, Vinha AF, Machado MªM, Vieira M, Fernandes B, Tomás T, Quirino D, Desouzart G, Matos R, Bordini M, Mouroço P, Matos AR, Serapioni M, Guimarães T, Fonseca V, Costa A, Ribeiro J, Lobato J, Martin IZ, Björklund A, Tavares AI, Ferreira P, Passadouro R, Morgado S, Tavares N, Valente J, Martins AC, Araújo P, Fernandes R, Mendes F, Magalhães C, Martins E, Mendes P, Paulo R, Faustino A, Mesquita H, Honório S, Batista M, Lacerda JT, Ortiga AB, Calvo MªC, Natal S, Pereira M, Ferreira M, Prata AR, Nelas P, Duarte J, Carneiro J, Oliveira AI, Pinho C, Couto C, Oliveira RF, Moreira F, Maia AS, Oliveira MT, Sousa AR, Ferreira PP, Souza GM, Almada LF, Conceição MA, Santiago EC, Rodrigues S, Domingues G, Ferreira I, Faria L, Seixas A, Costa AR, Jesus Â, Cardoso A, Meireles A, Colaço A, Cruz A, Vieira VL, Vincha KR, Cervato-Mancuso AMª, Faria M, Reis C, Cova MP, Ascenso RT, Almeida HA, Oliveira EG, Santana M, Pereira R, Oliveira EG, Almeida HA, Ascenso RT, Jesus R, Tapadas R, Tim-Tim C, Cezanne C, Lagoa M, Dias SS, Torgal J, Lopes J, Almeida H, Amado S, Carrão L, Cunha M, Saboga-Nunes L, Albuquerque C, Ribeiro O, Oliveira S, Morais MªC, Martins E, Mendes F, Fernandes R, Magalhães C, Araújo P, Pedro AR, Amaral O, Escoval A, Assunção V, Luís H, Luís L, Apolinário-Hagen J, Vehreschild V, Fotschl U, Lirk G, Martins AC, Andrade I, Mendes F, Mendonça V, Antunes S, Andrade I, Osório N, Valado A, Caseiro A, Gabriel A, Martins AC, Mendes F, Silva PA, Mónico LM, Parreira PM, Carvalho C, Carvalho C, Parreira PM, Mónico LM, Ruivo J, Silva V, Sousa P, Padilha JM, Ferraz V, Aparício G, Duarte J, Vasconcelos C, Almeida A, Neves J, Correia T, Amorim H, Mendes R, Saboga- Nunes L, Cunha M, Albuquerque C, Pereira ES, Santos LS, Reis AS, Silva HR, Rombo J, Fernandes JC, Fernandes P, Ribeiro J, Mangas C, Freire A, Silva S, Francisco I, Oliveira A, Catarino H, Dixe MªA, Louro MªC, Lopes S, Dixe A, Dixe MªA, Menino E, Catarino H, Soares F, Oliveira AP, Gordo S, Kraus T, Tomás C, Queirós P, Rodrigues T, Sousa P, Frade JG, Lobão C, Moura CB, Dreyer LC, Meneghetti V, Cabral PP, Pinto F, Sousa P, Esteves MªR, Galvão S, Tytgat I, Andrade I, Osório N, Valado A, Caseiro A, Gabriel A, Martins AC, Mendes F, Casas-Novas M, Bernardo H, Andrade I, Sousa G, Sousa AP, Rocha C, Belo P, Osório N, Valado A, Caseiro A, Gabriel A, Martins AC, Mendes F, Martins F, Pulido-Fuentes M, Barroso I, Cabral G, Monteiro MJ, Rainho C, Prado A, Carvalho YM, Campos M, Moreira L, Ferreira J, Teixeira A, Rama L, Campos M, Moreira L, Ferreira J, Teixeira A, Rama L. Proceedings of the 3rd IPLeiria's International Health Congress : Leiria, Portugal. 6-7 May 2016. BMC Health Serv Res. 2016 Jul 6;16 Suppl 3(Suppl 3):200. doi: 10.1186/s12913-016-1423-5. PMID: 27409075; PMCID: PMC4943498.

72: Wahl G, Bücking J, Hübner H, Schwartau M. Behandlung einer lebensbedrohlichen Digoxin-Intoxikation mit heterologen digoxinspezifischen Antikörperfragmenten [Treatment of life-threatening digoxin poisoning with heterologous digoxin-specific antibody fragments]. Dtsch Med Wochenschr. 1983 Sep 23;108(38):1441-3. German. doi: 10.1055/s-2008-1069765. PMID: 6884235.

73: Beck OA, Krämer KD, Hochrein H. Verlauf einer suizidalen Digoxin-Intoxikation mit Hyperkaliämie. Klinische Beobachtung auf einer internen Intensivstation [Suicidal digoxin intoxication with hyperkalaemia: report of a case (author's transl)]. Dtsch Med Wochenschr. 1974 Apr 12;99(15):756-64. German. doi: 10.1055/s-0028-1107839. PMID: 4834839.

74: Rampertaap MP. Depression in a geriatric patient: alleviation by insertion of a pacemaker. South Med J. 1986 Aug;79(8):1043-4. doi: 10.1097/00007611-198608000-00035. PMID: 3738582.

75: Chan W, Ikram H. A case of self-induced digoxin poisoning: with His bundle studies of the site of heart block. N Z Med J. 1976 Dec 8;84(577):443-6. PMID: 1071160.

76: Adamek R, Kostrzewa A, Klimaszyk D. Ciezkie zatrucia lekami nasercowymi wymagajace czasowej stymulacji endokawitarnej [Cardiac drugs severe intoxications requiring artificial pacemakers]. Przegl Lek. 2003;60(4):277-9. Polish. PMID: 14569903.

77: Pilcher J, Heely MK. The Birmingham (Lucas) pacemaker. An appraisal of its use. Br Heart J. 1971 May;33(3):375-82. doi: 10.1136/hrt.33.3.375. PMID: 5579154; PMCID: PMC458421.

78: Josephsen PG, Møgelvang JC, Schierbeck J. Nyere behandling af svaer digoxinforgiftning [The latest treatment of severe digoxin poisoning]. Ugeskr Laeger. 1987 Apr 27;149(18):1183-5. Danish. PMID: 3603743.

79: Stoel I, Hagemeijer F, Hilvering C. Blijvend totaal atrioventriculair block na amitriptyline-intoxicatie [Permanent total atrioventricular block after amitriptyline poisoning]. Ned Tijdschr Geneeskd. 1977 Jul 2;121(27):1096-9. Dutch. PMID: 882168.

80: Duttaroy A, Qian JF, Smith JS, Wang E. Up-regulated P21CIP1 expression is part of the regulation quantitatively controlling serum deprivation-induced apoptosis. J Cell Biochem. 1997 Mar 1;64(3):434-46. PMID: 9057101.

81: Łukasik-Głebocka M, Sieńko A, Klimaszyk D, Mańkowski W. Skuteczne zastosowanie stymulacji endokawitarnej w leczeniu zaburzeń przewodnictwa i rytmu serca w przebiegu zatrucia cisem pospolitym [Effective intracavitary pacemaking for Taxus baccata-induced cardiac conduction defects and arrhythmias]. Przegl Lek. 2007;64(4-5):298-300. Polish. PMID: 17724890.

82: Faivre G, Gilgenkrantz JM, Petitier H, Weiller M, Dodinot B. Possibilités et limites de l'entraînement électrosystolique dans les intoxications digitaliques et quinidiniques [Possibilities and limitations of electrosystolic pacing in digitalis and quinidine poisoning]. Arch Mal Coeur Vaiss. 1968 Oct;61(10):1373-85. French. PMID: 4974047.

83: Brunclík J, Jezek A. Některé psychiatricko-psychologické problémy pouzití kardiostimulátorů (pacemakerů [Some psychiatric--psychological problems concerning the use of cardiostimulators (author's transl)]. Cas Lek Cesk. 1973 Oct 5;112(40):1226-30. Czech. PMID: 4755298.

84: Gusak VK, Kuznetsov AS, Dziugan' SA. Povrezhdenie éndokardial'nogo élektroda s suitsidal'noĭ tsel'iu [Damage to an endocardial electrode with a suicidal intent]. Klin Khir (1962). 1992;(9-10):76. Russian. PMID: 1291804.

85: Kröll W, Matzer C, Schalk HV, Tscheliessnigg KH. Tiefe akzidentelle Hypothermie (24 degrees C). Eine Kasuistik [Deep accidental hypothermia (24 degrees C). A case report]. Anasth Intensivther Notfallmed. 1988 Dec;23(6):330-3. German. PMID: 3239731.

Supplement SF – Summary table complications of implanted cardiac devices

Device (group)	Complications (examples)
PM and/or ICDs	• Phrenic nerve stimulation[77,78] or diaphragmatic stimulation[79] (rare) / peripheral nerve injury[80]<br>• PM-Twiddler's syndrome[#81]<br>• Pacemaker Syndrome[+ 82] / Inadequate capture and/or sensing[83] and/or shocks[84,85] / HF[12]<br>• Fibrosis around the leads[83,86]<br>• Hematoma[87–89]<br>• Pneumothorax[89] / hematothorax[80]<br>• Tricuspid valve stenosis[90] (rare)<br>• Narrowing of inferior [91] or superior vena cava [92] (both rare) / subclavian vein thrombosis[80]<br>• Lead: fracture, infection, displacement, insulation injury[33], repositioning[80], encapsulation[36]<br>• Devise extrusion, Ulceration device pocket, lead or generator erosion, superficial phlebitis[89], wound / pocket infection [33,80]<br>• Endocarditis[12] / sepsis[80]<br>• Myocardial perforation, pericardial tamponade, post-implantation myocardial infarction, hypotension with resuscitation, electrical storm, renal insufficiency, coronary venous dissection, pulmonary edema, stroke, non-cerebral embolus[80]<br>• Death[80]
Impella®	• Hemolysis [93–96] (due to anatomic features[96], occurrence of hemolysis dependent on positioning [95,96])<br>• Wide-ranging swinging motion can cause severe hemolysis, too [97].<br>• Susceptible for dislocation[98]<br>• Access site bleeding[93] or other access site complications[56,93,99,100]<br>• Limb ischemia[93]<br>• Need for vascular intervention or aortic injury [93]<br>• Left ventricular perforation[93]<br>• Infection[101]<br>• Need for transfusion[93]

Table continued	
ECLS / ECMO / CPB	• Acute lung injury[66]<br>• Systemic inflammatory response[102]<br>• Thrombocytopenia / acquired von-Willebrand syndrome / thromboembolism / hemolysis / disseminated intravasal coagulation[103]<br>• Pump / oxygenator / circuit thrombosis[103]<br>• Therapy-associated bleeding such as gastrointestinal, central nervous bleeding, or bladder clots / need for transfusion / cannulation site bleeding / surgical site bleeding[103]<br>• Limb ischemia[104]<br>• Dissection / arteriovenous fistulae / pseudoaneurysm[104]
Long-term VADs (e.g., HeartMate3 or EXCOR) and total artificial hearts	• Bleeding (e.g., gastrointenstinal tract bleeding, central nervous system bleeding)[105]<br>• Thromboembolic events / pump thrombosis[105]<br>• Infections, especially drive line infections[105]<br>• Aortic regurgitation[105]<br>• Right ventricular failure[105]
IABP	• Balloon entrapment rupture[106]<br>• Thrombocytopenia[106]<br>• Thromboembolism[106]<br>• Infection[106]<br>• Dissection[106,107]<br>• Limb ischemia / Amputation[107]<br>• Hemorrhage / Bleeding / Hematoma / Pseudo-aneurysm[107]<br>• Mesenteric ischemia[107]<br>• Inadequate function due to inadequate setting of the triggering[108]

(#) Pacemaker-Twiddler's syndrome describes "coiling" of the leads due to movement of the aggregate in its pocket. (+) The term pacemaker syndrome describes symptoms due to inadequate timing of atrioventricular synchrony. Abbreviations are explained in supplement A.

Supplement SG – References used in the supplement

1. McDonagh, T. A. *et al.* 2021 ESC Guidelines for the diagnosis and treatment of acute and chronic heart failure: Developed by the Task Force for the diagnosis and treatment of acute and chronic heart failure of the European Society of Cardiology (ESC) With the special contribution of the Heart Failure Association (HFA) of the ESC. *Eur. Heart J.* **42**, 3599–3726 (2021).
2. Savarese, G. & Lund, L. H. Global Public Health Burden of Heart Failure. *Card Fail Rev* **3**, 7–11 (2017).
3. Savarese, G. *et al.* Global burden of heart failure: a comprehensive and updated review of epidemiology. *Cardiovasc. Res.* **118**, 3272–3287 (2023).
4. Martin, C. A. & Lambiase, P. D. Pathophysiology, diagnosis and treatment of tachycardiomyopathy. *Heart* **103**, 1543–1552 (2017).
5. Jackson, G., Gibbs, C. R., Davies, M. K. & Lip, G. Y. ABC of heart failure. Pathophysiology. *BMJ* **320**, 167–170 (2000).
6. Sanders, J. L., Koestenberger, M., Rosenkranz, S. & Maron, B. A. Right ventricular dysfunction and long-term risk of death. *Cardiovasc Diagn Ther* **10**, 1646–1658 (2020).
7. Unverferth, D. V. *et al.* Human myocardial histologic characteristics in congestive heart failure. *Circulation* **68**, 1194–1200 (1983).
8. Hartupee, J. & Mann, D. L. Neurohormonal activation in heart failure with reduced ejection fraction. *Nat. Rev. Cardiol.* **14**, 30–38 (2017).
9. Juillière, Y. *et al.* Heart failure with preserved ejection fraction: A systemic disease linked to multiple comorbidities, targeting new therapeutic options. *Arch. Cardiovasc. Dis.* **111**, 766–781 (2018).
10. Miyagi, C., Miyamoto, T., Karimov, J. H., Starling, R. C. & Fukamachi, K. Device-based treatment options for heart failure with preserved ejection fraction. *Heart Fail. Rev.* **26**, 749–762 (2021).
11. Lam, C. S. P. *et al.* Mortality associated with heart failure with preserved vs. reduced ejection fraction in a prospective international multi-ethnic cohort study. *Eur. Heart J.* **39**, 1770–1780 (2018).
12. Glaser, N., Persson, M., Dalén, M. & Sartipy, U. Long-term Outcomes Associated With Permanent Pacemaker Implantation After Surgical Aortic Valve Replacement. *JAMA Netw Open* **4**, e2116564–e2116564 (2021).
13. Eberhardt, F. *et al.* Long term complications in single and dual chamber pacing are influenced by surgical experience and patient morbidity. *Heart* **91**, 500–506 (2005).
14. Kennergren, C., Bjurman, C., Wiklund, R. & Gäbel, J. A single-centre experience of over one thousand lead extractions. *Europace* **11**, 612–617 (2009).
15. Borne, R. T. *et al.* Trends in Use of Single- vs Dual-Chamber Implantable Cardioverter-Defibrillators Among Patients Without a Pacing Indication, 2010-2018. *JAMA Netw Open* **5**, e223429–e223429 (2022).

16. Bänsch, D., Schneider, R., Akin, I. & Nienaber, C. A. A new single chamber implantable defibrillator with atrial sensing: a practical demonstration of sensing and ease of implantation. *J. Vis. Exp.* (2012) doi:10.3791/3750.

17. Edelson, J. B. *et al.* An Increasing Burden of Disease: Emergency Department Visits Among Patients With Ventricular Assist Devices From 2010 to 2017. *J. Am. Heart Assoc.* **10**, e018035 (2021).

18. Gribbin, G. M., Mccomb, J. M. & Bexton, R. S. Ventricular pacemaker upgrade: experience, complications, and recommendations. *Heart* **80**, 420–420 (1998).

19. Kini, V. *et al.* Appropriateness of primary prevention implantable cardioverter-defibrillators at the time of generator replacement: are indications still met? *J. Am. Coll. Cardiol.* **63**, 2388–2394 (2014).

20. Gama, F. *et al.* Implantable Cardioverter–Defibrillators in Trials of Drug Therapy for Heart Failure: A Systematic Review and Meta-Analysis. *J. Am. Heart Assoc.* **9**, e015177 (2020).

21. Zeppenfeld, K. *et al.* 2022 ESC Guidelines for the management of patients with ventricular arrhythmias and the prevention of sudden cardiac death: Developed by the task force for the management of patients with ventricular arrhythmias and the prevention of sudden cardiac death of the European Society of Cardiology (ESC) Endorsed by the Association for European Paediatric and Congenital Cardiology (AEPC). *Eur. Heart J.* ehac262 (2022).

22. Mellor, G. J. & Behr, E. R. Cardiac channelopathies: diagnosis and contemporary management. *Heart* **107**, 1092–1099 (2021).

23. Boulet, J. *et al.* Current Review of Implantable Cardioverter Defibrillator Use in Patients With Left Ventricular Assist Device. *Curr. Heart Fail. Rep.* **16**, 229–239 (2019).

24. Epstein, A. E. *et al.* Implantable cardioverter-defibrillator prescription in the elderly. *Heart Rhythm* **6**, 1136–1143 (2009).

25. Aktaş, M. K. *et al.* Survival After Implantable Cardioverter-Defibrillator Shocks. *J. Am. Coll. Cardiol.* **77**, 2453–2462 (2021).

26. Russo, V. *et al.* Lead Abandonment and Subcutaneous Implantable Cardioverter-Defibrillator (S-ICD) Implantation in a Cohort of Patients With ICD Lead Malfunction. *Front Cardiovasc Med* **8**, 692943 (2021).

27. Lopez, J. A. Implantable cardioverter defibrillator lead placement in the middle cardiac vein after tricuspid valve surgery. *Europace* **14**, 853–858 (2012).

28. Cannon, B. C. *et al.* Innovative techniques for placement of implantable cardioverter-defibrillator leads in patients with limited venous access to the heart. *Pacing Clin. Electrophysiol.* **29**, 181–187 (2006).

29. Duray, G. Z., Schmitt, J., Cicek-Hartvig, S., Hohnloser, S. H. & Israel, C. W. Complications leading to surgical revision in implantable cardioverter defibrillator patients: comparison of patients with single-chamber, dual-chamber, and biventricular devices. *Europace* **11**, 297–302 (2009).

30. Franco Fernandez, I., Aroca Peinado, A., Merino Llorens, J. L. & Al-Razzo, O. CTR-D without the left lead vs single/dual chamber ICD implantation: a cost saving strategy to avoid future upgrading of the latter? *Europace* **24**, euac053.509 (2022).

31. Kaya, E., Rassaf, T. & Wakili, R. Subcutaneous ICD: Current standards and future perspective. *Int J Cardiol Heart Vasc* **24**, 100409 (2019).

32. Higgins, S. L. *et al.* The first year experience with the dual chamber ICD. *Pacing Clin. Electrophysiol.* **23**, 18–25 (2000).

33. Borek, P. P. & Wilkoff, B. L. Pacemaker and ICD leads: strategies for long-term management. *J. Interv. Card. Electrophysiol.* **23**, 59–72 (2008).

34. Gemein, C., Haj, M. & Schmitt, J. Combining an subcutaneous ICD and a pacemaker with abdominal device location and bipolar epicardial left ventricular lead: first-in-man approach. *Europace* **18**, 1279 (2016).

35. Bernstein, A. D. *et al.* North American Society of Pacing and Electrophysiology policy statement. The NASPE/BPEG defibrillator code. *Pacing Clin. Electrophysiol.* **16**, 1776–1780 (1993).

36. Keiler, J. *et al.* Neointimal fibrotic lead encapsulation - Clinical challenges and demands for implantable cardiac electronic devices. *J. Cardiol.* **70**, 7–17 (2017).

37. Aggarwal, R. K., Connelly, D. T., Ray, S. G., Ball, J. & Charles, R. G. Early complications of permanent pacemaker implantation: no difference between dual and single chamber systems. *Heart* **73**, 571–575 (1995).

38. Ezzat, V. A. *et al.* A systematic review of ICD complications in randomised controlled trials versus registries: is our “real-world” data an underestimation? *Open Heart* **2**, e000198 (2015).

39. El-Chami, M. F. *et al.* Leadless pacemakers reduce risk of device-related infection: Review of the potential mechanisms. *Heart Rhythm* **17**, 1393–1397 (2020).

40. Kelle, A. M. *et al.* Dual-chamber epicardial pacing in neonates with congenital heart block. *J. Thorac. Cardiovasc. Surg.* **134**, 1188–1192 (2007).

41. Batra, A. S. & Balaji, S. Post operative temporary epicardial pacing: When, how and why? *Ann. Pediatr. Cardiol.* **1**, 120–125 (2008).

42. Harrigan, R. A., Chan, T. C., Moonblatt, S., Vilke, G. M. & Ufberg, J. W. Temporary transvenous pacemaker placement in the Emergency Department. *J. Emerg. Med.* **32**, 105–111 (2007).

43. Segar, D. E., Maldonado, J. R., Brown, C. G. & Law, I. H. Transvenous Versus Epicardial Pacing in Fontan Patients. *Pediatr. Cardiol.* **39**, 1484–1488 (2018).

44. Winter, S., Fehske, W., Steven, D. & Sultan, A. Supplement: Perspektiven der Kardiologie - Kabellose Herzschrittmacher: Erfahrungen und Ausblick. *Deutsches Ärzteblatt* **114**, [12] (2/2017).

45. Chauhan, A. *et al.* Early complications after dual chamber versus single chamber pacemaker implantation. *Pacing Clin. Electrophysiol.* **17**, 2012–2015 (1994).

46. Le Franc, P. *et al.* Triple chamber pacemaker for end-stage heart failure in a patient with a previously implanted automatic defibrillator. *Pacing Clin. Electrophysiol.* **21**, 1672–1675 (1998).

47. Glikson, M. *et al.* 2021 ESC Guidelines on cardiac pacing and cardiac resynchronization therapy: Developed by the Task Force on cardiac pacing and cardiac resynchronization therapy of the European Society of Cardiology (ESC) With the special contribution of the European Heart Rhythm Association (EHRA). *Eur. Heart J.* **42**, 3427–3520 (2021).

48. Butter, C., Georgi, C. & Stockburger, M. Optimal CRT Implantation-Where and How To Place the Left-Ventricular Lead? *Curr. Heart Fail. Rep.* **18**, 329–344 (2021).

49. Möllmann, H. *et al.* Desynchronization: a novel model to induce heart failure. *Thorac. Cardiovasc. Surg.* **57**, 441–448 (2009).

50. Katbeh, A. *et al.* Cardiac Resynchronization Therapy Optimization: A Comprehensive Approach. *Cardiology* **142**, 116–128 (2019).

51. Daubert, C., Gadler, F., Mabo, P. & Linde, C. Pacing for hypertrophic obstructive cardiomyopathy: an update and future directions. *Europace* **20**, 908–920 (2017).

52. Bernstein, A. D. *et al.* The NASPE/BPEG generic pacemaker code for antibradyarrhythmia and adaptive-rate pacing and antitachyarrhythmia devices. *Pacing Clin. Electrophysiol.* **10**, 794–799 (1987).

53. Bernstein, A. D. *et al.* The revised NASPE/BPEG generic code for antibradycardia, adaptive-rate, and multisite pacing. *Pacing Clin. Electrophysiol.* **25**, 260–264 (2002).

54. Han, J. & Trumble, D. R. Cardiac Assist Devices: Early Concepts, Current Technologies, and Future Innovations. *Bioengineering (Basel)* **6**, (2019).

55. Vanderpluym, C. J., Fynn-Thompson, F. & Blume, E. D. Ventricular assist devices in children: progress with an orphan device application. *Circulation* **129**, 1530–1537 (2014).

56. Burzotta, F. *et al.* Impella ventricular support in clinical practice: Collaborative viewpoint from a European expert user group. *Int. J. Cardiol.* **201**, 684–691 (2015).

57. Lafçı, G., Budak, A. B., Yener, A. Ü. & Cicek, O. F. Use of Extracorporeal Membrane Oxygenation in Adults. *Heart Lung Circ.* **23**, 10–23 (2014).

58. Seiler, F., Trudzinski, F. C. & Hennemann, K. The Homburg lung: efficacy and safety of a minimal-invasive pump-driven device for veno-venous extracorporeal carbon dioxide removal. *ASAIO* (2017).

59. Meuris, B., Arnout, J., Vlasselaers, D. & Schetz, M. Long-term management of an implantable left ventricular assist device using low molecular weight heparin and antiplatelet therapy: a possible alternative to oral …. *Artificial* (2007).

60. Kremer, J. *et al.* Long-term paracorporeal pulsatile mechanical circulatory support in adolescent and adult patients. *Interact. Cardiovasc. Thorac. Surg.* **35**, (2022).

61. Kannojiya, V., Das, A. K. & Das, P. K. Comparative assessment of different versions of axial and centrifugal LVADs: A review. *Artif. Organs* **45**, 665–681 (2021).

62. Kurihara, C., Kawabori, M. & Sugiura, T. Bridging to a long-term ventricular assist device with short-term mechanical circulatory support. *Artificial* (2018).

63. Bertoldi, L. F., Delmas, C., Hunziker, P. & Pappalardo, F. Escalation and de-escalation of mechanical circulatory support in cardiogenic shock. *Eur. Heart J. Suppl.* **23**, A35–A40 (2021).

64. Marbach, J. A., Chweich, H., Miyashita, S. & Kapur, N. K. Temporary mechanical circulatory support devices: updates from recent studies. *Curr. Opin. Cardiol.* **36**, 375–383 (2021).

65. Pineton de Chambrun, M., Bréchot, N. & Combes, A. Mechanical circulatory devices in acute heart failure. *Curr. Opin. Crit. Care* **24**, 286–291 (2018).

66. Boulate, D. *et al.* Acute lung injury after mechanical circulatory support implantation in patients on extracorporeal life support: an unrecognized problem. *Eur. J. Cardiothorac. Surg.* **44**, 544–9; discussion 549-50 (2013).

67. Nersesian, G. *et al.* Temporary mechanical circulatory support for refractory heart failure: the German Heart Center Berlin experience. *Ann Cardiothorac Surg* **8**, 76–83 (2019).

68. Mehra, M. R. *et al.* Primary results of long-term outcomes in the MOMENTUM 3 pivotal trial and continued access protocol study phase: a study of 2200 HeartMate 3 left ventricular assist device implants. *Eur. J. Heart Fail.* **23**, 1392–1400 (2021).

69. Goldstein, D. J. *et al.* Association of clinical outcomes with left ventricular assist device use by bridge to transplant or destination therapy intent: the multicenter study of MagLev technology in patients undergoing mechanical circulatory support therapy with HeartMate 3 (MOMENTUM 3) randomized clinical trial. *JAMA cardiology* **5**, 411–419 (2020).

70. Jakovljevic, D. G. *et al.* Left Ventricular Assist Device as a Bridge to Recovery for Patients With Advanced Heart Failure. *J. Am. Coll. Cardiol.* **69**, 1924–1933 (2017).

71. Wong, A. S. K. & Sin, S. W. C. Short-term mechanical circulatory support (intra-aortic balloon pump, Impella, extracorporeal membrane oxygenation, TandemHeart): a review. *Ann Transl Med* **8**, 829 (2020).

72. Cochran, R. P., Starkey, T. D., Panos, A. L. & Kunzelman, K. S. Ambulatory intraaortic balloon pump use as bridge to heart transplant. *Ann. Thorac. Surg.* **74**, 746–51; discussion 751-2 (2002).

73. Lyon, A. R., Samara, M. A. & Feldman, D. S. Cardiac contractility modulation therapy in advanced systolic heart failure. *Nat. Rev. Cardiol.* **10**, 584–598 (2013).

74. Koplan, B. A. *et al.* Implantable Loop Recorder Monitoring and the Incidence of Previously Unrecognized Atrial Fibrillation in Patients on Hemodialysis. *Kidney Int Rep* **7**, 189–199 (2022).

75. Elena, V. & Brignole, M. Implantable loop recorders in Clinical practice. *escardio.org*.

76. Cotter, P. E. *et al.* Incidence of atrial fibrillation detected by implantable loop recorders in unexplained stroke. *Neurology* **80**, 1546–1550 (2013).

77. Dalex, M., Malezieux, A., Parent, T., Zekry, D. & Serratrice, C. Phrenic nerve stimulation, a rare complication of pacemaker: A case report. *Medicine* **100**, e25060 (2021).

78. Sprinkle, J. D., Takaro, T. & Scott, S. M. Phrenic Nerve Stimulation as a Complication of the Implantable Cardiac Pacemaker. *Circulation* **28**, 114–116 (1963).

79. Shah, R. & Qualls, Z. Diaphragmatic stimulation caused by cardiac resynchronization treatment. *CMAJ* **188**, E239 (2016).

80. Lee, D. S. *et al.* Evaluation of early complications related to De Novo cardioverter defibrillator implantation insights from the Ontario ICD database. *J. Am. Coll. Cardiol.* **55**, 774–782 (2010).

81. Bayliss, C. E., Beanlands, D. S. & Baird, R. J. The pacemaker-twiddler's syndrome: a new complication of implantable transvenous pacemakers. *Can. Med. Assoc. J.* **99**, 371–373 (1968).

82. Ellenbogen, K. A., Gilligan, D. M., Wood, M. A., Morillo, C. & Barold, S. S. The pacemaker syndrome -- a matter of definition. *Am. J. Cardiol.* **79**, 1226–1229 (1997).

83. Kiviniemi, M. S., Pirnes, M. A., Eränen, H. J., Kettunen, R. V. & Hartikainen, J. E. Complications related to permanent pacemaker therapy. *Pacing Clin. Electrophysiol.* **22**, 711–720 (1999).

84. Haeberlin, A. *et al.* Sense-B-noise: an enigmatic cause for inappropriate shocks in subcutaneous implantable cardioverter defibrillators. *Europace* **25**, 767–774 (2022).

85. Koneru, J. N., Swerdlow, C. D., Wood, M. A. & Ellenbogen, K. A. Minimizing Inappropriate or "Unnecessary" Implantable Cardioverter-Defibrillator Shocks. *Circ. Arrhythm. Electrophysiol.* **4**, 778–790 (2011).

86. Robboy, S. J., Harthorne, J. W., Leinbach, R. C., Sanders, C. A. & Austen, W. G. Autopsy findings with permanent pervenous pacemakers. *Circulation* **39**, 495–501 (1969).

87. Nichols, C. I. & Vose, J. G. Incidence of bleeding-related complications during primary implantation and replacement of cardiac implantable electronic devices. *J. Am. Heart Assoc.* **6**, (2017).

88. Przybylski, A. *et al.* Bleeding complications after pacemaker or cardioverter-defibrillator implantation in patients receiving dual antiplatelet therapy: Results of a prospective, two-centre registry. *Neth. Heart J.* **18**, 230–235 (2010).

89. Carrión-Camacho, M. R., Marín-León, I., Molina-Doñoro, J. M. & González-López, J. R. Safety of Permanent Pacemaker Implantation: A Prospective Study. *J. Clin. Med. Res.* **8**, (2019).

90. Heaven, D. J., Henein, M. Y. & Sutton, R. Pacemaker lead related tricuspid stenosis: a report of two cases. *Heart* **83**, 351–352 (2000).

91. Shimada, Y. *et al.* An operative case of inferior vena cava stenosis due to fibrosis around permanent pacemaker leads. *Pacing Clin. Electrophysiol.* **25**, 223–225 (2002).

92. Mazzetti, H., Dussaut, A., Tentori, C., Dussaut, E. & Lazzari, J. O. Superior vena cava occlusion and/or syndrome related to pacemaker leads. *Am. Heart J.* **125**, 831–837 (1993).

93. Ancona, M. B. *et al.* Device-related complications after Impella mechanical circulatory support implantation: an IMP-IT observational multicentre registry substudy. *Eur Heart J Acute Cardiovasc Care* **10**, 999–1006 (2021).

94. Salas de Armas, I. *et al.* Surgically Implanted Impella Device for Patients on Impella CP Support Experiencing Refractory Hemolysis. *ASAIO J.* **68**, e251–e255 (2022).

95. Roberts, N., Chandrasekaran, U., Das, S., Qi, Z. & Corbett, S. Hemolysis associated with Impella heart pump positioning: In vitro hemolysis testing and computational fluid dynamics modeling. *Int. J. Artif. Organs* 391398820909843 (2020).

96. Nakamura, M. *et al.* Impact of the angle between aortic and mitral annulus on the occurrence of hemolysis during Impella support. *J. Artif. Organs* **23**, 207–213 (2020).

97. Nakao, Y. *et al.* Impella 5.0 Mechanical Assist Device Catheter-Induced Severe Hemolysis Due to Giant Swinging Motion - New Concern in Impella Usage. *Circ. J.* **83**, 2080 (2019).

98. Mathur Moses, Mohmand-Borkowski Adam, Harper Mariko & Kritzer Gordon. Percutaneous Salvage of an Impella Pretzel. *JACC: Case Reports* **1**, 254–255 (2019).

99. Lewin, D. *et al.* Complications related to the access site after transaxillary implantation of a microaxial left ventricular assist device. *J. Heart Lung Transplant.* **42**, 679–687 (2023).

100. Johannsen, L. *et al.* Access site complications following Impella-supported high-risk percutaneous coronary interventions. *Sci. Rep.* **9**, 1–6 (2019).

101. Lanfear, A. T. *et al.* Fevers after Impella Implantation are Common but Rarely Clinically Significant. *J. Heart Lung Transplant.* **40**, S412–S413 (2021).

102. Day, J. R. S. & Taylor, K. M. The systemic inflammatory response syndrome and cardiopulmonary bypass. *Int. J. Surg.* **3**, 129–140 (2005).

103. Murphy, D. A. *et al.* Extracorporeal Membrane Oxygenation—Hemostatic Complications. *Transfus. Med. Rev.* **29**, 90–101 (2015).

104. Stulak, J. M. *et al.* ECMO cannulation controversies and complications. *Semin. Cardiothorac. Vasc. Anesth.* **13**, 176–182 (2009).

105. Jezovnik, M. K., Gregoric, I. D. & Poredos, P. Medical complications in patients with LVAD devices. *European Society of Cardiology* **14**, (2017).

106. Parissis, H., Soo, A. & Al-Alao, B. Intra aortic balloon pump: literature review of risk factors related to complications of the intraaortic balloon pump. *J. Cardiothorac. Surg.* **6**, 147 (2011).

107. de Jong, M. M. *et al.* Vascular complications following intra-aortic balloon pump implantation: an updated review. *Perfusion* **33**, 96–104 (2018).

108. Maccioli, G. A., Lucas, W. J. & Norfleet, E. A. The intra-aortic balloon pump: a review. *J. Cardiothorac. Anesth.* **2**, 365–373 (1988).
